# Estimating the loss of economic predictability from aggregating firm-level production networks

**DOI:** 10.1093/pnasnexus/pgae064

**Published:** 2024-02-17

**Authors:** Christian Diem, András Borsos, Tobias Reisch, János Kertész, Stefan Thurner

**Affiliations:** Complexity Science Hub Vienna, Vienna A-1080, Austria; Institute for Finance, Banking and Insurance, Vienna University of Economics and Business, Vienna A-1020, Austria; Complexity Science Hub Vienna, Vienna A-1080, Austria; Department of Financial Systems Analysis, Central Bank of Hungary, Budapest 1013, Hungary; Department of Network and Data Science, Central European University, Vienna A-1100, Austria; Complexity Science Hub Vienna, Vienna A-1080, Austria; Section for Science of Complex Systems, CeMDAS, Medical University of Vienna, Vienna A-1090, Austria; Complexity Science Hub Vienna, Vienna A-1080, Austria; Department of Network and Data Science, Central European University, Vienna A-1100, Austria; Complexity Science Hub Vienna, Vienna A-1080, Austria; Section for Science of Complex Systems, CeMDAS, Medical University of Vienna, Vienna A-1090, Austria; Santa Fe Institute, Santa Fe, NM 85701, USA

**Keywords:** production networks, supply-chain disruptions, shock propagation, resilience

## Abstract

To estimate the reaction of economies to political interventions or external disturbances, input–output (IO) tables—constructed by aggregating data into industrial sectors—are extensively used. However, economic growth, robustness, and resilience crucially depend on the detailed structure of nonaggregated firm-level production networks (FPNs). Due to nonavailability of data, little is known about how much aggregated sector-based and detailed firm-level-based model predictions differ. Using a nearly complete nationwide FPN, containing 243,399 Hungarian firms with 1,104,141 supplier–buyer relations, we self-consistently compare production losses on the aggregated industry-level production network (IPN) and the granular FPN. For this, we model the propagation of shocks of the same size on both, the IPN and FPN, where the latter captures relevant heterogeneities within industries. In a COVID-19 inspired scenario, we model the shock based on detailed firm-level data during the early pandemic. We find that using IPNs instead of FPNs leads to an underestimation of economic losses of up to 37%, demonstrating a natural limitation of industry-level IO models in predicting economic outcomes. We ascribe the large discrepancy to the significant heterogeneity of firms within industries: we find that firms within one sector only sell 23.5% to and buy 19.3% from the same industries on average, emphasizing the strong limitations of industrial sectors for representing the firms they include. Similar error levels are expected when estimating economic growth, CO_2_ emissions, and the impact of policy interventions with industry-level IO models. Granular data are key for reasonable predictions of dynamical economic systems.

**Significance Statement**

Economic processes, such as economic growth or how crises affect the economy, depend on how firms are connected to each other in supply-chain networks. These have hitherto not been accessible. Economic systems are typically analyzed on the aggregate level using industry-level input–output tables that specify how much one industry buys/sells to/from others. This aggregation necessarily leads to errors when predicting effects of supply-chain disturbances, political interventions, or economic change. Using a unique dataset of the detailed firm-level supply network of an entire country, we quantify these errors and conclude that models based on aggregated data severely misestimate losses in economic output. We demonstrate the necessity of using firm-level data for reasonable modeling how disruptions spread through supply chains.

## Introduction

Supplier–buyer relationships between economic agents such as firms—the production network (PN)—constitute the backbone of every economy. The network structure of PNs, and their ability to undergo dynamical change are crucial for understanding and predicting central economic processes, including innovation (1, 2), growth (3, 4), development (5), creation of CO_2_ emissions (6–8), resilience (9–11), and how economies respond to severe crises (12–14), policy interventions (15), or how shocks are amplified (16–19). The way PNs operate, restructure over time, or how they adapt to changes in global markets, their local environments, and to societal change is largely determined by decisions made by firms. These decisions include what and how much to produce, the combination of material inputs and technology, prices, salaries, who to hire, how to finance production and innovation, and—importantly—how to react to change, crises, and shocks like, e.g. loss of essential suppliers, natural disasters, wars, pandemics, trade wars, or economic sanctions. The decisions of firms subject PNs to constant change. As a consequence, also the associated systemic properties of the economy change, such as its efficiency, robustness, or resilience.

Studying *firm-level production networks* (FPNs) has been almost impossible until recently, when large-scale FPNs that include (almost) all firms and (almost) all their supply links have become available for countries such as Japan (20) (1.1 million firms, 5.5 million links), Belgium (21) (0.8 million firms, 17.3 million links), or Hungary (22) (0.25 million firms, 1.1 million links); for a review, see Ref. (23). Subsequently, new methods were developed to reconstruct FPNs (24–27). Firm-to-firm supply network data were used for studying shock propagation after natural disasters (28, 29), analyzing interactions of the financial system with the FPN (30–33), and quantifying the systemic risk contributions of individual firms in an economy have become possible (19). Insights that became accessible with the new data include measuring the prevalence of indirect exposures of firms to imports and exports through FPN links (34), an explanation of the role of the FPN for firm-size heterogeneity (35), revealing how price changes (inflation) propagate through the FPN (36), and an estimation of how gaining or losing suppliers affect the input costs of firms (37). Further availability of large-scale FPN data paired with the development of adequate economic models will help in improving economic policy (38).

Apart from these developments, the scientific understanding of PNs is still largely based on highly aggregated *industry-level production network* (IPN) data. IPNs are widely accessible in the form of *input–output tables* (IOTs) that record how the output of one entire industry enters as a production input into other industries. Typically, the dimensionality of IOTs ranges from 56 (2016 release of the world input–output database (39, 40)) to 405 sectors (US benchmark input–output statistics (41)). Up to now, industry-level IOTs remain a cornerstone of economic research and modeling even though IPNs (such as IOTs) are highly aggregated representations of the economy and do not capture the details of the intricate supply-chain relations between firms. Aggregating FPNs containing millions of firms to IPNs consisting of a few dozens of industries leads to a massive loss of information on the network structure of production and possibly to substantial biases that are known to occur even when aggregating (the already aggregated) IOTs (42–44). The aim of this paper is to demonstrate that these details that manifest themselves in significant inhomogeneities are often essential; their omission can be a source of considerable errors in economic predictions. Our demonstration focuses on how economic shocks affect the agents of an economy. The dynamical propagation of a shock depends on the detailed structure of the PN (29, 45, 46), as firms that fail might be essential suppliers or customers of firms that have to lower or stop their production as a consequence (47, 48). Hence, production disruptions can show cascading dynamics, similarly to financial contagion (49–52). To specify the necessary notation, we start with the following example.

The IPN consisting of *m* industries is represented by the weighted directed adjacency matrix, *Z*, where, a link, $Z_{kl}$, denotes the sales of goods or services (price times quantity) from industry *k* to industry *l* for a given time period. Figure 1A shows an example, *Z*, with m=5 industry sectors, where, e.g. industry 3 buys inputs needed for its production process from industry 2 and sells its output to sectors 1 and 5. Colors represent the different industries and link weights indicate sales volume. Figure 1B shows the underlying FPN, *W*, with n=11 firms. A link, $W_{ij}$, denotes the sales of firm *i* to firm *j* for the same time period. Every firm, *i*, belongs to one of the *m* industries, specified by the $i^{th}$ element of the industry classification vector, *p*, where, $p_{i}\in \{1,2,\ldots ,m\}$. In the example, firm *i* within industry *k* ($p_{i}=k$) is denoted by $f_{i}^{k}$, and e.g. firms $f_{3}^{2},f_{4}^{2}$, and $f_{5}^{2}$ of sector 2 sell to firms $f_{6}^{3}$ and $f_{7}^{3}$ of sector 3. Note that for simplicity in the example network in Fig. 1B and C, we set the weights of all existing links to 1, i.e. $W_{ij}=1$. Due to data constraints, we assume that each firm *i* only produces one good (or product), corresponding to its industry classification, $p_{i}$, as in Refs. (19, 28, 53). We construct the IPN, *Z*, by aggregating all product flows between firms from the respective industries, e.g. $Z_{23}=W_{36}+W_{46}+W_{47}+W_{57}$ and more generally $Z_{kl}=\sum_{i=1}^{n}\sum_{\;j=1}^{n}W_{ij}\delta_{\;p_{i},k}\delta_{\;p_{j},l}$.^a^ In our example, this yields the intersector link weights, $Z_{23}=4$, $Z_{31}=Z_{35}=Z_{41}=2$, and $Z_{45}=1$. The total number of sales of firm *i* to all other firms in the FPN are measured by its *out-strength*, $s_{i}^{\mathrm{out}}=\sum_{\;j=1}^{n}W_{ij}$, e.g., in our example $s_{3}^{\mathrm{out}}=1$ and $s_{6}^{\mathrm{out}}=2$. It is a proxy for firm *i*’s output (amount produced). The *in-strength*, $s_{i}^{\text{i}n}=\sum_{\;j=1}^{n}W_{\;ji}$, represents all purchases of *i* from other firms, e.g. in our example $s_{6}^{\text{i}n}=2$ and $s_{10}^{\text{i}n}=1$.

**Fig. 1.**
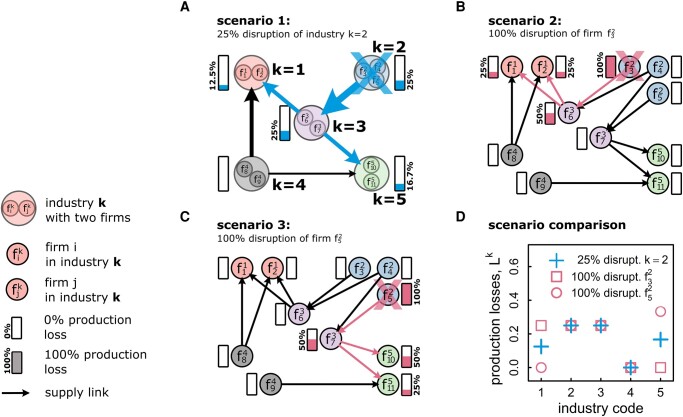
Schematic demonstration of how errors in production loss estimates originate from aggregating a FPN to the IPN. A) Scenario 1, shock propagation on the IPN in response to a *25% initial disruption of industry 2* (blue X) resulting in a 25% production loss (blue bar marks 25% reduction). The disruption of sector 2 can originate from various combinations of shocks to firms $f_{3}^{2},f_{4}^{2}$, and $f_{5}^{2}$. The initial shock spreads downstream to sector 3, leading to a 25% production loss, and further to sector 1 (12.5% loss) and sector 5 (16.7% loss). B) Scenario 2, shock propagation on the FPN in response to a *100% disruption of firm 3* (red X, red bar). The disruption propagates downstream (red edge) to firm 6 (50% production loss), and further to firms 1 and 2 (25% loss). Other nodes are not affected (0% loss, empty bars). C) Scenario 3, shock propagation on the FPN in response to a *100% disruption of firm 5* (red X, red bar), resulting in a 0% production level. The disruption propagates downstream (red edge) to firm 7, (50% loss) and to firms 10 (50% loss) and 11 (25% loss). Other nodes are not affected. D) Comparison of industry-specific production losses, $L^{k}$ (*y*-axis) for industries 1, 2, 3, 4, and 5 (*x*-axis), in response to the aggregated 25% disruption of sector 2 (blue “+”) and the two 100% firm-level shocks, of firms 3 and 5 (red squares and circles). Note that aggregating both firm-level shock scenarios leads to the same 25% shock of industry 2. The production losses of industries 2, 3, and 4 are 0.25, 0.25, and 0, respectively, for all three cascades the symbols “+”, circle, and square overlap. However, the output losses of sectors 1 and 5 are remarkably different for the three different shocks (symbols do *not* overlap). FPN-losses are seen to vary from 0 to 0.25 for sector 1, and from 0 to 0.33 for sector 5, whereas the IPN losses are the same for both firm-level failure scenarios: 0.125 for sector 1, and 0.167 for sector 5. Remarkably, the IPN-loss estimates deviates from the FPN-loss estimates by about 100%.

### How misestimations of output losses result from aggregating firm-level production networks to industries

Figure 1 illustrates how a production shock that propagates on the FPN (*W*) is misestimated by the same shock propagating on the IPN (*Z*), as aggregating the FPN erases crucial intraindustry heterogeneities of firms’ IO relations. To demonstrate the discrepancy, we compare three shock scenarios each corresponding to a 25% disruption of sector 2. Hence, all shocks propagate *identically* on the industry-level network, *Z*, but lead to *distinct* shock propagation cascades on the firm-level network, *W*. Figure 1A shows *scenario 1*, a 25% initial disruption of industry 2 (blue X), at time t=1. The production of sector 2 drops from $s_{k=3}^{\text{o}ut}=4$ units to 3 units, i.e. by 25% (indicated by the blue bar to the right filled by 25%), and the production level, $h_{2}(t)$, is $h_{2}(1)=\phi_{2}=0.75$. This initial shock is specified by the vector of remaining production levels, ϕ=(1,0.75,1,1,1). Then, the shock spreads downstream (blue edge) to sector 3 at t=2 (25% production loss, $h_{3}(2)=0.75$), and at t=3 to sectors 1 (12.5% production loss, $h_{1}(3)=0.875$), and 5 (16.7% production loss, $h_{5}(3)=0.833$).^b^ The shock propagates, as industries 3, 1, and 5 lack inputs for their production processes. Note that the 25% disruption of industry 2 could originate from various combinations of individual shocks to firms, $f_{3}^{2},f_{4}^{2}$, and $f_{5}^{2}$, in industry 2. Figure 1B shows *scenario 2*, a 100% disruption of firm 3, $f_{3}^{2}$, (red X, red bar). The production of firm 3 drops to 0%, i.e. a total operational failure ($h_{3}(1)=\psi_{3}^{1}=0$). The firm-level shock is specified by the remaining production level vector, $\psi^{1}$, where $\psi_{3}^{1}=0$ and $\psi_{i}^{1}=1$, for all i≠3. The disruption propagates downstream (red edge) to $f_{6}^{3}$ (50% production loss, $h_{6}(2)=0.5$), and further to firms 1 and 2 (25% production loss, $h_{1}(3)=h_{2}(3)=0.75$). Aggregating the production losses of firms yields a loss of 25% for industries 1, 2, and 3 and a 0% loss for industries 4 and 5. Figure 1C shows *scenario 3*, the propagation of a 100% disruption of firm 5, $f_{5}^{2}$ (red X, red bar). Aggregating the resulting production losses yields a loss of 25% for industries 2 and 3, a 0% loss for industries 1 and 4, and a 33% loss for industry 5. Figure 1D compares for each industry, *k* (*x*-axis), the industry-specific production loss, $L^{k}$ (*y*-axis), across the three scenarios, 25% shock to sector 2 (blue “+”), 100% shock to firm 3 (red squares), and firm 5 (red circles). When aggregated both firm-level shocks yield the same industry-level shock of 25% disruption of industry 2, and the shock propagation in all three scenarios cause production losses to industries 3 and 4 of 0.25 and 0, respectively—the symbols “+”, circle, and square *overlap*. However, the output losses of sectors 1 and 5 are vastly different across the three shocks—“+”, circle, square do *not overlap*. The FPN-based losses vary from 0 to 0.25 for sector 1 and from 0 to 0.33 for sector 5, whereas the aggregation-based IPN losses are the same for both firm-level shocks, 0.125 for sector 1 and 0.167 for sector 5. The IPN-based loss misestimates the FPN-based losses by 100%. Other network dynamics such as growth, innovation, or productivity spill overs—happening to a large extent at the firm and not the industry level—are potentially affected in similarly drastic ways.

Note that the observed misestimation is not caused by the choice of the production function (which for simplicity is linear in the example), but by the fact that the output vector of sector 3 does not capture the heterogeneity of the output vectors of firms $f_{6}^{3}$ and $f_{7}^{3}$. Firm $f_{6}^{3}$ sells only to firms in sector 1, while $f_{7}^{3}$ sells only to firms in sector 5, i.e. firms $f_{6}^{3}$ and $f_{7}^{3}$ have *no overlap* in their customer industries, see Fig. 1B. Aggregation to the industry level erases this firm-level heterogeneity, as the aggregated industry 3 sells equally to industry 1 and industry 5; see Fig. 1A. Hence, on the FPN same-sized shocks to sector 3 can propagate either to sector 1 or sector 5 (depending on how $f_{6}^{3}$ and $f_{7}^{3}$ are affected), while on the IPN these shocks always propagate identically to sectors 1 and 5. Consequently, the lacking intrasector heterogeneity of aggregated industry-level output vectors causes the misestimation in shock propagation losses. Similarly, IPNs can approximate firm-level shock propagation dynamics only to a limited extent, when firms within the same sector have heterogeneous IO relations. If in the example we assume Leontief production functions instead, the absolute level of production losses would be higher overall, but the discrepancy between the FPN and IPN estimates would remain at 100%.

Note that unlike the literature focusing on aggregate fluctuations of output (16, 17), here we are interested in negative shocks (e.g. as in a crises situation), where there is no possibility for shocks to compensate each other in the aggregate. For example, in the beginning of the COVID-19 pandemic, the supply of most goods and services was reduced simultaneously, while demand increased for only a few sectors (54). Even if firms suffer i.i.d. shocks (that we do not consider here) misestimations due to network aggregation can arise. Firm-level shocks can cancel each other when aggregated to sectors, but in the FPN shocks would still continue to propagate along firms’ supply chains—unless negatively affected suppliers and customers could be replaced instantly. For example, consider *scenario 2* where $f_{3}^{2}$ suffers a 100% disruption, but at the same time firm $f_{5}^{2}$ receives a positive (productivity) shock doubling its production. Then, aggregating the two shocks would cancel at the industry level; industry k=2 would receive a 0% shock. However, at the firm level, the shock propagates as in Fig. 1B, unless the additional output of firm, $f_{5}^{2}$, could be rewired immediately to firm, $f_{6}^{3}$. In that case, the discrepancy between the sector-level and the firm-level production loss estimate would be even larger. In our example, the loss estimates would coincide only when rewiring is instantaneous, but this is unlikely in reality given the empirical evidence of shock propagation in FPNs (29, 45, 46).

We now quantify the relevance of misestimating production losses resulting from the aggregation of real-world FPNs by using a unique dataset that allows us to observe almost every firm-level supply chain relation of the entire production network of Hungary, containing 243,399 firms and 1,104,141 links in 2019, see the Methods section. First, we assess how representative IPNs are of real-world FPNs, by quantifying the intrasector overlaps of firms’ input and output vectors. Second, we quantify the estimation errors of economy-wide and industry-specific production losses that arise when using IPNs to approximate firm-level shock propagation dynamics. Firm-level labor data with monthly time resolution enables us to realistically estimate the size of the COVID-19 shock for *individual firms* in the beginning of 2020. Then, we compare the production losses from propagating a realistic COVID-19 shock and 1,000 synthetic shock realizations, once on the FPN and once on the IPN. We sample the synthetic shocks such that they are of the same size when aggregated to the industry level, but affect firms within industries differently. This feature allows us to clearly show the effects of intrasector heterogeneity in firms’ input–output vectors for estimating production losses, while controlling for size and industry effects.

## Results

### Quantifying input and output vector overlaps of firms

Large overlaps (firms within sectors are similar) would suggest that aggregation to the industry level does not lead to large distortions of network dynamics. Small overlaps (firms within sectors are heterogeneous) would lead to potentially large aggregation effects. First, we aggregate for every firm its firm-level input and output vectors to the industry level (NACE2), see SI Section 1. Second, for each pair of firms, *i*, and, *j*, within a given NACE2 industry, we calculate the input overlap coefficient (IOC) and the output overlap coefficient (OOC) as


(1) $${\text{IOC}}_{ij}=\sum_{k=1}^{m}\min\;\left[{\bar{\Pi }}_{ik}^{\text{i}n},{\bar{\Pi }}_{\;jk}^{\text{i}n}\right],$$

(2) $${\text{OOC}}_{ij}=\sum_{k=1}^{m}\min\;\left[{\bar{\Pi }}_{ik}^{\text{o}ut},{\bar{\Pi }}_{\;jk}^{\text{o}ut}\right],$$

where *m* is the number of NACE2 industries (here 86), and ${\bar{\Pi }}_{i}^{\text{i}n}$. and ${\bar{\Pi }}_{i}^{\text{o}ut}$. are the normalized input and output vectors of firm, *i*, respectively, see SI Section 1. ${\text{IOC}}_{ij}$ specifies the fraction of total inputs, *i* and *j* buy from the same industries. It quantifies the common exposure of *i* and *j* to supply shocks originating from the same upstream industries and indicates the fraction of a demand shock that is forwarded by *i* and *j* to the same upstream industries. ${\text{OOC}}_{ij}$ specifies the fraction of total sales, *i* and *j* sell to the same industries. It quantifies the common exposure of *i* and *j* to demand shocks originating from the same downstream industries and indicates the fraction of a shock that is forwarded by *i* and *j* to the same downstream industries. For more information, see SI Section 2. In Fig. 1B, the relative input vector is ${\bar{\Pi }}_{10}^{\text{i}n}=(0,0,0.5,0.5,0)$ for firm 10 and ${\bar{\Pi }}_{11}^{\text{i}n}=(0,0,1,0,0)$ for firm 11, hence, ${\text{IOC}}_{10,11}=0.5$. The propagation of upstream shocks by 10 and 11 will only overlap by 50% (sector 3), while 50% spread to distinct sectors.

#### Firms within industries are highly different

We show the distribution of the pairwise similarities ${\text{IOC}}_{ij}$ and ${\text{OOC}}_{ij}$ for all firms in NACE2 industry C26, “Manufacture of computer, electronic and optical products” in Fig. 2. Figure 2A–D shows the ${\text{IOC}}_{ij}$ distributions stratified by their number of suppliers (in-degree, $k_{i}^{\mathrm{in}}$). Figure 2A contains all firms that have 1–5 suppliers, Fig. 2B 6–15, Fig. 2C 16–35, and Fig. 2D more than 35. The average similarity of firms’ input vectors is small across all four groups for which the median (vertical solid line) and mean (dashed line) overlaps are 0, 0.121, 0.199 343, and 0.141, 0.196, 0.239, 0.343, respectively. Clearly, the average similarity of input vectors is increasing for firms with more suppliers. The distribution for firms with one to five suppliers (Fig. 2A) is bimodal, most pairs of firms have either almost no overlap or almost perfect overlap. For firms with a few suppliers (Fig. 2B and C) the distributions become unimodal and right skewed, implying that very high similarities appear in the right tail, but are not very frequent. Finally, the distribution of input overlaps for firms with more than 35 suppliers are centered around 0.34 (Fig. 2D). Figure 2E–H shows the distribution of the pairwise ${\text{OOC}}_{ij}$, grouped according to their number of buyers (out-degree, $k_{i}^{\mathrm{out}}$). The bin sizes are the same as before. The average similarity of output vectors is visibly smaller than those of input vectors. The median and mean overlaps for the respective out-degree bins are 0, 0.025, 0.119, 0.119 and 0.054, 0.087, 0.169, 0.143, respectively. The distributions are more concentrated toward low overlaps and remain right skewed for all out-degree bins.

**Fig. 2.**
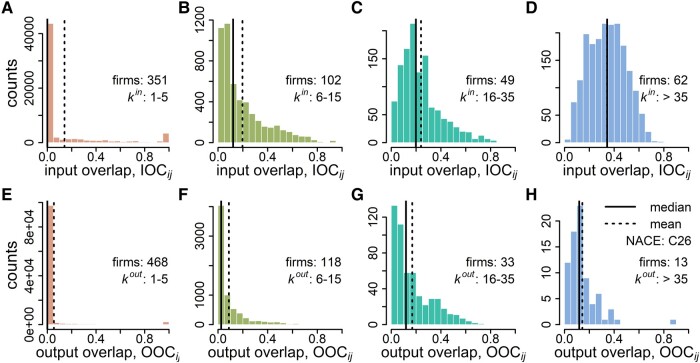
Pairwise similarity distributions of input and output vectors for firms of the NACE class 26, “manufacture of computer, electronic and optical products”. Panels A–D show input vector overlap coefficients, ${\text{IOC}}_{ij}$, and E–H output vector overlap coefficients, ${\text{OOC}}_{ij}$, for four in-degree, $k_{i}^{\mathrm{in}}$ (number of suppliers) and out-degree, $k_{i}^{\mathrm{out}}$ (number of buyers) bins, respectively. Vertical solid lines correspond to median, dashed lines to the average overlap coefficients. A) ${\text{IOC}}_{ij}$ for 351 firms with $1\leq k_{i}^{\mathrm{in}}\leq 5$ suppliers; B) 102 firms with $6\leq k_{i}^{\mathrm{in}}\leq 15$ suppliers; C) 49 firms with $16\leq k_{i}^{\mathrm{in}}\leq 35$ suppliers; D) for 62 firms with more than 35 suppliers. It is clearly visible that the similarity of input vectors is low for all numbers of supplier, but increases on average with the number of suppliers. E) ${\text{OOC}}_{ij}$ distribution for 468 firms with $1\leq k_{i}^{\mathrm{out}}\leq 5$ customers; F) 118 firms with $6\leq k_{i}^{\mathrm{out}}\leq 15$ customers; G) 33 firms with $16\leq k_{i}^{\mathrm{out}}\leq 35$ customers; and H) 13 firms with more than 35 customers. The similarity of output vectors is even lower than for input vectors, and also increases on average with the number of buyers. If industry-level aggregation were fully representative for the IO vectors of firms in NACE C26 in all panels, the distributions would correspond to one single bar at an overlap value of 1.

#### Similarity of firms is low and varies across industries

We now show the summary statistics of the pairwise ${\text{IOC}}_{ij}$ and ${\text{OOC}}_{ij}$ distributions for all NACE2 industries in Fig. 3, in particular, the mean, 5%, 25%, 50% (median) 75%, and 95% percentiles. Only firms with more than 35 suppliers and buyers are included. The *x*-axis shows the 86 NACE2 codes present; the *y*-axis represents the overlap coefficients, each boxplot corresponds to one NACE2 class. Dark blue horizontal bars indicate the median, ($p_{50\%}$), thick dark blue vertical lines indicate the interquartile range ($p_{25\%}$— $p_{75\%}$), thin light blue vertical lines indicate error bars ($p_{5\%}$— $p_{95\%}$), and thin vertical black lines separate NACE1 class affiliations. Empty columns indicate that less than two firms exist in the respective sector and degree bin. Figure 3A shows that the low input overlaps of industry C26 are not just an outlier. The mean of the mean (median) input overlaps, ${\text{IOC}}_{ij}$, across NACE2 industries is 0.35 (0.33) and the SD of mean (median) input overlaps is 0.084 (0.102). This indicates that relatively low input overlaps are the norm with few outliers. The highest median ${\text{IOC}}_{ij}$ are found in the “agricultural industry” (A1–A2), “water collection, treatment and supply” (E36) and in the “transport” sectors (H53), whereas the lowest median ${\text{IOC}}_{ij}$ are found in service sectors, such as “other professional”, “scientific and technical activities” (M74), “travel agency, and related activities” (N79), “sports activities and amusement and recreation activities” (R93), and “activities of membership organizations” (S94). The average SD is 0.156. The SD of SDs is small 0.048, and the length of error bars appears to be relatively homogeneous across sectors, suggesting that the variation of pairwise input overlaps, ${\text{IOC}}_{ij}$, is relatively constant across sectors. Figure 3B shows that output overlaps, ${\text{OOC}}_{ij}$, are on average lower than the input overlaps, but have a higher variation across industries. The mean of the mean (median) output overlaps, ${\text{OOC}}_{ij}$, across all NACE2 industries is 0.282 (0.257) and the SD of mean (median) output overlaps is 0.147 (0.161), indicating that relatively low output overlaps are the norm with several outliers. See SI Section 3 for details.

**Fig. 3.**
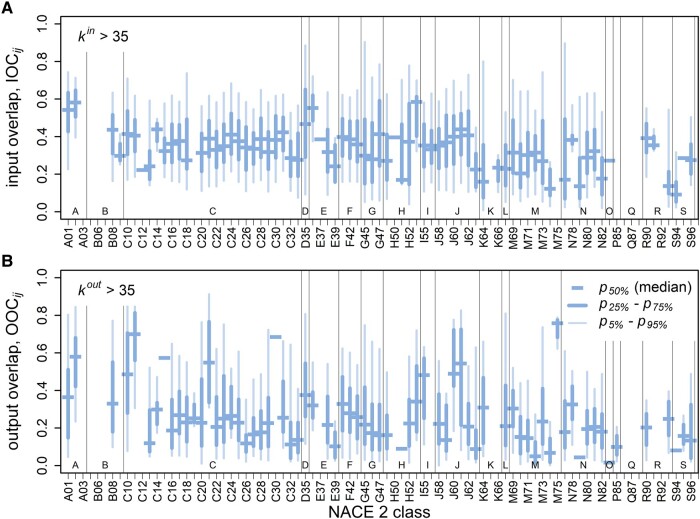
Pairwise similarity distributions of input and output vectors of firms within all NACE2 industries. The overlap coefficient is computed for firms with more than 35 suppliers A) and buyers B), respectively. The dark blue horizontal bars in the boxplots correspond to the median, ($p_{50\%}$), dark blue vertical lines to the interquartile range ($p_{25\%}$– $p_{75\%}$), and thin light blue vertical lines to error bars ($p_{5\%}$– $p_{95\%}$). Thin black vertical lines separate the NACE1 classes. Empty columns indicate sectors with less than two firms in this degree bin. A) Intraindustry input overlap coefficients, ${\text{IOC}}_{ij}$. The average of the mean (median) input overlaps, across all NACE2 industries is 0.35 (0.33) and the SD of mean (median) input overlaps is 0.084 (0.102). The average SD is 0.156. Relatively low input overlaps are the norm with few outliers such as “agricultural industry” (A1–A2), “water collection, treatment and supply” (E36) and “transport” (H53). B) Intraindustry output overlap coefficients, ${\text{OOC}}_{ij}$. The average of the mean (median) output overlaps, across all NACE2 industries is 0.282 (0.257) and the SD of mean (median) output overlaps is 0.147 (0.161). Again we see small overlaps. Output overlaps are on average lower than the input overlaps, but there appears to be more variation across industries. If industry-level aggregation were fully representative for the IO vectors of firms in both panels, all distributions would correspond to a single bar at an overlap value of 1. Not a single industry is even close to that value, the highest similarities are found for sectors such as Veterinary activities (M75), Manufacture of beverages (C11), Manufacture of other transport equipment (C30).

In SI Section 4, we show that for the degree bins 1–5, 6–15, and 16–35, the mean over mean (median) input overlaps are 0.132 (0.009), 0.202 (0.148), 0.269 (0.241), respectively; the respective values for output overlaps are slightly lower. As for industry C26, generally input and output vectors of firms within industries become more homogeneous with the number of suppliers and buyers. In SI Section 5, we show the same analysis for NACE4 industries based on NACE4-level input–output vectors and find that the intrasector variation of input–output vectors is higher than at the NACE2 level. In SI Section 6, we show that our results are robust with respect to the choice of the similarity measure. In SI Section 7, we show that the similarity of input and output vectors of firms *over time* is substantially higher than intraindustry similarities. Individual firms show significant similarity from one year to the next, as expected, while the observed low level of intraindustry similarities capture fundamental heterogeneities.

Overall, we clearly see that input and output overlaps of firms within industries are surprisingly low, across industries and across degree bins. The high level of heterogeneity of input–output vectors of firms within industries shows that for most industries sector-level aggregates are practically not representative for the actual firm-level supply-chain interlinkages and very likely will misrepresent dynamic processes occurring on the firm-level network.

### Misestimating production losses when aggregating networks

We now compare the economy-wide production losses for Hungary caused by a COVID-19 shock propagating once on the FPN, and once on the IPN. Based on firms’ actual employment reductions, the shock realistically captures how *individual firms* were affected by COVID-19 in the beginning of 2020. The shock is represented by the vector, *ζ*, where, $\zeta_{i}$, is the relative reduction of firm *i*’s labor input from January to May 2020, $\zeta_{i}=\max[0,\;1-e_{i}(\text{may})/e_{i}(\text{ jan})]$, and $e_{i}$ is the number of *i*’s employees in the respective month.^c^ The *remaining production capacities* of firms (after the shock) are given by the vector ψ=1−ζ, where, $\psi_{i}\in [0,1]$, is the remaining fraction of firm *i*’s production, e.g. if *i* reduced its employees by 20%, its remaining capacity is $\psi_{i}=0.8$. Aggregating the capacities, $\psi_{i}$, of all firms *i* in sector *k* gives sector *k*’s remaining production capacity, $\phi_{k}$. For details on shock construction and aggregation, see the Methods section.

Following the COVID-19 shock, we simulate how the adaptation of firms’ supply and demand levels propagate *downstream* and *upstream* along the PN, once on the firm level and once on the industry level. We employ the simulation model of Diem et al. (19), where each firm (industry) is equipped with a generalized Leontief production function that distinguishes between essential and nonessential inputs, and considers a heuristic for replacing failed suppliers, see Methods for details. The simulation stops when the production levels of firms have reached a new stationary state at (model internal) time, *T*. Every firm *i* (or sector *k*) has a *final production level*, $h_{i}(T,\psi )\in [0,1]$ ($h_{k}(T,\phi )\in [0,1]$), that depends explicitly on the details of the shock *ψ* (*ϕ*). It represents the fraction of original production, $s_{i}^{\text{o}ut}$, firm *i* (sector *k*) maintains after the shock has propagated. We define the *FPN-based economy-wide production loss* as


(3) $$L_{\mathrm{firm}}(\psi )=\sum_{i=1}^{n}\frac{s_{i}^{\text{o}ut}}{\sum_{\;j=1}^{n}s_{j}^{\text{o}ut}}(1-h_{i}(T,\psi )).$$

It is the fraction of the overall revenue in the network (measured in out-strength, $s_{i}^{\text{o}ut}$, see the Methods section) that is lost due to the shock and the indirect effects of its propagation. The *IPN-based economy-wide production loss*, $L_{\mathrm{ind}.}(\phi )$, is defined accordingly, see Eq. 10 in the Methods section.

#### Misestimating economy-wide production losses

Figure 4 compares the production losses for the two simulations, FPN and IPN. Shock propagation on the FPN leads to a production loss, $L_{\mathrm{firm}}(\psi )$, of 11.5% (red solid line), while propagation on the IPN yields a loss, $L_{\mathrm{ind}.}(\phi )$, of 9.6% (blue dashed line). Aggregated industry-level shock propagation substantially underestimates the production losses caused by firm-level shock propagation dynamics, for the COVID-19 shock, *ψ*, by 16.5%. To quantify the size of misestimations if the firm-level shock was slightly different, we sample 1,000 distinct, synthetic realizations of the COVID-19 shock that are of the same size when aggregated to the industry level, but affect firms within industries differently. First, we take for every sector, *k*, the empirical distribution, $\zeta_{i}$, of firms *i* belonging to that sector. Then, we sample for every company, *i*, of sector *k* a new value, $\zeta_{i}^{\mathrm{sample}}$, from this distribution, replace the old $\zeta_{i}$, and calculate the corresponding remaining production capacity vector, $\psi^{\mathrm{sample}}=1-\zeta^{\mathrm{sample}}$. In this way, we generate the set of 1,000 synthetic capacity vectors, $\Psi =\{\psi^{1},\psi^{2},\ldots ,\psi^{1,000}\}$; for the full algorithm, see SI Section 8. The resulting distribution of FPN-based economy-wide production losses $L_{\mathrm{firm}}(\Psi )$ is shown as histogram and boxplot in Fig. 4. The losses vary strongly from 10.5 to 15.3% of economy-wide production, i.e. losses can vary by a factor of up to 1.46 for different initial shocks of the same size. The actual Hungarian GDP declined by 14.2% in 2020Q2 (55), showing that the losses obtained by our computations are within perfectly realistic bounds; a gross output estimate is not available for comparison.

**Fig. 4.**
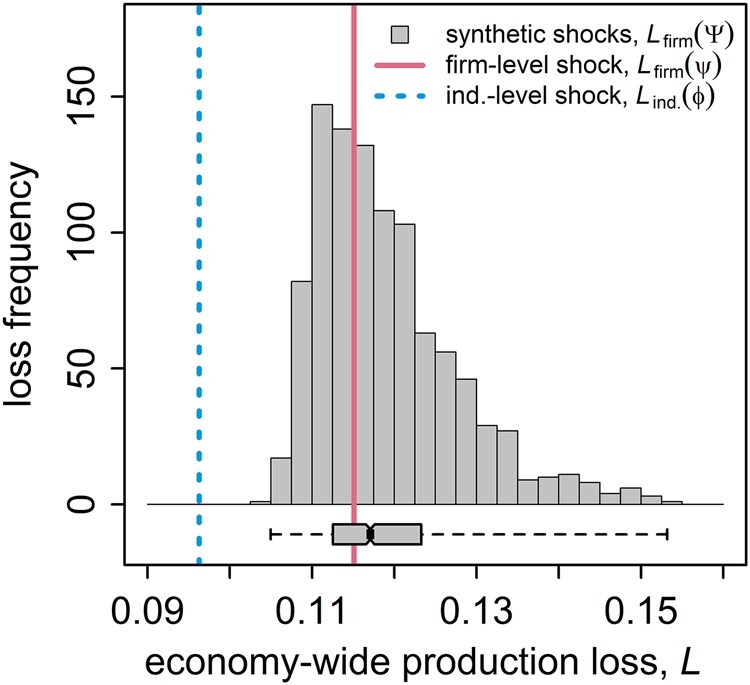
Economy-wide production losses, *L*, obtained from an empirically calibrated and 1,000 synthetic COVID-19 shocks propagating on the aggregated IPN, (blue dashed line) and on the FPN (red line, histogram). The FPN and IPN correspond to the production network of Hungary in 2019; the firm-level shock, *ψ*, correspond to firms reducing their production level proportional to their reduction in employees between January and May 2020, and are taken from monthly firm-level labor data. The NACE2 level shock, *ϕ*, is the aggregation of *ψ*. The 1,000 synthetic shocks, *Ψ*, are sampled such that (when they are aggregated to the NACE2 level) they all have the same size as *ϕ*. The empirically calibrated shock, *ψ*, yields a FPN-based loss, $L_{\mathrm{firm}}(\psi )$, of 11.5% (red line). The synthetic shocks yield a distribution of FPN-based production losses, $L_{\mathrm{firm}}(\Psi )$, ranging from 10.5 to 15.3% of national output (histogram). The median is 11.7% (see boxplot). As a reference, the Hungarian GDP declined by 14.2% in 2020Q2. Note that for the IPN, all realizations, *Ψ*, result in the same production loss, $L_{\mathrm{ind}.}(\phi )$, of 9.6%, by construction. The aggregation to the IPN causes a substantial underestimation of the FPN-based production losses.

Note that the 1,000 synthetic shocks propagating on the IPN always lead to the same economy-wide production loss (9.6%, blue dashed line) because all firm-level shocks, *Ψ*, impact the industry-level production capacities by exactly the same amount, *ϕ*. On average the IPN-based production losses underestimate FPN-based losses by 2.3% of the economy-wide production. In relative terms, losses are on average underestimated by 18.7%. For 10% of the shocks the underestimation is even larger than 26.3% and the maximum underestimation is 37.1%. This tail of large losses is clearly visible in the histogram and is caused by shocks affecting systemically relevant firms stronger (19). The median and mean of the 1,000 losses, $L_{\mathrm{firm}}(\Psi )$, are 11.7 and 11.9%, respectively, and lie close to the FPN-based production loss, $L_{\mathrm{firm}}(\psi )=11.5\%$, based on the original COVID-19 shock, *ψ* (red line).

#### Misestimating industry-specific production losses

We now compare the IPN- and FPN-based production losses for every NACE2 industry separately. We define the *FPN-based industry-specific production loss* of industry, *k*, in response to the COVID-19 shock, *ψ*, as


(4) $$L_{\mathrm{firm}}^{k}(\psi )=\sum_{i=1}^{n}\frac{s_{i}^{\text{o}ut}}{\sum_{\;j=1}^{n}s_{j}^{\text{o}ut}\delta_{\;p_{j},k}}(1-h_{i}(T,\psi ))\delta_{\;p_{i},k}.$$

It is the fraction of revenue (measured in out-strength) that firms in sector *k* lost due to the direct and indirect effects of the shock. The *IPN-based industry-specific production loss*, $L_{\mathrm{ind}}^{k}(\psi )$, is defined accordingly.

In Fig. 5, we show for each NACE2 industry the distribution of *FPN-based production losses*, $L_{\mathrm{firm}}^{k}(\Psi )$, caused by the 1,000 synthetic shocks as boxplot. The *IPN-based production losses*, $L_{\mathrm{ind}.}^{k}(\phi )$ are indicated by the blue “+”es, the *FPN-based production losses* for the original COVID-19 shock, $L_{\mathrm{firm}}^{k}(\psi )$, are given by red “x”es. It is clearly visible that for many industries losses vary strongly across the identically sized shocks, but also the variation between industries is noteworthy. For all but two industries (M73, N82), the production loss distributions are right skewed, few industries (B06, C15, K65, M75, Q87, and R92) have substantial outliers (gray dots) above 3 times the interquartile range. This means that for some particular shock realizations, these sectors can suffer extremely large losses. The minimum and maximum values of production losses for different initial shocks can differ by factors of up to 9.5 (B06), 6.0 (B07), 5.7 (C12), 6.2 (J61), 41 (K65), or 25.9 (Q87). The median (mean) ratios of maximum to minimum loss is 2 (3.2). This variation in production losses across different shocks is inaccessible when using aggregated IPN data; it cannot be inferred from the blue “+”es. The large variations emerge as different shocks affect firms at different positions in the supply networks that have different systemic relevance (19). IPN-based losses (“+”es), lie frequently below the lowest FPN-based loss, while FPN-based COVID-19 losses (“x”es) lie within boxplots. The industries where IPN-based shock propagation underestimates output losses most are C26 (−59.5%), C28 (−53.5%), J58 (−51.3%), C25 (−50.3%), J63 (−47.8%), and C20 (−42.1%). Overestimation of production losses from using IPN-based losses are highest for sectors, K66 (150%), C19 (87.4%), R91 (83.3%), Q88 (80%), S94 (65.4%), and E39 (42%). For other sectors, see SI Section 9. We calculate for each industry the *mean absolute deviation* and take the average across industries, yielding 30.2%.

**Fig. 5.**
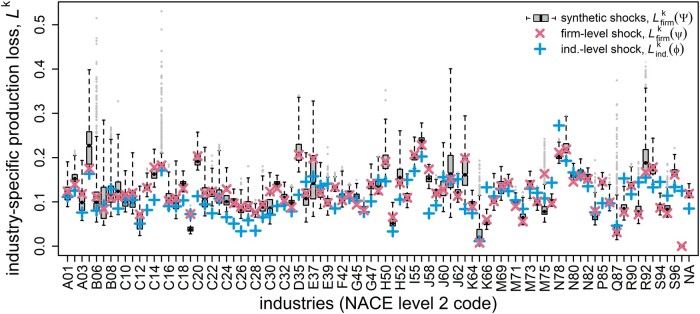
Comparison of industry-specific production losses, $L^{k}$, obtained from an empirically calibrated and 1,000 synthetic COVID-19 shocks propagating on the aggregated IPN (blue “+”es) and on the FPN (red “x”es, boxplots). For most industries, the FPN-based production losses, $L_{\mathrm{firm}}^{k}(\Psi )$ (boxplots) vary strongly across the 1,000 synthetic shocks even though shocks have the same size when aggregated to the industry level. Shock propagation on the industry level (blue “+”es) cannot capture this variation. IPN-based production losses typically underestimate the FPN-based production losses severely.

#### Varying the production function

Even though there is empirical evidence that firms struggle to substitute inputs after suppliers are hit by large natural disasters (29, 45, 46), the Leontief production function has been criticized as being too rigid with respect to input substitution, and, hence could be the cause for the large discrepancies between FPN- and IPN-based loss estimates. Hence, we consider the hypothetical case that shocks propagate on the same PN, but assuming that all firms have linear production functions, allowing for full substitution across inputs, see SI Section 10. We find that the distribution of economy-wide production losses, $L_{\mathrm{firm}}(\Psi )$, ranges from 9.5 to 10.8%, and that these losses are again substantially underestimated by IPN-based loss estimates of 5.5%. This is substantially less variation than when more realistic nonlinear generalized Leontief production functions are used. As expected, we find that the linear production function assumption makes the economy-wide production losses less dependent on which exact firms within industries are impacted by shocks. However, the variations of industry-specific production losses, $L_{\mathrm{firm}}^{k}(\Psi )$, are still very large for many sectors. This emphasizes immediately that in order to correctly estimate sector-level production losses, it is crucial which firms are affected by shocks, even in the best-of-all worlds, where shocks would propagate linearly (i.e. inputs can be fully substituted).

#### Misestimating production losses in general equilibrium

We next show that aggregating production networks also causes large discrepancies between FPN- and IPN-based production loss estimates in a *general equilibrium* (GE) framework. We use the standard production network model of Acemoglu et al. (16), where a household consumes the inputs of firms (sectors) according to a Cobb–Douglas utility function and each firm (sector) is equipped with a Cobb–Douglas production function that takes labor and the outputs of other firms (sectors) as inputs, see Methods. Assuming utility and profit maximization of households and firms, respectively, while taking prices and wages as given, the model has a closed-form solution for the economy-wide output loss in response to a (exogenous) shock-affecting firms (sectors). Based on this result, we define the *FPN-based aggregate output loss in general equilibrium* as


(5) $$L_{\mathrm{firm}}^{\text{G}E}(\psi )=\sum_{i=1}^{n}v_{i}(1-\psi_{i}),$$

where *v* is called *influence vector*, a network centrality measure that is closely related to *PageRank*, see Methods. $L_{\mathrm{firm}}^{\text{G}E}(\psi )$ is the percentage loss in GDP of the overall network due to quantity and price adjustments caused by the shock and its propagation. The *IPN-based economy-wide production loss in GE*, $L_{\mathrm{ind}.}^{\text{G}E}(\phi )$ , is defined analogously.

Figure 6 shows that the empirically calibrated shock, *ψ*, yields a FPN-based loss, $L_{\mathrm{firm}}^{\text{G}E}(\psi )$, of 7.1% (red line). The synthetic shocks yield a distribution of FPN-based production losses, $L_{\mathrm{firm}}^{\text{G}E}(\Psi )$, ranging from 5.8% to 8.2% of national output (histogram). The median is 7.2% (see boxplot). Note that for the IPN all realizations, *Ψ*, result in the same production loss, $L_{\mathrm{ind}.}^{\text{G}E}(\phi )$, of 5.5%, by construction. On average, the IPN-based production losses underestimate FPN-based losses by 1.7% of the economy-wide output. In relative terms, losses are on average underestimated by 23.7%. For 10% of the shocks, the underestimation is even larger than 28.4% and the maximum underestimation is 33.1%. Notably the distribution, $L_{\mathrm{firm}}^{\text{G}E}(\Psi )$, is left skewed, i.e. large losses occur less frequently in comparison to, $L_{\mathrm{firm}}(\Psi )$, that is computed with the framework of Diem et al. (19). The percentage values are not directly comparable as, $L_{\mathrm{firm}}^{\text{G}E}(\Psi )$ measures change in GDP, whereas $L_{\mathrm{firm}}(\Psi )$ measures change in aggregate firm out-strength.

**Fig. 6.**
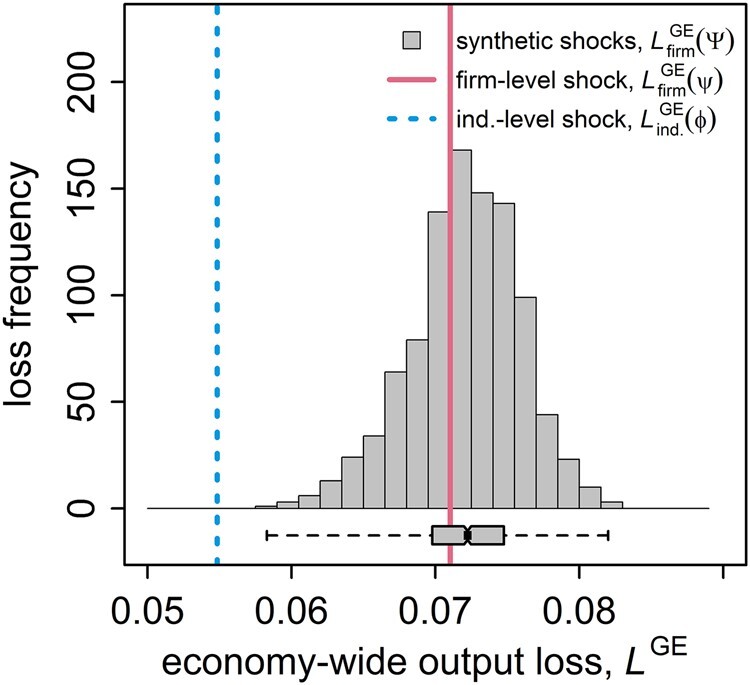
General equilibrium economy-wide output losses, $L^{\text{G}E}$, obtained from an empirically calibrated and 1,000 synthetic COVID-19 shocks propagating on the aggregated IPN (blue dashed line) and on the FPN (red line, histogram). We apply the same firm level, *ψ*, sector level, *ϕ*, and 1,000 synthetic shocks, *Ψ*, as previously. The empirically calibrated shock, *ψ*, yields a FPN-based loss, $L_{\mathrm{firm}}^{\text{G}E}(\psi )$, of 7.1% (red line). The synthetic shocks yield a distribution of FPN-based production losses, $L_{\mathrm{firm}}^{\text{G}E}(\Psi )$, ranging from 5.8 to 8.2% of national output (histogram). The median is 7.2% (see boxplot). Note that for the IPN all realizations, *Ψ*, result in the same production loss, $L_{\mathrm{ind}.}(\phi )$, of 5.5%, by construction. Also in the general equilibrium setting, the IPN-based losses substantially underestimate the FPN-based production.

## Discussion

Production networks are fundamental for explaining and predicting dynamical economic phenomena. For almost a century, these were only accessible as aggregated IPNs, usually represented as IOTs. Only recently, large-scale FPNs, covering entire economies have become available. Based on a unique FPN dataset, containing almost all buyer–supplier links of the Hungarian economy, we demonstrated on the one hand that the aggregation of production networks to the industry level cannot be expected to yield anything close to correct predictions of dynamical processes, such as the propagation of short-term shocks through production networks. On the other hand, we showed that using firm-level supply network data instead, a much more realistic picture can emerge.

We first showed that industries are insufficient to describe the firms they contain, because firms within industries are highly heterogeneous with respect to the industries they buy from and sell to. Specifically, two firms within the same industry spend on average only 23.5% on inputs from the same industry, and sell on average only 19.3% of their revenues to the same industry. Even when two firms belong to the same industry, their industry-level input and output vectors will differ substantially. Therefore, IPN data provides an incomplete description of the actual structure of an economy’s production network and likely causes substantial misestimations of dynamic processes occurring on the firm level.

We next demonstrated that the aggregation of FPNs causes indeed large misestimations for shock propagation dynamics and the resulting production losses. The demonstration is based on a COVID-19 shock that is realistically calibrated with firm-level employment data and 1,000 synthetic COVID-19 shocks of the same size. While economy-wide production losses, in response to the 1,000 shock scenarios, simulated on the FPN range from 10.5 to 15.3% (mean 11.9%), the corresponding IPN-based production losses are 9.6%. In the worst case scenario, the underestimation amounts to 37.1%. For single industries, the largest average misestimation of production losses range from −59.5 to 150%. Our results are robust under the extreme assumption that all firms have a linear production function (i.e. firms can fully substitute different inputs for each other) and they do not depend on the specific shock propagation model of Diem et al. (19); aggregated IPNs also lead to a large underestimation of losses in standard Cobb–Douglas general equilibrium production network models. Our estimates show a drastically reduced ability of predicting the response of an economy to shocks (in particular how shocks propagate) when relying on *aggregated* IPN instead of FPN data.

### Generalizability of results

Our results are derived from the FPN of a particular country (Hungary), but we expect them to be generalizable to FPNs of other countries. A recent comparison of FPNs (including Hungary) shows that the surveyed networks share a number of topological features, such as power-law degree, strenght, and influence vector distributions (23). Since these features are key drivers for the nature of shock propagation, generalizability should be given. Note that the commonality in influence vector distributions provides strong evidence that misestimations due to aggregation are likely in other countries. Most likely, similar discrepancies between network dynamics calculated on disaggregated agent-level networks and their aggregated counterparts occur also in other types of (economic) networks. For example, in financial exposure networks, the detailed network topology is crucial for how financial shocks propagate in response to a bank’s failure (51), hence, we expect that aggregating financial networks would lead to similarly sized misestimations in financial losses and systemic risks. Another example is climate stress-testing, where the aggregation of individual assets (establishments) to single firms can cause an underestimation of financial losses from physical risks (e.g. hurricanes) that affect firms’ assets heterogeneously (56). Misestimations of network dynamics due to aggregation are also likely in noneconomic settings such as epidemic spreading. Topological features of social contact networks are crucial for the dynamics of epidemic spreading (57). Also here aggregating networks (e.g. in so-called mean-field models, like SIR) can lead to well-known misestimations of important metrics such as the epidemic threshold (58).

Our results show a consistent underestimation of production losses when shock propagation is assessed with aggregated data. An underestimation of losses was also found, e.g. when applying a dynamic input output (DIO) model to simulated production networks with 500 firms and an aggregated network with 15 sectors (53), or when applying homogeneous sector-level instead of heterogeneous firm-level shocks to the same DIO model with a transportation network extension (14). Yet, it is a priori not clear under which conditions the aggregation of production networks leads to an underestimation of losses. To establish such conditions for simulation-based models, e.g. Refs. (14, 19, 28, 53), a systematic analysis of which network and which model features lead to what misestimation is required. This might have to be carried out with systematic simulation studies. Features that are likely to matter range from network topology measures (e.g. degree, strength, link weight distributions) to various firm-level heterogeneities (e.g. of IO vectors of firms within sectors), features of production functions (e.g. input substitution), and behavioral model assumptions (e.g. ease and speed of replacing failed suppliers and customers). For economic models based on the Leontief inverse (e.g. through output multipliers or the influence vector)—and thus do not require a simulation of shock propagation dynamics—analytical results are available. These suggest that aggregation leads to misestimation when sectors have heterogeneous IO vectors (42). More generally, for a wide range of networks—that have a large spectral gap and are not sparse—a number of network centrality measures (including the influence vector) can be well approximated based on the networks’ strength distribution (59). Under these conditions, the aggregation of a network should cause only small estimation errors in production losses when these losses are based on, e.g. output multipliers or the influence vector. For these reasons, we can expect misestimations from aggregating FPNs as the heterogenity across firms’ IO vectors is large and networks are sparse. However, neither the analytical result on sparsity nor those on heterogeneity specify conditions for when aggregation causes an *under*estimation of losses.

### Implications for economic modeling and policy making

The presented results imply a range of immediate consequences for economic modeling, in particular for short-term economic dynamics such as shock propagation, but also more generally for the reliability of industry-level IO models in the context of testing policy implications.

First, our findings make it clear that the size of losses from shock propagation depends crucially on which exact firms are affected by the initial shock. Crises such as COVID-19, the war in Ukraine, or large natural disasters can affect firms within the same industry sectors and regions very differently and, hence, the exact materialization of the shock can lead to significantly different indirect economic losses. Aggregated industry-level models, such as IO models, can by design not account for this, potentially underestimating tail losses that appear when a group of systemically important firms receive shocks at the same time. Modeling shock propagation on FPNs might significantly improve economic assessments of crises of this kind.

Second, our results raise the question if and how microlevel (total factor productivity) shocks can be aggregated in real-world empirical production network structures. In Ref. (16), the effect of a sector-level shock on the aggregate economy is calculated as the weighted sum of the sectors’ shocks and the sectors’ influence vector elements. Analogously, the aggregate effect of a firm-level shock is calculated by summing the firms’ shocks weighted by their influence vector elements. In equilibrium, the elements of the influence vector correspond to the sales shares (revenue of the sector [firm] divided by total revenue of the economy) of sectors and firms, respectively. Hence, the aggregate shock to the economy should be the same when derived from aggregating firm-level and sector-level shocks. However, our results suggest a substantial deviation from this result, adding to the results showing that Hulten’s theorem is not a good approximation when shocks are large (60). Our results corroborate earlier theoretical models that emphasize local nonlinear interactions of individual production units matter for microlevel shocks to cause system-wide variation in production (61).

Third, our method for creating an ensemble of synthetic shock scenarios that are identical on the industry level, but affect firms differently can be used to estimate realistic confidence intervals (CIs) for economic impacts of crises. Experts can define a shock on the industry level (as done routinely for IO models) and obtain distributions of the quantity of interest for each firm, specific sectors or the whole production network. This approach could reveal which combination of shocks to individual firms causes particularly dangerous scenarios that would go unnoticed with industry-level models. This is useful for designing scenarios in economic stability stress tests.

Fourth, the presented framework extends well beyond shock propagation. Other forms of network dynamics that are certainly distorted by industry-level aggregation include economic growth (4), the estimation of CO_2_ emissions of economic activity (43, 44, 62), or the spread of price increases. Detailed future research on these topics, considering the details of firm-level production networks is necessary. These topics happen on larger time-scales and will be overlaid with other dynamics that were not covered here. These dynamics are most likely more complicated than the ones of short-term shock propagation and therefore it is reasonable to assume that the effects of aggregation are even stronger in these situations.

Fifth, specifically, for estimating CO_2_ emissions of industry sectors and countries, aggregating IOTs causes substantial errors in emission estimates (43, 44, 62). Our results indicate, firms in the same NACE industries use very different inputs and sell to very different industries and therefore their resulting scope-3 emissions (indirect CO_2_ emissions along supply chains) can differ substantially. Firm-level data will be crucial for reliable and targeted CO_2_ emission estimates and for designing green transition enhancing economic policies that can target problematic firms (63).

Sixth, in the past economic models, e.g. for assessing economic effects of natural disasters, Henriet et al. (53) have worked with the simplifying assumption that firms within an industry are the same with respect to their input and output vectors. Now with new firm-level data being available, we can actually empirically assess the validity of this assumption and find that firm-level heterogeneity is actually large. Our results and the above discussion suggest that for estimating and predicting effects of natural disasters more reliably, production network models should feature firm-level heterogeneity within industries.

### Limitations and future research

There is a list of limitations of the presented material. For self-consistency, the industry-level production network used here is simply the aggregation of a FPN. IOTs are constructed with extensive survey methodologies and the available tables can differ (22). However, also IOTs are aggregations of underlying firm- and establishment-level networks and are likely to be affected by the same problems and to a comparable extent. Secondary NACE categories of firms are not contained in our dataset. Larger firms producing several different types of products (in potentially several establishments) are fully aggregated to their primary NACE category. This could lead to an overestimation of heterogeneity of input and output vectors within industries. Future research should quantify the heterogeneity of input and output vectors of establishments used for creating IOTs. A potentially strong limitation is that we do not have information of firms’ international import and export links. Consider two firms in one sector, one imports a specific input and the other sources it domestically we would overestimate the heterogeneity of their input vectors. However, for the Belgium production network, it has been shown that only a small fraction of firms have direct import and export linkages (34). In practice, high-quality economic data to calibrate industry-level economic models is widely available and some have achieved good forecasting performance, e.g. Ref. (15). To calibrate firm-level models, substantially larger amounts of data are needed. For example, quantifying how a shock (e.g. a natural disaster) affects hundreds of thousands of firms is substantially harder than for a few dozens of sectors. Firms within sectors do react differently, modeling their behavior realistically, involves many assumptions, but up to now data for calibration is scarce. We demonstrated that for how shocks propagate details do matter. In our simulation model important nonlinearities appear in the generalized Leontief production functions (GLPFs) of companies. The calibration of firms’ GLPFs is currently a rough approximation combining firms’ NACE4 industry affiliation with an expert based survey for 56 industry sectors conducted in Ref. (15). The calibration of the GLPF needs refinement in the future, e.g. with large-scale firm-level surveys. Note that this does not affect our results obtained with linear production functions and the GE model.

Our results point out relevant open questions. Duprez and Magerman (36) find large idiosyncrasies in price changes of producers within the same product categories. It would be interesting to see, whether these could be explained by the heterogeneity of firms’ input and output vectors. In the direction of IOTs, differences of Leontief multipliers for different aggregation levels of IOTs with potential implications for predicting economic growth were reported (4). It would be of interest to see how this extends across all scales to the firm level. Heinrich et al. (64) show that correlation structures found on the sector level can vanish when economic analysis is taken to the firm level. Also for this phenomenon the intrasector heterogeneity of firms could be part of the explanation. The effects of heterogeneities should also be checked for establishment-level supply networks that are still rarely available at larger scale (65). Since our data shows that input and output vectors of firms remain relatively stable from one year to another. This raises the question of how fast can production networks adapt to technological change? And would an aggregate perspective of production networks underestimate or overestimate the speed of adaption in the network? Further, the large heterogeneity of inputs and outputs of firms within the same sectors implies that the same output can be produced from different input combinations. If one input is no longer available, this might affect a certain company, while others continue production. In the longer term, if one input is becoming structurally more expensive, firms could change the production to mimic competitors that use a different input mix to produce the same good. This raises the question if this large amount of heterogeneity in input and output vectors is actually a source of resilience in the production network, or just an inefficiency in knowledge transfer?

## Conclusion

In this work, we showed the importance of modeling production networks at the firm level. However, currently data on FPNs exist only in very few countries and is rarely available to research. This work shows how necessary it is to make these data usable for researchers and policy institutions. Complementing traditional industry-level models with new models that are specifically designed for firm-level data is a great opportunity forward for both reliable policy making and progress of scientific research on resilience and transformability of the current economy.

## Methods

### Data

The Hungarian FPN, *W*, is based on the 2019 value added tax (VAT) microdata of the Hungarian Central Bank (19, 22). Supply links between two firms are present if the tax content of the transactions was above 1 million Forint for 2018Q1–Q2 and 100,000 Forint for 2018Q3–2019Q4 (approx. 250 euros). The link weight, $W_{ij}$, represents the monetary value of all transactions between the two firms in the given year. We filter the data for stable supply links and keep a link if at least two supply transactions occurred in two different quarters, i.e. we exclude one-off transactions. The filtering reduces the number of links from approx. 2 millions to 1.1 millions, but the transaction volume drops only by approx. 10%. The number of firms drops from 315,259 to 243,339 in 2019 and from 296,992 to 185,322 in 2018; for summary statistics, see SI Section 11. Imports and exports are not contained in the dataset. The industry affiliation of firms, $p_{i}$, correspond to the NACE classifications contained in the Hungarian corporate tax registry. On the NACE2 level, 86 different classes are present, and on the NACE4 level, 587 different classes are present. In 2019, the NACE affiliation is missing for 62,782 firms; in 2018 for 42,385 firms. We treat them as a residual NACE class.

### Constructing firm- and industry-level COVID-19 shocks

The employment data (collected by the Hungarian tax authority available at the central bank) contain the number of employees, $e_{i}(\tau )$, firm *i* employed in the respective month *τ*. We assume that labor is an essential (Leontief style) input to a firm’s production, Eq. 7, and that after a shock, firms only keep the amount of employees needed to operate at the new reduced production level. Therefore, we treat the empirical reduction of employees as a signal for how strong the firm was affected by the consequences of the pandemic in beginning of 2020. No furlough schemes were in place in Hungary. Note that January is sufficiently distant from COVID-19 affecting Europe, and May is the time when the initial shock should be fully incorporated in the employment data, as there is a 2-month leave notice period in Hungary. Note that, hence, the initial shock corresponds to the effects occurring in the first quarter in 2020 even though May data are used. The Hungarian labor data are available for approx. 160,000 firms. For the firms with no data, we impute the value by drawing the fraction of employment from firms in the respective NACE4 category where the data are available. We conduct the imputation 1,000 times and receive 1,000 completed vectors. For each of them, we calculate the value of economy-wide lost production, $L_{\mathrm{firm}}$, see Eq. 3. We choose the completed shock vector that yields the median loss of production as *ψ*. The corresponding industry-level COVID-19 shock is calculated by aggregating the vector *ψ*, to the NACE2 industry level. As firms within a sector mostly have different ratios of in-strength and out-strength, i.e. $s_{i}^{in}/s_{i}^{out}\neq s_{j}^{in}/s_{j}^{out}$, we aggregate the firm-level production capacities to a vector of downstream constrained, $\phi^{\text{d}}$, and a upstream constrained remaining production capacity, $\phi^{\text{u}}$. For industry *k*, $\phi_{k}^{u},\phi_{k}^{d}$ are calculated as


(6) $$\phi_{k}^{\text{u}}=\frac{\sum_{i=1}^{n}\psi_{i}\;s_{i}^{\text{i}n}\;\delta_{\;p_{i},k}}{\sum_{i=1}^{n}s_{i}^{\text{i}n}\;\delta_{\;p_{i},k}},\;\phi_{k}^{\text{d}}=\frac{\sum_{i=1}^{n}\psi_{i}\;s_{i}^{\text{o}ut}\;\delta_{\;p_{i},k}}{\sum_{i=1}^{n}s_{i}^{\text{o}ut}\;\delta_{\;p_{i},k}}.$$

We use the notation $\phi =(\phi^{\text{u}},\phi^{\text{d}})$. We show the aggregated shock, *ϕ*, for each NACE2 class, see SI Section 9. Creating synthetic shocks, $\psi^{1},\psi^{2},\ldots ,\psi^{1,000}$,—that when aggregated to the industry level are identical to $\phi^{\text{d}}$ and $\phi^{\text{u}}$ —can be achieved by $\psi^{1},\psi^{2},\ldots ,\psi^{1,000}$ fulfilling Eq. 6. This implies that the aggregated firm-level shocks all fulfill $\phi^{\text{u},1}\;=\;\phi^{\text{u},2}\;=\;\cdots \;=\;\phi^{\text{u},1000}$ and $\phi^{\text{d},1}\;=\;\phi^{\text{d},2}\;=\;\cdots \;=\;\phi^{\text{d},1000}$. For details, see SI Section 8.

### Shock propagation model

The production process of each firm *i* is represented by a generalized Leontief production function (GLPF), defined as


(7) $$x_{i}=\min\left[\min_{k\in I_{i}^{es}}\left[\frac{1}{\alpha_{ik}}\Pi_{ik}\right],\beta_{i}+\frac{1}{\alpha_{i}}\sum_{k\in I_{i}^{ne}}\Pi_{ik},\frac{1}{\alpha_{l_{i}}}l_{i},\frac{1}{\alpha_{c_{i}}}c_{i}\right].$$

$\Pi_{ik}$ is the amount of input *k* firm *i* uses for production, $I_{i}^{\text{e}s}$ is the set of essential inputs, $I_{i}^{\text{n}e}$ is the set of nonessential inputs of firm *i*; $l_{i}$ and $c_{i}$ are *i*’s labor and capital inputs. The essential and nonessential input types of firms are assigned according to their industry affiliation (NACE4) and an expert-based survey for 56 industry sectors conducted by Pichler et al. (15). The parameters $\alpha_{ik}$ are technologically determined coefficients, $\beta_{i}$ is the maximum production level possible without nonessential inputs $k\in I_{i}^{\text{n}e}$, and $\alpha_{i}$ is chosen to interpolate between the full production level (with all inputs) and $\beta_{i}$. All parameters are determined by *W*, $I_{i}^{\text{e}s}$, and $I_{i}^{\text{n}e}$. The COVID-19 shock, *ψ*, propagates through the Hungarian production network in the following way. Initially, at time t=0, the network, *W*, is stable and the production amount of each firm *i* corresponds to its out-strength, $x_{i}(0)=s_{i}^{\text{o}ut}$, where $s_{i}^{\text{o}ut}$ corresponds to firm *i*’s original revenue from its activity in the FPN, *W*. We denote firm *i*’s remaining fraction of production, at time *t* as $h_{i}(t)=x_{i}(t)/x_{i}(0)$, hence at time t=0 before any shocks occur $h_{i}(0)=1\;\forall \;i$. At time t=1, the initial shock materializes and production levels of each firm *i* drop to the remaining production capacity, $h_{i}(1)=\psi_{i}$. Then, we simulate how firms propagate the received shock upstream by reducing their demand to suppliers and downstream by reducing their supply to customers. Missing nonessential inputs cause production reductions in a linear fashion, while a lack of essential inputs affects output in the nonlinear Leontief way, i.e. downstream shocks can have strong negative impacts on production, depending on the supplier–buyer industry pair. The loss of a customer leads to a production reduction proportional to the customers’ revenue-share, i.e. upstream shocks have only linear impacts. For each firm, *i*, we update the production output, $x_{i}^{\text{d}}(t+1)$, at t+1, given the downstream constrained production levels of its suppliers, $h_{j}^{\text{d}}(t)$, at time *t* as


(8) $$\begin{array}{rl} x_{i}^{d}(t+1) & =\min[\min_{k\in I_{i}^{es}}\left(\frac{1}{\alpha_{ik}}\sum_{\;j=1}^{n}W_{\;ji}h_{j}^{\text{d}}(t)\delta_{\;p_{j},k}\right),\\ & \;\beta_{i}+\frac{1}{\alpha_{i}}\sum_{k\in I_{i}^{ne}}\sum_{\;j=1}^{n}W_{\;ji}h_{j}^{\text{d}}(t)\delta_{\;p_{j},k},\psi_{i}x_{i}(0)]. \end{array}$$

The production output, $x_{i}^{\text{u}}(t+1)$, of firm *i* at t+1, given the upstream constrained production level of its customers, $h_{l}^{\text{u}}(t)$, at time *t* is computed as


(9) $$x_{i}^{u}(t+1)=\min\left[\sum_{l=1}^{n}W_{il}h_{l}^{\text{u}}(t),\psi_{i}x_{i}(0)\right].$$

The algorithm converges at time *T*, yielding final production levels, $h_{i}(T,\psi )$, for each firm *i*. The dependence of the final production level on the initial shock is made explicit by writing $h_{i}$ as a function of *ψ*. Note that the quantity, $s_{i}^{\text{o}ut}(1-h_{i}(T,\psi ))$, is the amount of lost revenue of firm *i* due to the initial shock and its propagation. For a complete description of the algorithm, see Ref. (19). For simulating shocks on the industry-level network, *Z*, in Eqs. 8 and 9, we replace *W* with *Z*, in Eq. 8, $\psi_{i}$ with $\phi_{i}^{\text{d}}$, and in Eq. 9, $\psi_{i}$ with $\phi_{i}^{\text{u}}$. This results in the final production levels, $h_{k}(T,\phi )$, for each sector, *k*, and we set $L_{\mathrm{ind}.}(\phi )=1-h_{k}(T,\phi )$. The overall production loss, $L_{\mathrm{ind}.}^{k}(\phi )$, is calculated analogously as in Eq. 3, based on the out-strengths, $s_{k}^{\text{o}ut}$, of sectors, *k*, as


(10) $$L_{\mathrm{ind}}(\phi )=\sum_{k=1}^{m}\frac{s_{k}^{out}}{\sum_{l=1}^{m}s_{l}^{out}}(1-h_{k}(T,\phi )).$$

### Shock propagation in GE setting

The GE model in Ref. (16) uses the household utility function $U(c)=q\prod_{i=1}^{n}c_{i}^{1/n}$, where $c_{i}$ is the consumption of output *i*. Firm *i*’s production output is given by $x_{i}=z_{i}^{\alpha }l_{i}^{\alpha }\prod_{\;j=1}^{n}x_{\;ji}^{(1-\alpha )A_{\;ji}}$, where $z_{i}$ is the productivity level (or productivity shock), $l_{i}$ is the amount of labor used for production, $x_{\;ji}$ is the amount of output of firm *j* used in firm *i*’s production, *α* is the labor share of production, and $A_{\;ji}=W_{\;ji}/s_{i}^{\text{i}n}$ is the fraction of value of the input provided by firm *j* out of the value of all inputs used by firm *i*, $s_{i}^{\text{i}n}$. In GE, the log GDP of this FPN is given by $\log\;({\text{GDP}}_{\text{f}irm})=\log\;(y_{\text{f}irm})=v^{⊤}\log\;(z)$, with $v=\frac{\alpha }{n}(I-(1-\alpha )A{)}^{-1}1$, where 1 is a vector of ones, *I* is the identity matrix, and *A* is the input share matrix with $A_{ij}=W_{ij}/s_{j}^{\text{i}n}$ (its columns sum to 1). We use the common assumption of α=0.5, see Ref. (23). The extent of shock propagation and, hence, the reduction in aggregate output in response to a shock are determined by the production network’s influence vector. The influence vector, *v*, is a network centrality measure, whose elements, $v_{i}$, give the importance of firm *i* for the propagation of shocks in the production network, *W*. It is closely related to the Leontief inverse in the standard IO model and coincides with the *PageRank* of $W^{⊤}$. We compute *v* with the R igraph PageRank implementation (66). Note that the transpose occurs since in PageRank the matrix, *W*, is out-strength (row) normalized, and here *A* is the in-strength (column) normalized matrix, *W*. Note that in Ref. (16), adjacency matrices are defined in the transposed way.

In the GE setting, one can calculate the percent reduction in output in response to the employed empirical and the 1,000 synthetic COVID-19 shocks, ψ,Ψ, in the following way. The productivity vector *z* gives us the aggregate log-output, $\log\;(y_{\text{f}irm})$, and for a new productivity vector, $\tilde{z}$, we receive a new level of log-output, $\log\;({\tilde{y}}_{\text{f}irm})=v^{⊤}\log\;(\tilde{z})$. Note that the influence vector, *v*, is independent of the shock *z*, and, hence, does not change when the shock changes (in this model). By subtracting the old equilibrium state from the new, we calculate the percent reduction in output as $\log\;({\tilde{y}}_{\text{f}irm}/y_{\text{f}irm})=v^{⊤}\log\;(\tilde{z}/z)$, where $\log\;({\tilde{z}}_{i}/z_{i})$ corresponds to the percentage change in productivity, for firm *i*; see also Ref. (67) which uses this approach. By setting $\log\;(z_{i}/{\tilde{z}}_{i})$ to $\zeta_{i}=(1-\psi_{i})$, we receive Eq. 5, as


(11) $$L_{\mathrm{firm}}^{\text{G}E}(\psi )=\log\;\left({\tilde{y}}_{\text{f}irm}/y_{\text{f}irm}\right)=v^{⊤}(1-\psi ).$$

The change in output computed from the aggregated IPN, *Z*, is calculated accordingly as $L_{\mathrm{ind}.}^{\text{G}E}(\phi )={\tilde{v}}^{⊤}(1-\phi^{\text{d}})$, where $\tilde{v}=\frac{\alpha }{n}(I-(1-\alpha )\tilde{A}{)}^{-1}1$ and $\tilde{A}$ is the sector-level input share matrix with element, ${\tilde{A}}_{kl}=Z_{kl}/s_{l}^{\text{i}n}$. We use only the out-strength weighted aggregate of the shock, $\left(1-\phi^{\text{d}}\right)$, since in the Cobb–Douglas GE model shocks are aggregated with their sales shares. Note that here shocks only propagate downstream; in the upstream direction, price and quantity effects cancel each other; for details, see Refs. (16, 17). As a variant, we also calculate $L_{\mathrm{ind}.}^{\text{G}E}(\phi^{\max})=0.061$, where $\phi_{k}^{\max}=\max[\phi_{k}^{\text{d}},\phi_{k}^{\text{u}}]$.

## Supplementary Material

pgae064_Supplementary_Data

## Data Availability

The data are only physically available in the Central Bank of Hungary or the Hungarian Academy of Sciences and, hence, cannot be shared by the authors. The code to reproduce this study is available on https://github.com/ch-diem/misestimation_from_aggregation.

## Notes

a Official IOTs are constructed differently and are based on surveys and other data sources (68, 69).

b The index *t* denotes the “internal” time steps of the shock propagation model, *not* calendar time.

c We use January to May such that the shock corresponds to 2020Q1, as Hungary has a 2-month leave notice. See Methods.

## Preprints

A preprint of this article is available on https://arxiv.org/abs/2302.11451.
