# Supplementary material for: Estimating the loss of economic predictability from aggregating firm-level production networks
Source: PNAS Nexus. 2024 Feb 17;3(3):pgae064. doi: 10.1093/pnasnexus/pgae064 (PMC10965025; doi:10.1093/pnasnexus/pgae064)
Supplement: pgae064_Supplementary_Data [file pgae064_supplementary_data.pdf]

Supplementary Information:

Estimating the loss of economic predictability from aggregating firm-level production networks

Christian Diem, András Borsos, Tobias Reisch, János Kertész & Stefan Thurner

SI Section 1. Calculating input and output vectors

In this section we show how to calculate the intra-sector heterogeneity (or similarity) of the firms' input-output vectors. We start aggregating every firms' firm-level in- and output vector to the NACE2 industry-level. The ith column of the FPN's adjacency matrix, W, represents the firm-level input vector, $W_{i.}$, of firm i, while the ith row gives the firm-level output vector, $W_{.i}$. We compute the corresponding industry-level input vector, $\Pi^{\text{in}}_{i.}$, and output vector, $\Pi^{\text{out}}_{i.}$, of firm i, by aggregating all in links (purchases) of i's suppliers from the same industry and all out links (sales) to i's customers in the same industry, as

$$\Pi^{\text{in}}_{ik} = \sum_{j=1}^{m} W_{ji}\delta_{p_j,k}, \qquad \Pi^{\text{out}}_{ik} = \sum_{j=1}^{m} W_{ij}\delta_{p_j,k} \quad . \tag{S.1}$$

The element Π^{in}_{ik}, specifies the amount of input k firm i is buying from suppliers, j, of industry, k, i.e., all j with $p_j = k$. The element Π^{out}_{ik} specifies the amount firm i is selling to firms, j in industry, k, i.e., all j with $p_j = k$. The expression $\delta_{p_j k}$ is the Kronecker delta and is equal to one if firm j produces product k and zero otherwise, i.e.,

$$\delta_{p_j k} = \begin{cases} 1 & \text{if } \; p_j = k \quad , \\ 0 & \text{if } \; p_j \neq k \quad . \end{cases}$$

We focus on the relative importance of firms' input types (industries) and customer industries, independent of firm size. To do so, we compute the normalized input-, $\bar{\Pi}^{\text{in}}_{i.}$, and output vectors, $\bar{\Pi}^{\text{out}}_{i.}$, of every firm, i. The kth entry of the normalized input vector, $\bar{\Pi}^{\text{in}}_{ik}$, represents the fraction of inputs firm i buys from firms in industry k. Similarly, the kth entry of i's normalized output vector, $\bar{\Pi}^{\text{out}}_{ik}$, represents the fraction of firm i's revenue it receives by selling to firms in industry k. $\bar{\Pi}^{\text{in}}_{ik}$ and $\bar{\Pi}^{\text{out}}_{ik}$ are the scaled technical and allocation coefficients in classical IO analysis. For firm i the vectors $\bar{\Pi}^{\text{in}}_{i.}$ and $\bar{\Pi}^{\text{out}}_{i.}$ are computed as

$$\bar{\Pi}^{\text{in}}_{ik} = \frac{\Pi^{\text{in}}_{ik}}{\sum_{k=1}^{m}\Pi^{\text{in}}_{ik}} = \frac{1}{s^{\text{in}}_i}\sum_{j=1}^{m} W_{ji}\delta_{p_j,k} \quad , \qquad \bar{\Pi}^{\text{out}}_{ik} = \frac{\Pi^{\text{out}}_{ik}}{\sum_{k=1}^{m}\Pi^{\text{out}}_{ik}} = \frac{1}{s^{\text{out}}_i}\sum_{j=1}^{m} W_{ij}\delta_{p_j,k} \quad , \tag{S.2}$$

where $\delta_{p_j,k} = 1$ if firm j belongs to industry k and $\delta_{p_j,k} = 0$ otherwise. We quantify the similarity between input and output vectors of two firms with the *overlap coefficient* (OC) due to its clear economic interpretability. To show that our results do not depend on the specific choice of the similarity measure we also look at the jaccard index (JI).

SI Section 2. Details for calculation and interpretation of the input and output overlap coefficient

In general the overlap coefficient of two vectors x, y of dimension m is defined as

$$\text{OC}\,(x,\; y) = \frac{\sum_{k=1}^{m}\min\left[x_k, y_k\right]}{\min\left[\sum_{k=1}^{m} x_k,\; \sum_{k=1}^{m} y_k\right]} \quad . \tag{S.3}$$

We calculate the overlap coefficient of the 1-norm $||\;||_1$ normalized input, and output vectors, i.e. $\bar{\Pi}^{\text{in}}_i$ and $\bar{\Pi}^{\text{out}}_i$. Therefore, in each calculation both vectors sum to one, then the denominator is always equal to one and can be dropped. The overlap coefficient is closely related to the *weighted* Jaccard Index, which has the same numerator, and $\sum_{k=1}^{m}\max\left[x_k, y_k\right]$ as the denominator. It is also called the Szymkiewicz-Simpson distance [1, 2].

As introduced in the main text, for our application we calculate the input overlap coefficient (IOC) and output overlap coefficient (OOC) of two firms i and j as,

$$\text{IOC}_{ij} \;=\; \sum_{k=1}^{m}\min\left[\bar{\Pi}^{\text{in}}_{ik},\; \bar{\Pi}^{\text{in}}_{jk}\right] \quad , \tag{S.4}$$

$$\text{OOC}_{ij} \;=\; \sum_{k=1}^{m}\min\left[\bar{\Pi}^{\text{out}}_{ik},\; \bar{\Pi}^{\text{out}}_{jk}\right] \quad . \tag{S.5}$$

The denominator from Eq. S.3 can be omitted since $\sum_{k=1}^{m}\bar{\Pi}^{\text{in}}_{ik} = 1$ and $\sum_{k=1}^{m}\bar{\Pi}^{\text{out}}_{ik} = 1$ for all i. We calculate the distribution of the two measures for each industry k, by computing all pairwise IOC_{ij} and OOC_{ij} for all firms where, $p_i = p_j = k$, and $i \neq j$, in

the respective industry, k. The input overlap coefficient, IOC_{ij}, of two firms i and j gives the fraction of their overall inputs they source from the same industries, i.e. the overlap of their industry input shares. The output overlap coefficient, OOC_{ij}, of two firms i and j specifies the fraction of their overall sales they sell to the same industries, i.e. the overlap of their industry sales shares. Note that IOC_{ij} also quantifies i's and j's overlap of exposures to other economic dynamics, like price increases or innovations of supplying industries. Similarly, OOC_{ij} measures the common exposure to, e.g., innovation in the buyer industry that makes the input of firms obsolete.

In the example of Fig. 1, the relative input vector of firm 10 is $\bar{\Pi}^{\mathrm{in}}_{10} = (0, 0, 0.5, 0.5, 0)$ and for firm 11 $\bar{\Pi}^{\mathrm{in}}_{11} = (0, 0, 1, 0, 0)$, hence $\mathrm{IOC}_{10,11} = 0.5$. If a demand shock affects firm 10, 50% of the shock spreads upstream to sectors 3 and 4, respectively, while if the shock affects firm 11, 100% of the shock spreads upstream to sector 3. This means that the shock spreading dynamics overlap by only 50% ($\mathrm{IOC}_{10,11} = 0.5$), while the other 50% spread to distinct sectors. For firms 6 and 7 the output vectors are $\bar{\Pi}^{\mathrm{out}}_{6} = (1, 0, 0, 0, 0)$ and $\bar{\Pi}^{\mathrm{out}}_{7} = (0, 0, 0, 0, 1)$, hence, $\mathrm{OOC}_{6,7} = 0$. This means that if either, 6 or 7, receives a shock, 0% of the shock would affect the same industry and 100% would spread to different sectors, in one case towards sector 1 and in the other to sector 5. Also their exposure to demand shocks has no overlap. Firm 6 is only exposed to industry 1 while firm 6 is only exposed to sector 5.

Since we use industry-level input and output vectors, which neglect firm-level differences within industries, the real level of heterogeneity could be even larger. In our dataset cross border import and export links of firms are not available. This could lead to a potential underestimation of overlaps, but a study for Belgium [3] shows that firms' import and export links are few in relation to national import and export links.

SI Section 3. Further results on input and output overlaps

Further results for NACE C26

Even though, in all four in-degree (out-degree) groups there are firms with very similar input (output) vectors, the results clearly show that in general firms have surprisingly small overlaps with respect to to their suppliers' industries (inputs) and customers' (output) industries. This implies that if two random firms in in-degree (out-degree) bin >35 receive the same absolute size shock, on average only 34% (14%) of the shock's volume is propagated to firms of the same industry while 66% (86%) of the shock is propagated to firms in other industries. At the same time it means that two firms in this industry have on average 66% (86%) of their upstream (downstream) exposures to different supplier (buyer) industries. The low level of similarity of input and output vectors clearly shows that aggregating these firms into a single industry is not representative of the single firms' input-output vectors and will lead to large biases and mis-estimations of economic dynamics.

Further results on output overlaps across industries

The highest median OOC_{ij} are found in Veterinary activities (M75), Manufacture of beverages (C11), Manufacture of other transport equipment (C30), Forestry and logging (A2), Manufacture of leather and related products (C15), Manufacture of basic pharmaceutical products (C21), Telecommunications (J61), whereas the lowest median OOC_{ij} are found in service sectors such as Public administration and defence; compulsory social security (O84), Travel agency and related activities (N79), or Scientific research and development (M72), but also non-service sectors such as Remediation activities and other waste management services (E39), Other manufacturing (C32), or Manufacture of textiles (C13) are among the lowest output overlap sectors. The average standard deviation is 0.17, the standard deviation of standard deviations is 0.047, and the error bar length appears to be relatively homogeneous across sectors. This indicates that the variation of pairwise output overlaps, OOC_{ij}, within sectors is relatively similar across sectors. For the other degree bins see SI Fig. S6.

The same results are shown for the other three out-degree bins 1-5, 6-15, and 16-35 in SI Section 4 Fig. S3. As for industry C26, the output overlaps are smaller for lower degree bins; the averages over the mean (median) output overlaps, are 0.110, (0.021) 0.157 (0.135), 0.223 (0.215), for the bins 1-5, 6-15, and 16-35, respectively. The averages over the standard deviations of output overlaps, are 0.266, 0.129, 0.109, respectively and therefore the variation of output overlaps within is on average decreasing with the number of out-links. Figure S1b illustrates this relationship more clearly by showing for each in-degree size bin (1-5, 6-15, 16-35, >35) the boxplot of the industries' median OOC values. It is clearly visible that output vectors of firms within industries become more homogeneous with the number of suppliers. SI Section 5 shows that OOC_{ij} are even lower when computed for at NACE 4 level. SI Fig. S4b shows the average of the mean (median) output overlaps, across NACE4 industries is 0.231 (0.207). The standard deviation of mean (median) output overlaps is 0.179 (0.19), i.e. higher than for the NACE2 level. This indicates that the variation of average output vector overlaps is higher at the NACE 4 level. The average standard deviation is 0.126 and the standard deviation of standard deviations is 0.056. Note that the average IOC and OOC levels seem to be more similar on the NACE 4 level than at the NACE 2 level where the average IOC is higher than OOC. For the other degree bins see SI Fig. S7. SI Fig. S9 in SI Section 6 shows qualitatively similar results for the Jaccard Index for the degree bin >35. The pairwise input Jaccard Index (IJI) distributions are slightly shifted towards higher similarity values with a average mean (median), 0.398 (0.394) and slightly less variation with a standard deviation of means of 0.07 (0.067). The pairwise output Jaccard Index (OJI) distributions are also shifted towards slightly higher similarity values with a average over means (medians), of 0.301 (0.291) and slightly less variation with a standard deviation of means of 0.076 (0.077).

SI Section 4. Overlap coefficients across industries for other degree bins

This section shows the results of the pairwise input overlap coefficient, IOC, and output overlap coefficient, OOC, distributions across all NACE2 industries for the three degree bins 1-5, 6-15, and 16-35 that are not shown in Fig. 3. As for Fig. 3 we calculate the summary statistics — mean, 5%, 25%, 50% (median) 75% and 95% percentiles — for the pairwise IOC (SI Fig. S2) and OOC (SI Fig. S3) distributions for all NACE2 industries. Again these statistics are visualized as boxplots. The x-axis shows the 86 NACE2 codes present in the data set; the y-axis denotes the overlap coefficients, each boxplot corresponds to a NACE2 class. The dark thick horizontal bars correspond to the median, ($p_{5\%}$), the interquartile range ($p_{25\%} - p_{75\%}$) is shown as thick dark vertical lines, and the error bars ($p_{5\%} - p_{95\%}$) are indicated by thin light vertical lines. The thin vertical black lines separate NACE2 classes by their NACE1 affiliation.

The results for the IOC distributions are shown in SI Fig. S2. SI Fig. S2a shows the pairwise IOC$_{ij}$ for firms with in-degree between one and five, $1 \leq k_i^{in} \leq 5$. The mean over the industries' mean (median) IOC is 0.132 (0.009), the standard deviation of mean (median) IOCs is 0.081 (0.062). The mean standard deviation is 0.262. SI Fig. S2b shows the pairwise IOC$_{ij}$ for firms with in-degree between one and five, $6 \leq k_i^{in} \leq 15$. The mean over the industries' mean (median) IOC is 0.202 (0.148), the standard deviation of mean (median) IOCs is 0.081 (0.088). The mean standard deviation is 0.192. SI Fig. S2c shows the pairwise IOC$_{ij}$ for firms with in-degree between one and five, $16 \leq k_i^{in} \leq 35$. The mean over the industries' mean (median) IOC is 0.269 (0.241), the standard deviation of mean (median) IOCs is 0.083 (0.091). The mean standard deviation is 0.168. As for NACE C26 in the main text we see that on average input vector overlaps increase with the number of suppliers.

The results for the OOC distributions are shown in SI Fig. S3. SI Fig. S3a shows the pairwise OOC$_{ij}$ for firms with out-degree between one and five, $1 \leq k_i^{out} \leq 5$. The mean over the industries' mean (median) OOC is 0.110 (0.021), the standard deviation of mean (median) OOCs is 0.094 (0.118). The mean standard deviation is 0.226. SI Fig. S3b shows the pairwise OOC$_{ij}$ for firms with out-degree between one and five, $6 \leq k_i^{out} \leq 15$. The mean over the industries' mean (median) OOC is 0.157 (0.135), the standard deviation of mean (median) OOCs is 0.078 (0.074). The mean standard deviation is 0.129. SI Fig. S3c shows the pairwise OOC$_{ij}$ for firms with out-degree between one and five, $16 \leq k_i^{out} \leq 35$. The mean over the industries' mean (median) OOC is 0.223 (0.215), the standard deviation of mean (median) OOCs is 0.078 (0.078). The mean standard deviation is 0.109. Again average overlaps seem to increase with degree (number of customers). Further, output overlaps are on average slightly lower than input overlaps.

Next we illustrate how average similarity increases with the degree bins. Fig. S1 illustrates this relationship more clearly by showing for each degree size bin (1-5, 6-15, 16-35, >35) the boxplot of the industries' median IOC and OOC values. Fig. S1a shows boxplots of the median input overlap coefficients, IOC, for all NACE2 industries for each in-degree bin, respectively. We see that for the bin with 1 to 5 suppliers almost all medians are zero. Then the distribution of medians is substantially shifted upwards for the bin of 6-15 suppliers and it continues to increase for the other two in-degree bins with 16-35 and more than 35 suppliers, respectively. Fig. S1b shows boxplots of the median output overlap coefficients, OOC, for all NACE2 industries for each out-degree bin, respectively. We see that for the bin with 1 to 5 buyers almost all medians are zero. Then the distribution of medians is slightly shifted upwards for the bin of 6-15 buyers, but there are several outlier industries with higher output overlaps. The median OOC continue to increase for the other two out-degree bins with 16-35 and more than 35 buyers, respectively. It is visible that the upper tails of the median OOC distributions are longer than for the median IOC distributions. Overall median OOCs are lower than median IOCs.

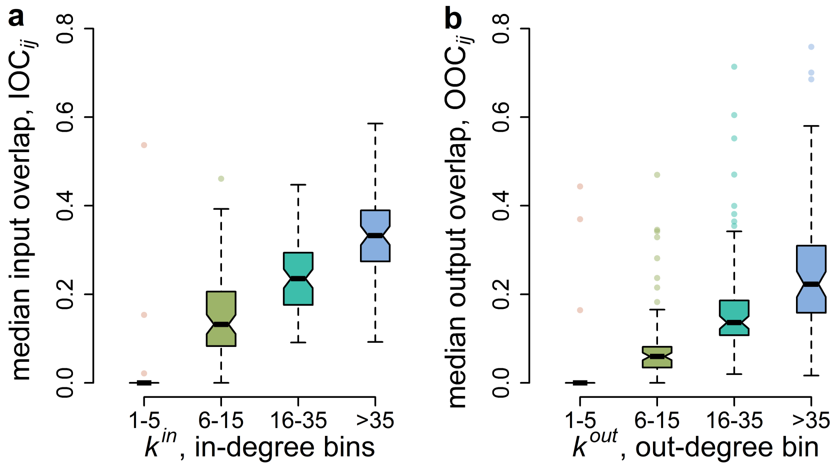

Figure S1. Increase of input- and output-vector similarity with increasing in-degree, k^{in}, and out-degree, k^{out}, bins (1-5, 6-15, 16-35, >35). a) boxplots of the median input overlap coefficients for all NACE2 industries for each in-degree bins, respectively. b) boxplots of the median out overlap coefficients for all NACE2 industries for each out-degree bins, respectively. It is clearly visible that input- and output-vectors of firms within industries become on average more similar (higher median IOC and OOC values) with the number of suppliers and buyers.

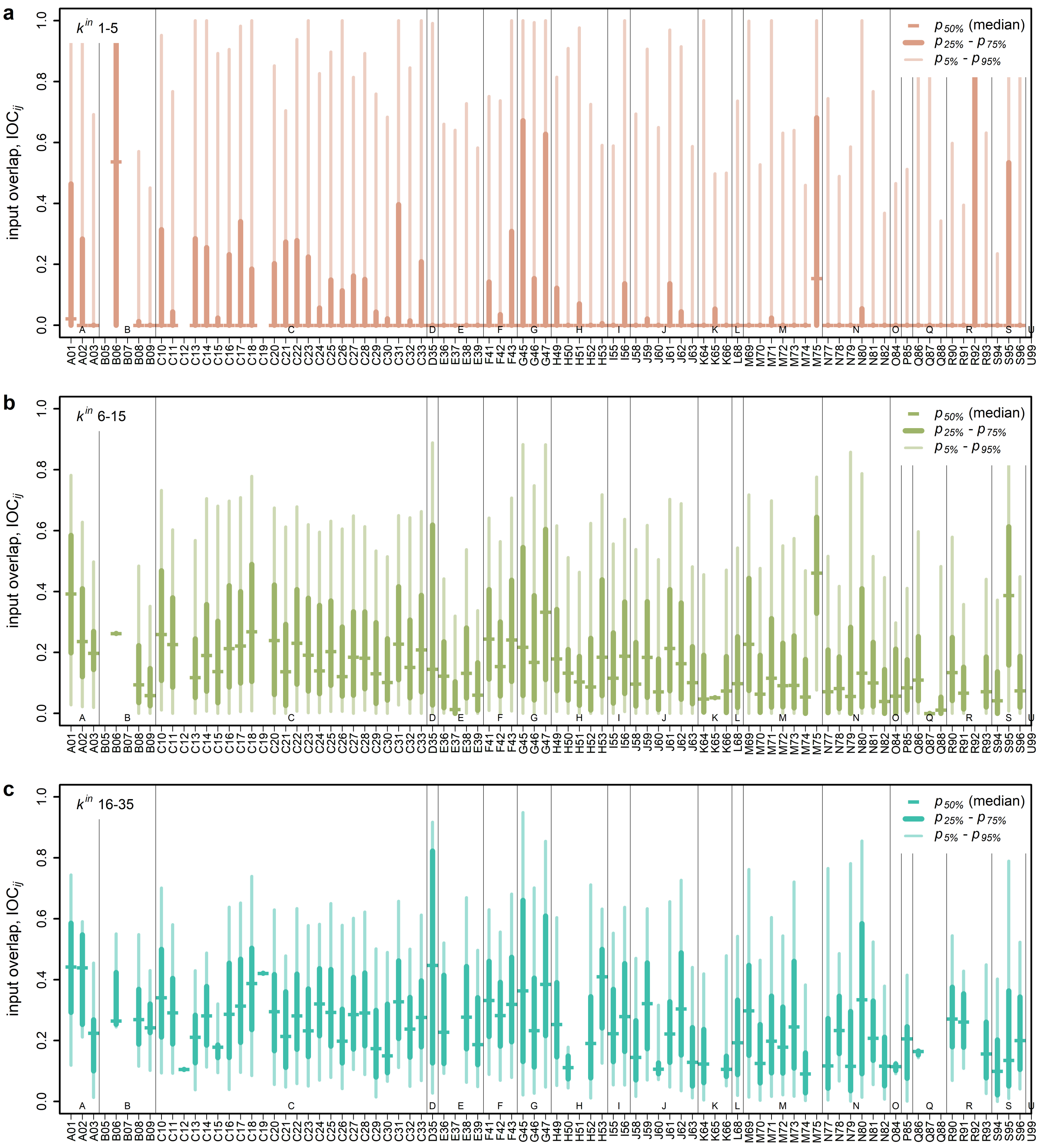

Figure S2. Distributions of pairwise input vector overlaps, IOC_{ij}, of firms across NACE 2 industries for three in-degree size bins. NACE2 classes are on the x-axis; overlap coefficients on the y-axis. a) pairwise IOC_{ij} for firms with in-degree between one and five, $1 \leq k_i^{in} \leq 5$. The mean over the industries' mean (median) IOC is 0.132 (0.009), the standard deviation of mean (median) IOCs is 0.081 (0.062). The mean standard deviation is 0.262. b) pairwise IOC_{ij} for firms with in-degree between 6 and 15, $6 \leq k_i^{in} \leq 15$. The mean over the industries' mean (median) IOC is 0.202 (0.148), the standard deviation of mean (median) IOCs is 0.081 (0.088). The mean standard deviation is 0.192. c) pairwise IOC_{ij} for firms with in-degree between 16 and 35, $16 \leq k_i^{in} \leq 35$. The mean over the industries' mean (median) IOC is 0.269 (0.241), the standard deviation of mean (median) IOCs is 0.083 (0.091). The mean standard deviation is 0.168.

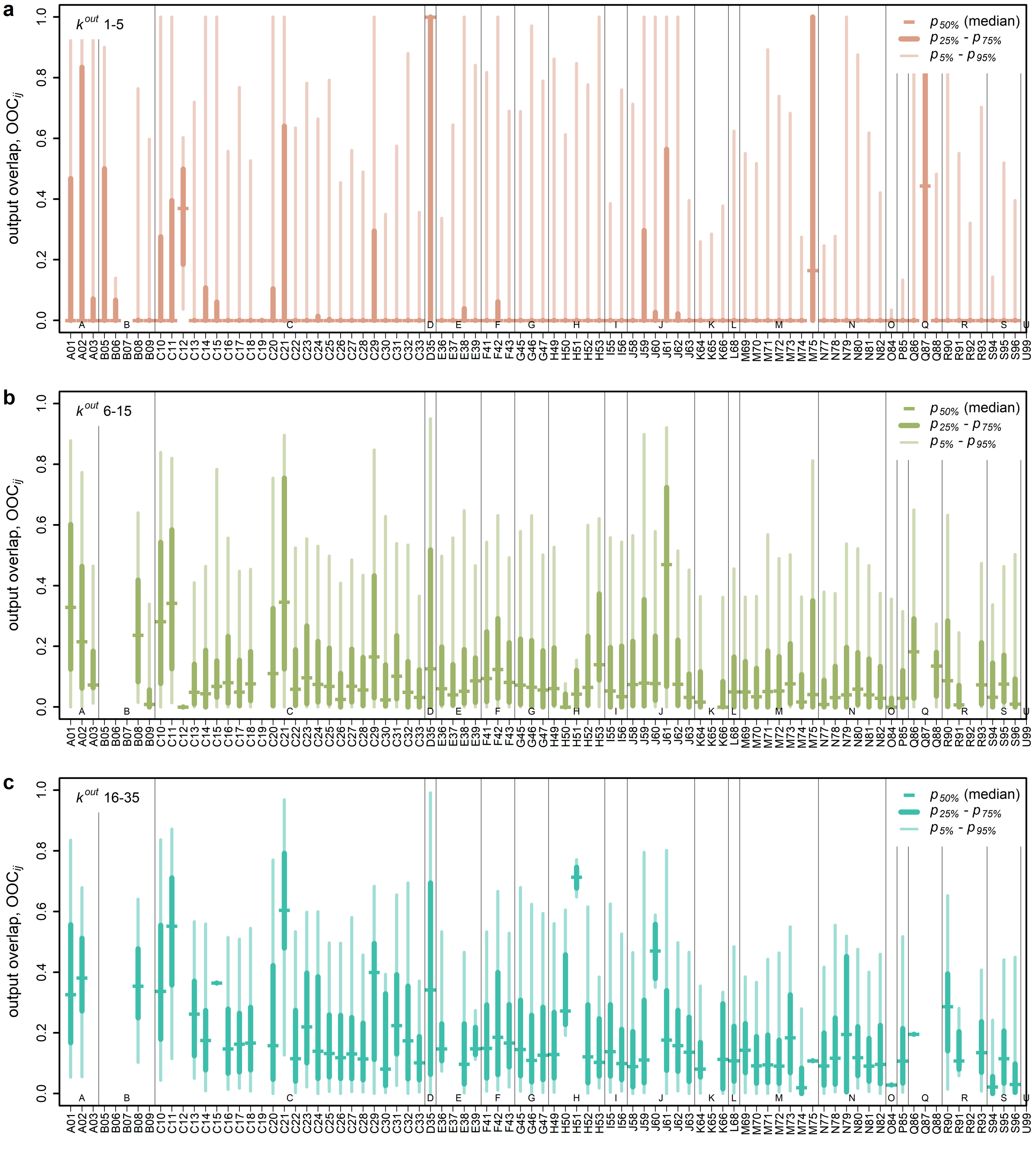

Figure S3. Distributions of pairwise output vector overlaps, OOC_{ij}, of firms across NACE 2 industries for three in-degree size bins. NACE2 classes are on the x-axis; overlap coefficients on the y-axis. a) pairwise OOC_{ij} for firms with out-degree between one and five, $1 \leq k_i^{out} \leq 5$. The mean over the industries' mean (median) OOC is 0.110 (0.021), the standard deviation of mean (median) OOCs is 0.094 (0.118). The mean standard deviation is 0.226. b) pairwise OOC_{ij} for firms with out-degree between 6 and 15, $6 \leq k_i^{out} \leq 15$. The mean over the industries' mean (median) OOC is 0.157 (0.135), the standard deviation of mean (median) OOCs is 0.078 (0.074). The mean standard deviation is 0.129. c) pairwise OOC_{ij} for firms with out-degree between 16 and 35, $16 \leq k_i^{out} \leq 35$. The mean over the industries' mean (median) OOC is 0.223 (0.215), the standard deviation of mean (median) OOCs is 0.078 (0.078). The mean standard deviation is 0.109.

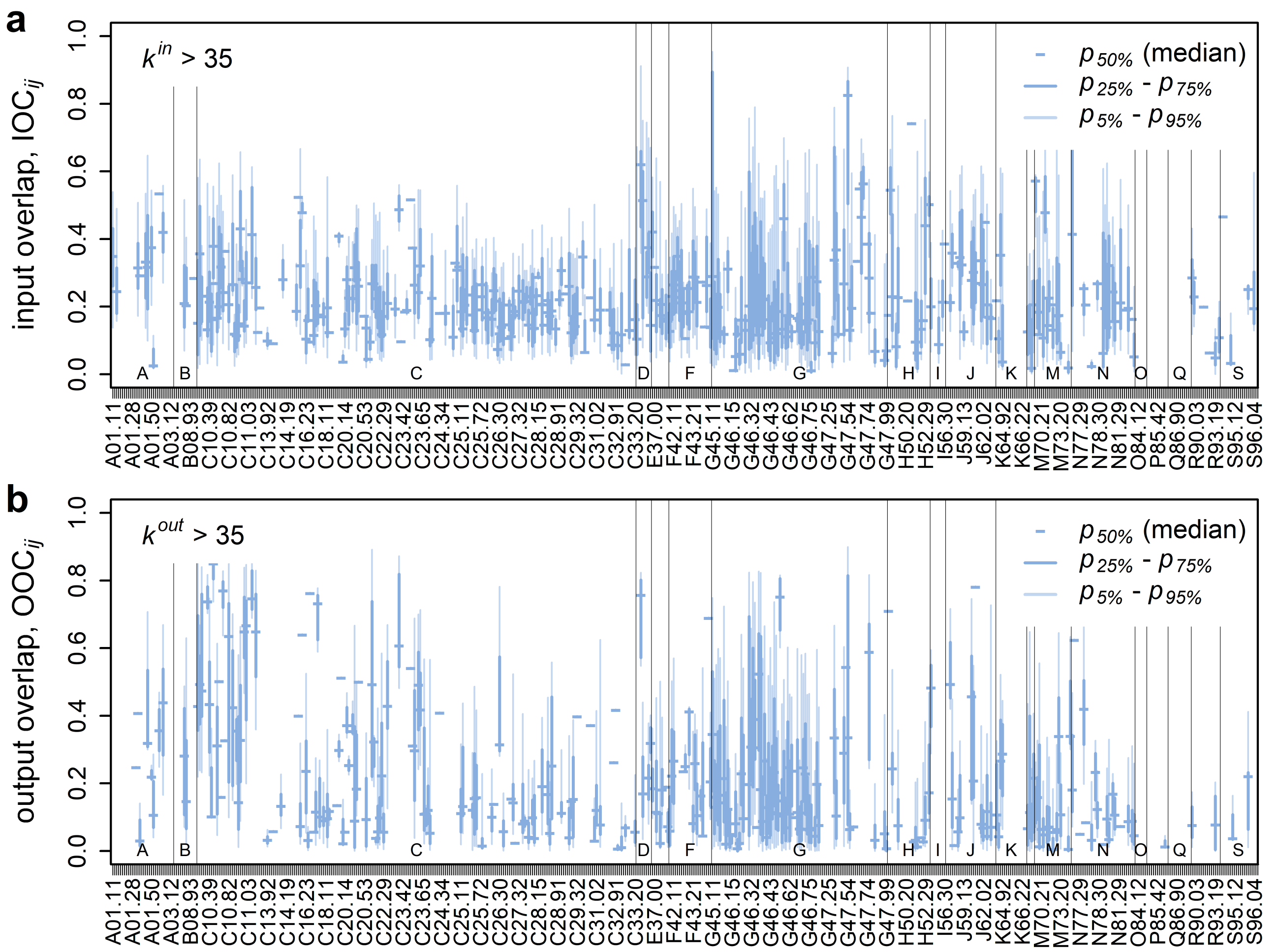

Figure S4. Pairwise similarity distributions of input- and output-vectors of firms within each NACE4 industry. Similarity is measured with the overlap coefficient for firms with more than 35 suppliers (a) and buyers (b), respectively. The y-axis denotes the overlap coefficients, the x-axis shows the NACE4 code for the respective boxplots. The dark blue horizontal bars correspond to the median, ($p_{50\%}$), dark blue vertical lines to the interquartile range ($p_{25\%} - p_{75\%}$), and thin light blue vertical lines to error bars ($p_{5\%} - p_{95\%}$). Thin black vertical lines separate NACE1 classes. Empty columns indicate sectors with less than two firms in this degree bin. a) distributions of pairwise intra-industry input overlap coefficients, IOC$_{ij}$. The average of the mean (median) input overlaps, across NACE2 industries is 0.237 (0.216) and the standard deviation of mean (median) input overlaps is 0.11 (0.12). The average standard deviation is 0.126. This indicates that relatively low input overlaps are the norm, but there are several outliers with higher similarities. b) distributions of pairwise intra-industry output overlap coefficients, OOC$_{ij}$. The average of the mean (median) output overlaps, across NACE2 industries is 0.231 (0.207) and the standard deviation of mean (median) output overlaps is 0.179 (0.19), indicating that relatively low output overlaps are the norm, but there are relatively many outliers with higher similarities. The average standard deviation is 0.135. Output overlaps are on average only slightly lower than the input overlaps, but there is more variation across industries. If industry-level aggregation were fully representative for the IO-vectors of firms in both panels all distributions would correspond to a single bar at the value 1.

SI Section 5. Overlap coefficients for NACE 4 level input output vectors

In this section we show that the pairwise input overlaps, IOC$_{ij}$, and output overlaps, OOC$_{ij}$, are lower for all pairs of firms within NACE 4 industries for the NACE 4 level input and output vectors. Remember, in the previous analysis we have computed the overlaps for all pairs of firms within a NACE2 industry and on the NACE2 level input and output vectors.

In the following figures we show the pairwise overlap coefficient distributions of input- and output-vectors of firms within each NACE4 industry for the respective degree-bins 1-5, 6-15, 6-35, and >35. The y-axis denotes the overlap coefficients, the x-axis shows the NACE4 code for the respective boxplots. The dark horizontal bars correspond to the median, ($p_{50\%}$), dark vertical lines to the interquartile range ($p_{25\%} - p_{75\%}$), and thin light vertical lines to error bars ($p_{5\%} - p_{95\%}$). Thin black vertical lines separate NACE1 classes. Empty columns indicate sectors with less than two firms in this degree bin.

First, we show the distributions of the input overlap coefficients, IOC$_{ij}$. SI Fig. S6a shows the distributions of pairwise intra-industry input overlap coefficients, IOC$_{ij}$, for firms with more than 35 suppliers, $k^{\text{in}} > 35$. The average of the mean (median) input overlaps, across NACE2 industries is 0.237 (0.216) and the standard deviation of mean (median) input overlaps is 0.11 (0.12). The average standard deviation is 0.126. This indicates that relatively low input overlaps are the norm, but there are several outliers

with higher similarities. SI Fig. S6a shows the distributions of pairwise input overlap coefficients, IOC_{ij}, for firms with in-degree between one and five, $1 \leq k_i^{in} \leq 5$. The mean over the industries' mean (median) IOC_{ij} is 0.063 (0.005), the standard deviation of mean (median) IOCs is 0.074 (0.057). The mean standard deviation is 0.168. SI Fig. S6b shows the distributions of pairwise input overlap coefficients, IOC_{ij}, for firms with in-degree between 6 and 15, $6 \leq k_i^{in} \leq 15$. The mean over the industries' mean (median) IOC_{ij} is 0.112 (0.063), the standard deviation of mean (median) IOCs is 0.084 (0.083). The mean standard deviation is 0.139. SI Fig. S6c shows the distributions of pairwise input overlap coefficients, IOC_{ij}, for firms with in-degree between 16 and 35, $16 \leq k_i^{in} \leq 35$. The mean over the industries' mean (median) IOC_{ij} is 0.165 (0.140), the standard deviation of mean (median) IOCs is 0.107 (0.112). The mean standard deviation is 0.130.

Second, we show the distributions of the output overlap coefficients, OOC_{ij}. SI Fig. S6b shows the distributions of pairwise intra-industry output overlap coefficients, OOC_{ij}, for more than 35 buyers, $k^{\mathrm{out}} > 35$. The average of the mean (median) output overlaps, across NACE2 industries is 0.231 (0.207) and the standard deviation of mean (median) output overlaps is 0.179 (0.19), indicating that relatively low output overlaps are the norm, but there are relatively many outliers with higher similarities. The average standard deviation is 0.135. Output overlaps are on average only slightly lower than the input overlaps, but there is more variation across industries. SI Fig. S7a shows the distributions of pairwise output overlap coefficients, OOC_{ij}, for firms with out-degree between one and five, $1 \leq k_i^{out} \leq 5$. The mean over the industries' mean (median) OOC_{ij} is 0.056 (0.005), the standard deviation of mean (median) OOCs is 0.075 (0.054). The mean standard deviation is 0.148. SI Fig. S7b shows the distributions of pairwise output overlap coefficients, OOC_{ij}, for firms with out-degree between 6 and 15, $6 \leq k_i^{out} \leq 15$. The mean over the industries' mean (median) OOC_{ij} is 0.081 (0.057), the standard deviation of mean (median) OOCs is 0.063 (0.068). The mean standard deviation is 0.087. SI Fig. S7c shows the distributions of pairwise output overlap coefficients, OOC_{ij}, for firms with out-degree between 16 and 35, $16 \leq k_i^{out} \leq 35$. The mean over the industries' mean (median) OOC_{ij} is 0.127 (0.116), the standard deviation of mean (median) OOCs is 0.080 (0.082). The mean standard deviation is 0.078. Note that if industry-level aggregation was fully representative for the IO-vectors of firms in all figures all distributions would correspond to a single bar at the value 1.

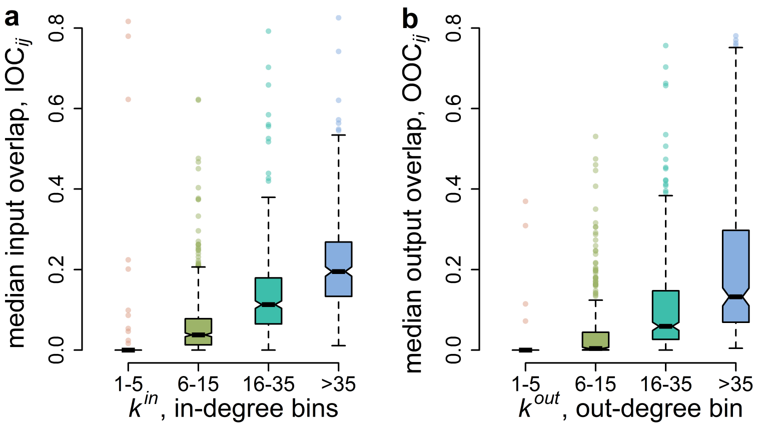

Figure S5. Increase of input- and output-vector similarity with increasing in-degree, k^{in}, and out-degree, k^{out}, bins (1-5, 6-15, 16-35, >35). a) boxplots of the median input overlap coefficients for all NACE 4 industries for each in-degree bin, respectively. b) boxplots of the median out overlap coefficients for all NACE 4 industries for each out-degree bin, respectively. It is clearly visible that input- and output-vectors of firms within industries become on average more similar (higher median IOC and OOC values) with the number of suppliers and buyers.

Next we show specifically how the average overlap coefficients increase with the degree of firms. Fig. S5 illustrates this relationship by showing for each degree size bin (1-5, 6-15, 16-35, >35) on the x-axis, the boxplot of the NACE 4 industries' median overlap coefficients on the y-axis. Fig. S5a shows boxplots of the median input overlap coefficients, IOC_{ij}, for all NACE4 industries for each in-degree bin, respectively. We see that for the bin with 1 to 5 suppliers most medians are zero. Then the distribution of medians is shifted upwards for the bin of 6-15 suppliers and it continues to increase for the other two in-dgree bins with 16-35 and more than 35 suppliers, respectively. Note that even for two highest degree bins medians can range from almost zero to above 0.8. Fig. S5b shows boxplots of the median output overlap coefficients, OOC_{ij}, for all NACE2 industries for each out-degree bin, respectively. We see that for the bin with 1 to 5 buyers almost all medians are zero. Then the distribution of medians is slightly shifted upwards for the bin of 6-15 buyers, but there are several outlier industries with higher output overlaps. The median OOC continue to increase for the other two out-dgree bins with 16-35 and more than 35 buyers, respectively. Note that even for two highest degree bins medians can range from zero to around 0.8. It is visible that the tails of the median OOC distributions are longer than for the median IOC distributions. Overall median OOCs appear lower than median IOCs.

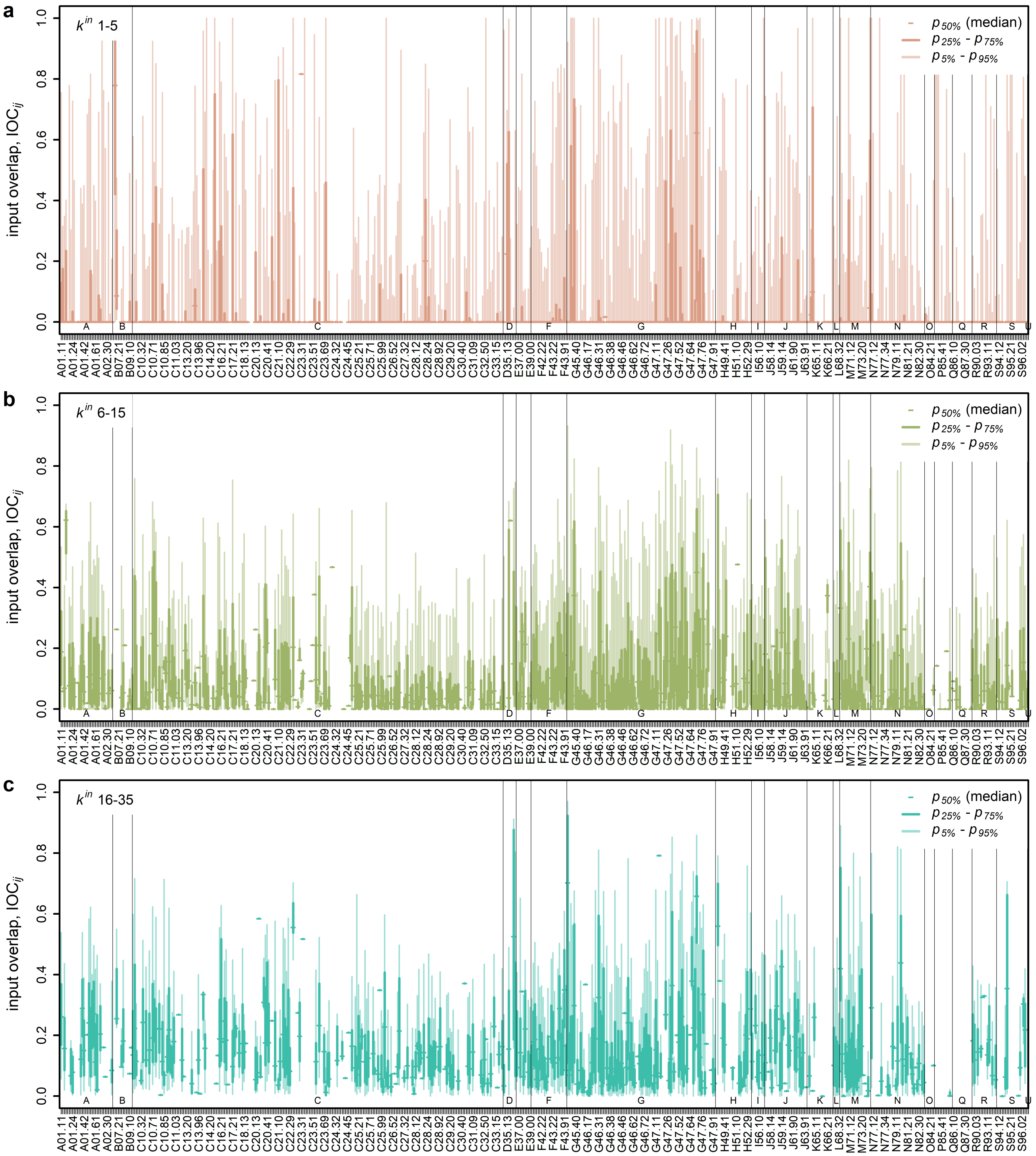

Figure S6. Distributions of pairwise input vector overlaps, IOC_{ij}, of firms across NACE 4 industries for three in-degree size bins. NACE 4 classes are on the x-axis; overlap coefficients on the y-axis. a) pairwise IOC_{ij} for firms with in-degree between one and five, $1 \leq k_i^{in} \leq 5$. The mean over the industries' mean (median) IOC is 0.063 (0.005), the standard deviation of mean (median) IOCs is 0.074 (0.057). The mean standard deviation is 0.168. b) pairwise IOC_{ij} for firms with in-degree between 6 and 15, $6 \leq k_i^{in} \leq 15$. The mean over the industries' mean (median) IOC is 0.112 (0.063), the standard deviation of mean (median) IOCs is 0.084 (0.083). The mean standard deviation is 0.139. c) pairwise IOC_{ij} for firms with in-degree between 16 and 35, $16 \leq k_i^{in} \leq 35$. The mean over the industries' mean (median) IOC is 0.165 (0.140), the standard deviation of mean (median) IOCs is 0.107 (0.112). The mean standard deviation is 0.130.

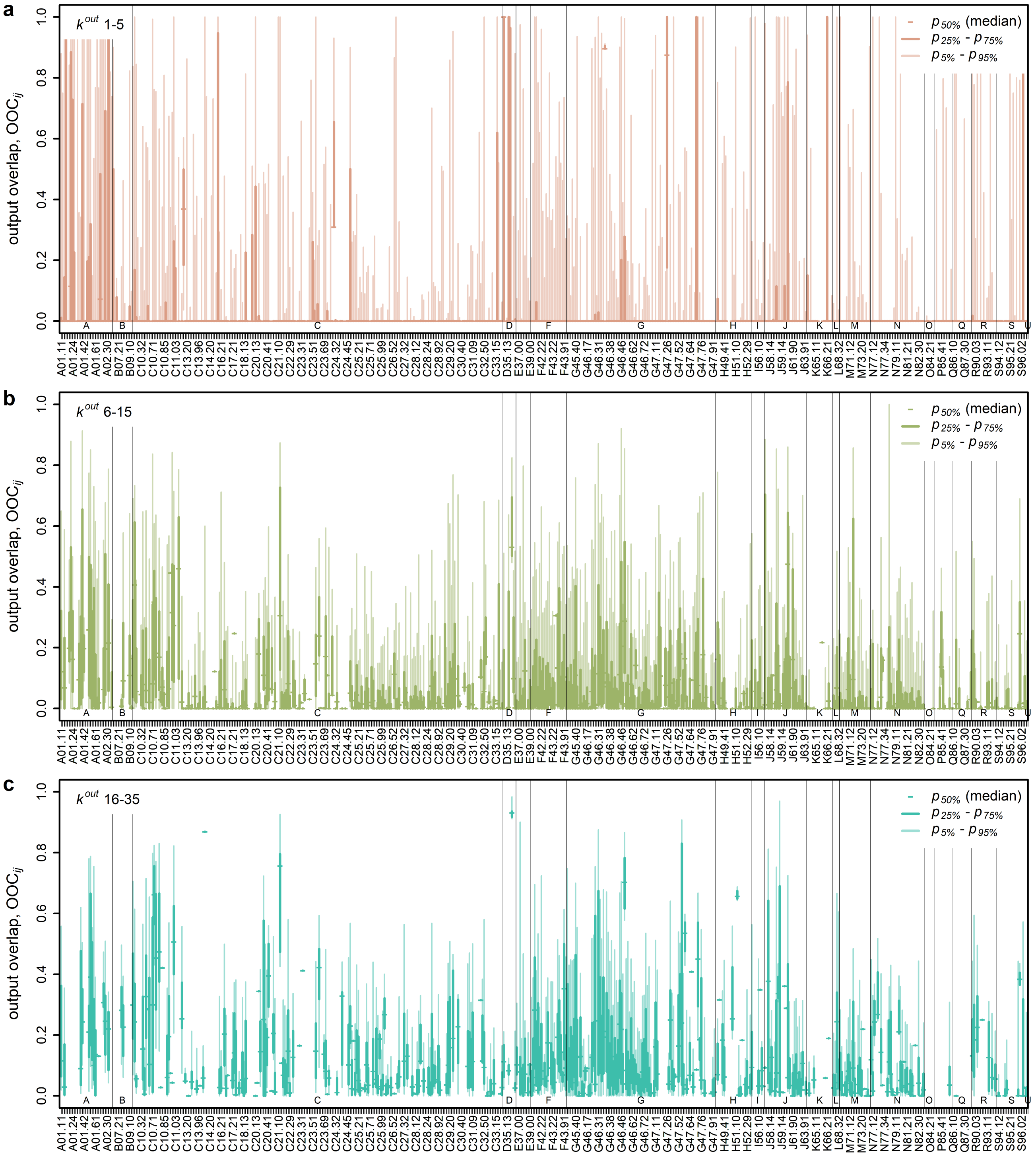

Figure S7. Distributions of pairwise output vector overlaps, OOC_{ij}, of firms across NACE 4 industries for three in-degree size bins. NACE 4 classes are on the x-axis; overlap coefficients on the y-axis. a) pairwise OOC_{ij} for firms with out-degree between one and five, $1 \leq k_i^{out} \leq 5$. The mean over the industries' mean (median) OOC is 0.056 (0.005), the standard deviation of mean (median) OOCs is 0.075 (0.054). The mean standard deviation is 0.148. b) pairwise OOC_{ij} for firms with out-degree between 6 and 15, $6 \leq k_i^{out} \leq 15$. The mean over the industries' mean (median) OOC is 0.081 (0.057), the standard deviation of mean (median) OOCs is 0.063 (0.068). The mean standard deviation is 0.087. c) pairwise OOC_{ij} for firms with out-degree between 16 and 35, $16 \leq k_i^{out} \leq 35$. The mean over the industries' mean (median) OOC is 0.127 (0.116), the standard deviation of mean (median) OOCs is 0.080 (0.082). The mean standard deviation is 0.078.

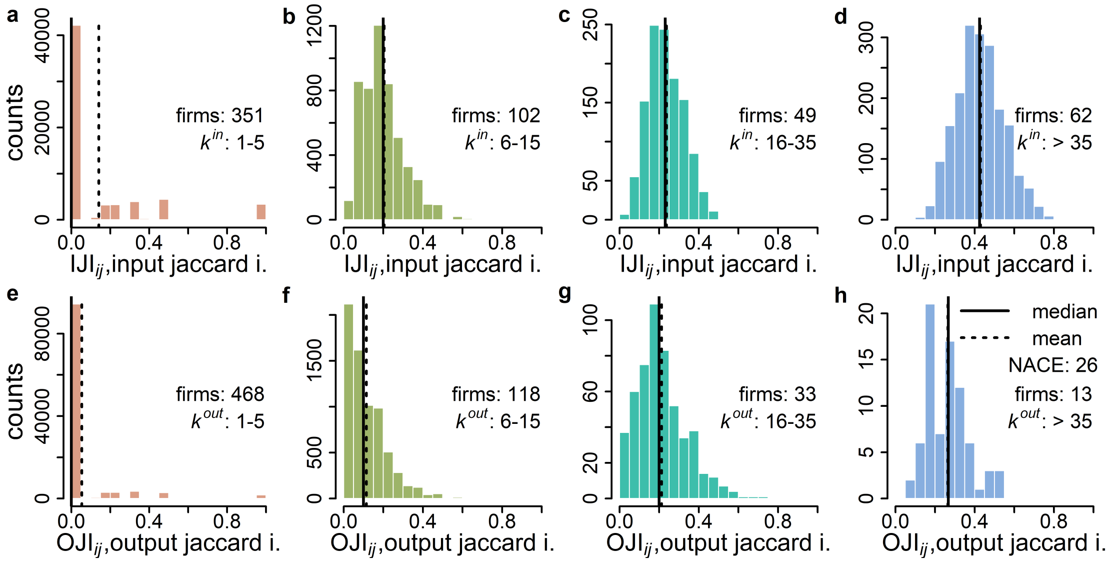

Figure S8. Pairwise similarity distributions of input and output vectors for firms of NACE class 26, Manufacture of computer, electronic and optical products measured with the Jaccard Index. a-d) show input Jaccard Indices, IJI_{ij}, and e-h) output Jaccard Indices, OJI_{ij}, visualized as histograms, for four in-degree, k_i^{in}, (number of suppliers) and out-degree, k_i^{out} (number of buyers), bins, respectively. Jaccard Index values are on the x-axis in bins of width 0.05; the y-axis shows the frequency to fall in the respective bin. Vertical solid lines correspond to median and dashed lines to mean overlap coefficients. a) pairwise IJI_{ij} for 351 firms with $1 \leq k_i^{in} \leq 5$. The median and mean input Jaccard Index is 0 and 0.141, respectively; the standard deviation is 0.261. b) pairwise IJI_{ij} for 102 firms with $6 \leq k_i^{in} \leq 15$. The median and mean input Jaccard Index is 0.2 and 0.204, respectively; the standard deviation is 0.109. c) pairwise IJI_{ij} for 49 firms with $16 \leq k_i^{in} \leq 35$. The median and mean input Jaccard Index is 0.231 and 0.237, respectively; the standard deviation is 0.091. d) pairwise IJI_{ij} for 62 firms with $35 < k_i^{in}$. The median and mean input Jaccard Index is 0.425 and 0.43, respectively; the standard deviation is 0.119. It is clearly visible that the similarity of input vectors is low for all size bins, but increases on average with the number of suppliers. e) pairwise OJI_{ij} for 468 firms with $1 \leq k_i^{out} \leq 5$. The median and mean output Jaccard Index is 0 and 0.054, respectively; the standard deviation is 0.163. f) pairwise OJI_{ij} for 118 firms with $6 \leq k_i^{out} \leq 15$. The median and mean output Jaccard Index is 0.1 and 0.115, respectively; the standard deviation is 0.109. g) pairwise OJI_{ij} for 33 firms with $16 \leq k_i^{out} \leq 35$. The median and mean output Jaccard Index is 0.2 and 0.212, respectively; the standard deviation is 0.127 . h) pairwise OJI_{ij} for 13 firms with $35 < k_i^{out}$. The median and mean output Jaccard Index is 0.267 and 0.265, respectively; the standard deviation is 0.106. The similarity of output vectors is even lower than for input vectors, and also increases on average with the number of buyers. If industry-level aggregation were fully representative for the IO-vectors of firms in NACE C26 in all panels the distributions would correspond to a single bar at the value 1.

SI Section 6. Jaccard Index confirms low similarities

The Jaccard Index for two binary vectors x, y of dimension m can be defined as

$$\text{JI}\,(x,\ y) = \frac{\sum_{k=1}^{m} \min\,[x_k, y_k]}{\sum_{k=1}^{m} \max\,[x_k, y_k]} \quad . \tag{S.6}$$

For firm i we define the binary input vector, π_i^{in}, as $\pi_{ik}^{\text{in}} = 1$ if $\bar{\Pi}_{ik}^{\text{in}} > 0$ and the binary output vector, π_i^{out}, as $\pi_{ik}^{\text{out}} = 1$ if $\bar{\Pi}_{ik}^{\text{out}} > 0$. Analogously to the IOC and OOC we define the pairwise input vector Jaccard Index, IJI, and the pairwise output vector Jaccard Index, OJI, of two firms i and j as

$$\text{IJI}_{ij} = \frac{\sum_{k=1}^{m} \min\left[\pi_{ik}^{\text{in}},\ \pi_{jk}^{\text{in}}\right]}{\sum_{k=1}^{m} \max\left[\pi_{ik}^{\text{in}},\ \pi_{jk}^{\text{in}}\right]} \quad , \qquad \text{OJI}_{ij} = \frac{\sum_{k=1}^{m} \min\left[\pi_{ik}^{\text{out}},\ \pi_{jk}^{\text{out}}\right]}{\sum_{k=1}^{m} \max\left[\pi_{ik}^{\text{out}},\ \pi_{jk}^{\text{out}}\right]} \quad . \tag{S.7}$$

Results for the Jaccard Index

We show that the results from the main text do not depend on the specific similarity measure. We show the results of Fig. 2 and Fig. 3 are qualitatively similar when using IJI and OJI instead of IOC and OOC.

First, we show the pairwise similarity distributions of input and output vectors for firms of NACE class 26, Manufacture of computer, electronic and optical products measured with the Jaccard index. SI Fig. S8a-d show input Jaccard Indices, IJI_{ij}, and SI Fig. S8e-h output Jaccard Indices, OJI_{ij}, visualized as histograms, for four in-degree, k_i^{in}, (number of suppliers) and out-degree, k_i^{out} (number of buyers), bins, respectively. Jaccard Index values are on the x-axis in bins of width 0.05; the y-axis shows the frequency to fall in the respective bin. Vertical solid lines correspond to median and dashed lines to mean overlap coefficients. SI Fig. S8a-d shows that the median and mean similarities of input vectors measured by the IJI are slightly higher than for the IOC. The medians are 0, 0.2, 0.231 and 0.425 for the IJI and 0, 0.121, 0.199, and 0.343 for the IOC. The differences in means is slightly smaller. Further, the standard deviation is smaller for the IJI values than for the IOC values (0.261, 0.109, 0.091, 0.119,

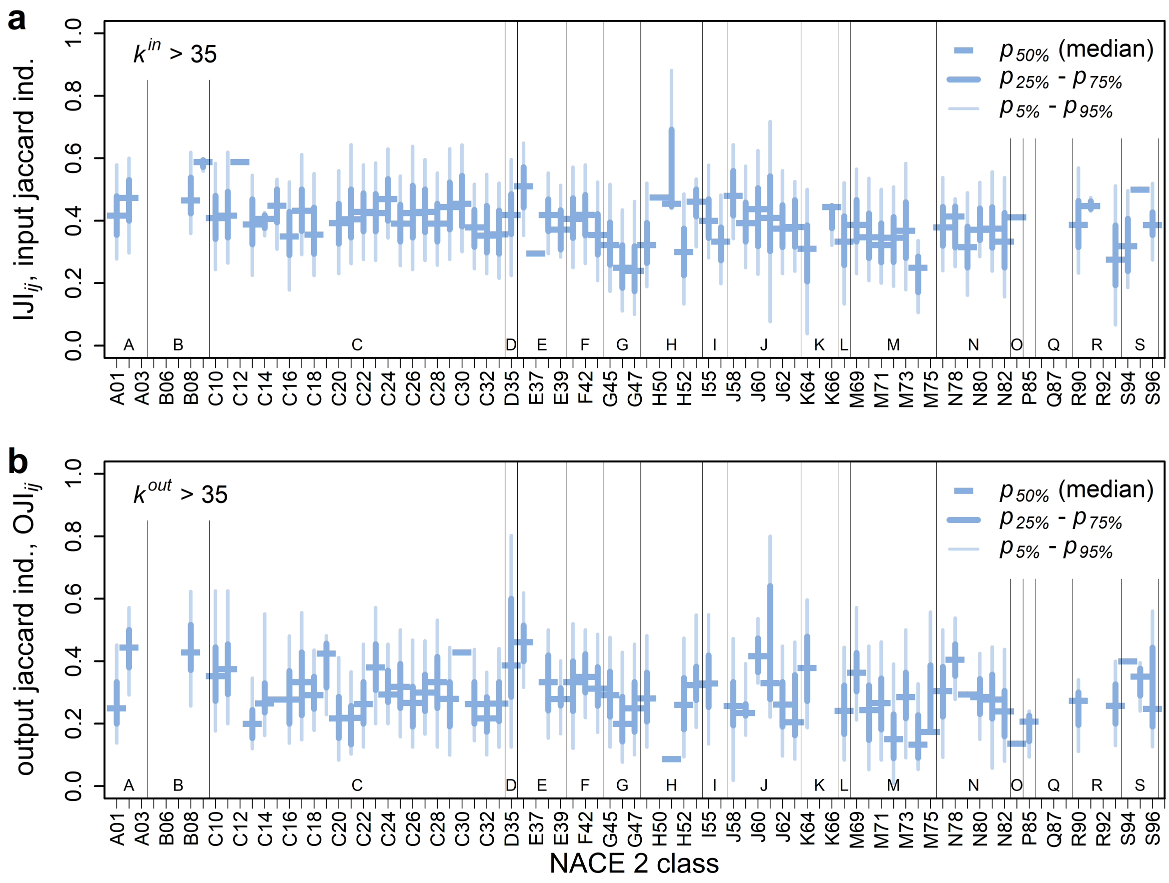

Figure S9. Pairwise similarity distributions of input- and output-vectors of firms within each NACE2 industry. Similarity is measured with the Jaccard Index for firms with more than 35 suppliers (a) and buyers (b), respectively. The y-axis denotes the Jaccard Index, the x-axis shows the NACE2 code for the respective boxplots. The dark blue horizontal bars correspond to the median, ($p_{50\%}$), dark blue vertical lines to the interquartile range ($p_{25\%} - p_{75\%}$), and thin light blue vertical lines to error bars ($p_{5\%} - p_{95\%}$). Thin black vertical lines separate NACE1 classes. Empty columns indicate sectors with less than two firms in this degree bin. a) distributions of pairwise intra-industry input Jaccard Index, IJI_{ij}. The average of the mean (median) input Jaccard Index, across NACE2 industries is 0.398 (0.394) and the standard deviation of mean (median) input Jaccard Index is 0.07 (0.067). The average standard deviation is 0.031. This indicates that relatively low input similarity is the norm with few outliers. b) distributions of pairwise intra-industry output vector Jaccard Index, OOC_{ij}. The average of the mean (median) output overlaps, across NACE2 industries is 0.301 (0.291) and the standard deviation of mean (median) output Jaccard Index values is 0.076 (0.077), indicating that relatively low output similarities are the norm with few outliers. The average standard deviation is 0.031. Output overlaps are on average lower than the input overlaps, but there is only slightly more variation across industries. If industry-level aggregation were fully representative for the IO-vectors of firms in both panels all distributions would correspond to a single bar at the value 1.

vs. 0.282, 0.192, 0.161, 0.148). For smaller size bins the IJI distribution is also right skewed, but less so and the distribution becomes symmetric faster than for the IOC. As indicated by the lower standard deviations the distributions are narrower. In general the similarities are relatively low and far away from the value of one, which would indicate that industry-level aggregation is representative for firm-level input vectors.

Fig. S8e-h shows that the median and mean similarities of output vectors measured by the OJI are slightly higher than for the OOC. The medians are 0, 0.1, 0.2 and 0.267 for the OJI and 0, 0.025, 0.119, and 0.119 for the OOC. The differences in the means are smaller. Further, the standard deviation is smaller for the OJI values than for the OOC values (0.163, 0.109, 0.127, 0.106, vs. 0.190, 0.141, 0.156, 0.123). For smaller size bins the OJI distribution is also right skewed, but less so and the distribution becomes symmetric faster. As indicated by the lower standard deviations the distributions are narrower. In general the similarities are relatively low and far away from the value of one, which would indicate that industry-level aggregation is representative for firm-level input vectors.

The patterns of increasing similarity with degree also holds true for IJI and OJI. SI Fig. S9 shows the IJI and OJI for the degree bins of firms with more than 35 suppliers ($k^{\mathrm{in}} > 35$) and more than 35 customers ($k^{\mathrm{out}} > 35$), respectively. SI Fig. S9a shows that as for NACE class C26 the average similarity is slightly higher for the IJI than for the IOC. For the IJI_{ij} the average of the mean (median) input overlaps, across NACE2 industries is 0.398 (0.394) and the standard deviation of mean (median) input overlaps is 0.07 (0.067). For the IOC_{ij} the average of the mean (median) input overlaps, across NACE2 industries is 0.35 (0.33) and the standard deviation of mean (median) input overlaps is 0.084 (0.102). The average standard deviation for IJI is 0.031, which is substantially lower than the average standard deviation for the IOC of 0.156. This implies that the distributions are

on average more concentrated for the jaccard index based input vector similarity. This is not surprising as both measures have a similar numerator, but the binary counting of input vectors in the Jaccard Index probably reduces the range of possible lower range outliers. This is because the binary counting of the JI tends to give overlaps that are small when measured with the OC a higher weight (the JI denominator divides in the best case by the number of joint inputs and in the worst case by the number of different inputs of both firms added up). The same reasoning could explain the slightly higher average similarity values of JI over OC. SI Fig. S9b shows the results for the pairwise output vector similarity based on the Jaccard Index. For the OJI_{ij} the average of the mean (median) output vector Jaccard Index, across NACE2 industries is 0.301 (0.291) and the standard deviation of mean (median) input Jaccard Index is 0.076 (0.077). OOC_{ij}. The average of the mean (median) output overlaps, across NACE2 industries is 0.282 (0.257) and the standard deviation of mean (median) output overlaps is 0.147 (0.161). Again the average Jaccard Index based similarity, OJI, is slightly higher than the average output overlap coefficient OOC. The average standard deviation for OJI is 0.031, which is substantially lower than the average standard deviation for the OOC of 0.17. This implies that the distributions are on average more concentrated for the Jaccard Index based output vector similarity.

Overall we observe a qualitatively similar degree of similarity when using the Jaccard Index instead of the overlap coefficient.

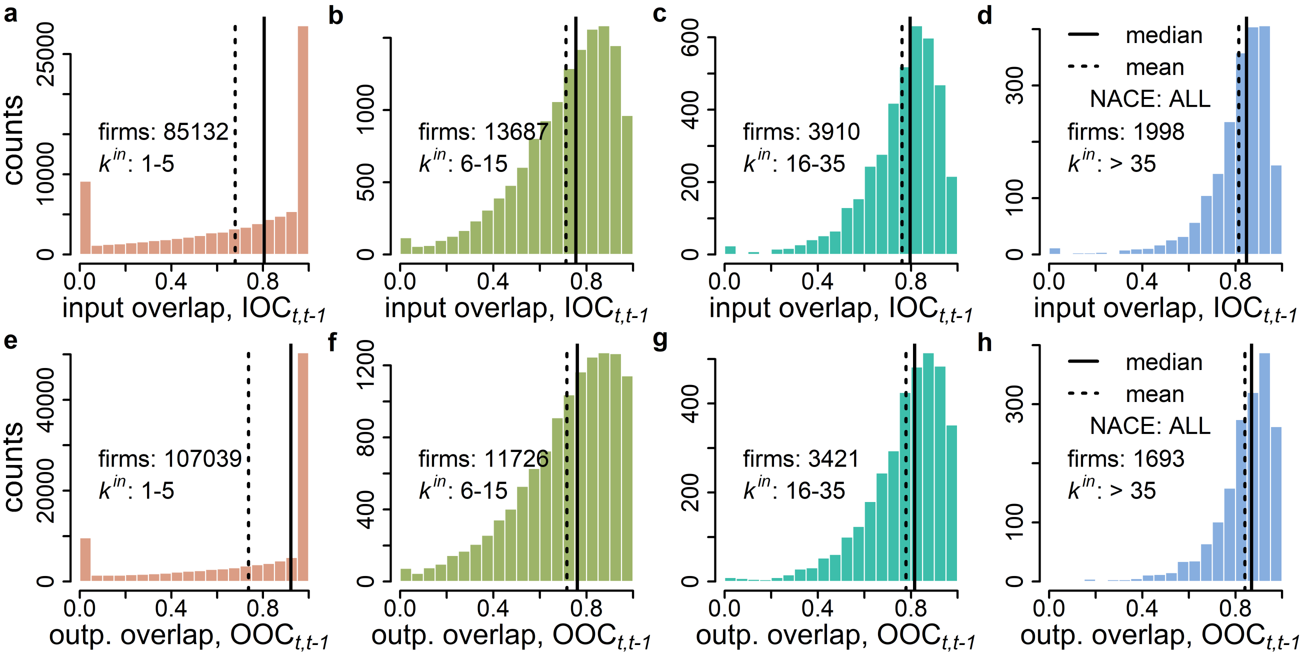

Figure S10. Distribution of input and output overlap coefficients of firms' input- and output-vectors across the years 2019 and 2018 over all NACE2 industries. The overlap coefficients, OC, are on the x-axis and counts for the respective OC-value bin on the y-axis. a-d) illustrate the distributions of, $\text{IOC}_{t,t-1}$, across all NACE 2 industries for the four in-degree bins (1-5, 6-15, 16-35, >35) as histograms. The median and mean IOCs over time, $\text{IOC}_{t,t-1}$, are 0.805 (0.678) , 0.755 (0.712), 0.797 (0.761) and 0.847 (0.814), respectively, indicated by the vertical solid (dashed) lines. The standard deviations for the in-degree bins are 0.345, 0.203, 0.161 and 0.142, respectively, and decreasing with the number of in-links. e-h) illustrate the distributions of, $\text{OOC}_{t,t-1}$, for the respective out-degree bins. The median and (mean) OOCs over time, $\text{OOC}_{t,t-1}$, are 0.922 (0.737) , 0.816 (0.778), 0.869 (0.841) and 0.847 (0.814), The standard deviations for the out-degree bins are 0.34, 0.209, 0.163 and 0.128; again decreasing with the in-link number. The similarity of firms input- and output-vectors over time is substantially higher than for the pairwise intra-industry similarities.

SI Section 7. Input and output vectors are similar over time

In this section we show that the low pairwise IOC and OOC values for firms within the same industries are not a generic feature of the micro-level data. The similarity of firms input and output vectors over time is substantially higher than the intra-industry similarities. To show this we calculate for each firm the overlap coefficient of its relative input vector in the year t with its input vector in the previous year $t-1$ as

$$\text{IOC}_{t,t-1} = \sum_{k=1}^{m} \min\left[\bar{\Pi}_{ik}^{\text{in}}(t),\ \bar{\Pi}_{ik}^{\text{in}}(t-1)\right] \tag{S.8}$$

Analgously, we compute the output overlap coefficient between two years t and $t-1$ as

$$\text{OOC}_{t,t-1} = \sum_{k=1}^{m} \min\left[\bar{\Pi}_{ik}^{\text{out}}(t),\ \bar{\Pi}_{ik}^{\text{out}}(t-1)\right] \tag{S.9}$$

The two measures indicate the fraction of total inputs (outputs) that is spent on (sold to) the same industry in the two year. We calculate the overlap coefficients over time for the years 2019 and 2018. Firms are allocated into the respective in- and out-degree bins based on their number of suppliers or customers in the year 2018.

SI Fig. S10 we show the distribution of input and output overlap coefficients of firms' input- and output-vectors across the years 2019 and 2018 over all NACE2 industries. The overlap coefficients, OC, are on the x-axis and counts for the respective OC-value bin on the y-axis. SI Fig. S10a-d illustrates the distributions of $\text{IOC}_{t,t-1}$ across all NACE 2 industries for the four in-degree bins (1-5, 6-15, 16-35, >35) as histograms. The median and mean IOCs over time, $\text{IOC}_{t,t-1}$, are 0.805 (0.678) , 0.755 (0.712), 0.797 (0.761) and 0.847 (0.814), respectively, and thus substantially higher than for the intra-industry IOCs. The standard deviations for the in-degree bins are 0.345, 0.203, 0.161 and 0.142, respectively and decreasing with the number of in-links. The distributions are left skewed, i.e. very low overlap coefficients are outliers and for the smallest in-degree bin bi-modal. In all four bins there are firms having almost zero input overlap in the two years. While this number is relatively high for the smallest in-degree bin it decreases strongly for higher in-degree bins. For firms with few suppliers this is most likely due to the change of a single or the primary supplier. For the few cases where firms with many suppliers have almost no overlap the likely explanation is that they went out of business between the two years and did not source inputs anymore in the second year. As the network is growing — due to a reduction of the link reporting threshold in mid-2018 — the overlaps over time shown here might be smaller than in practice. Therefore, we check also the probability of retaining an input type from the year 2018 in the year 2019 and find that these are even higher than the overlap coefficients, for details see SI Fig. S11a-d.

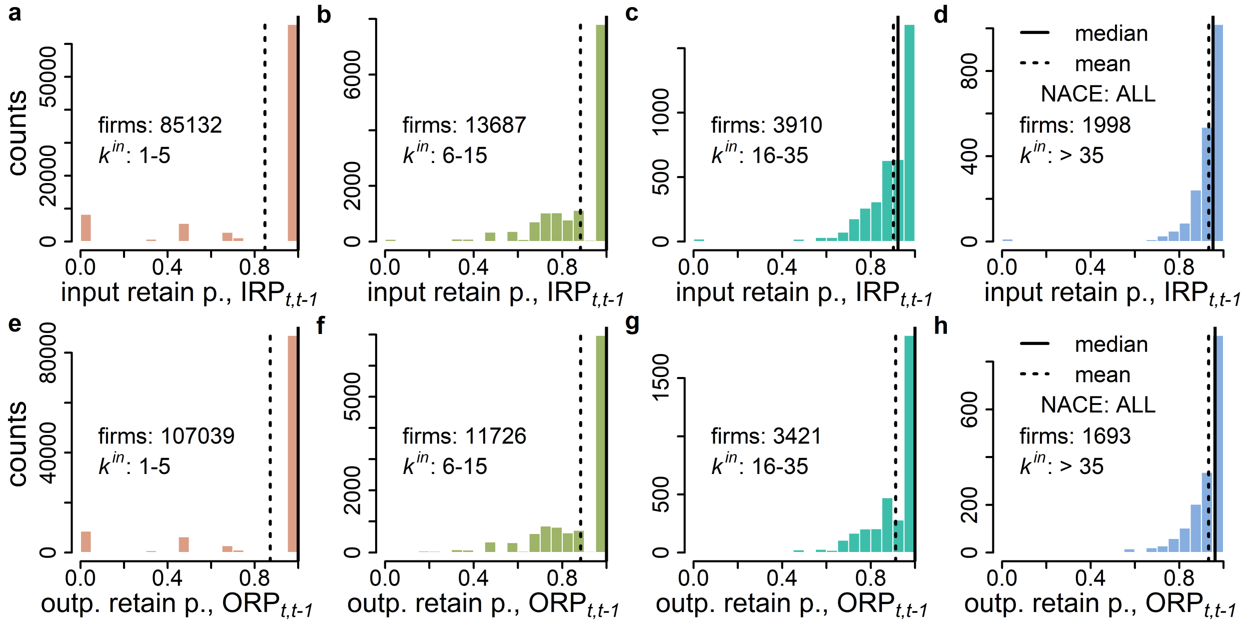

Figure S11. Distribution of input and output retention probabilities (IRPs and ORPs) of firms for 2019 and 2018 across all industries. The retention probabilities, RPs, are on the x-axis and counts for the respective RP-value bins on the y-axis. a-d) illustrate the distributions of, $\text{IRP}_{t,t-1}$, across all NACE 2 industries for the four in-degree bins (1-5, 6-15, 16-35, >35) as histograms. The median and mean IRPs over time, $\text{IRP}_{t,t-1}$, are 1 (0.847) , 1 (0.882), 0.923 (0.902) and 0.952 (0.933), respectively, indicated by the vertical solid (dashed) lines. The means increase with the in-degree. The standard deviations for the in-degree bins are 0.316, 0.173, 0.133 and 0.108, respectively, and decreasing with the number of in-links. With increasing in-degree the distributions become more concentrated on the value 1, i.e. most firms retain almost all NACE2 input types. e-h) illustrate the distributions of, $\text{ORP}_{t,t-1}$, for the respective out-degree bins. The median and (mean) ORPs over time, $\text{ORP}_{t,t-1}$, are 1 (0.872) , 1 (0.883), 1 (0.913) and 0.962 (0.933), i.e. means increase with in-degree. The standard deviations for the out-degree bins are 0.295, 0.177, 0.130 and 0.100; again decreasing with the in-link number. With increasing out-degree the distributions become more concentrated on the value 1, i.e. most firms retain almost all NACE2 customer industries. The similarity of firms input- and output-vectors over time is slightly higher than the intra-industry similarities.

Analogously Fig. S10e-h illustrates the distributions of $\text{OOC}_{t,t-1}$ for the respective out-degree bins. The median and (mean) OOCs over time, $\text{OOC}_{t,t-1}$, are 0.922 (0.737) , 0.816 (0.778), 0.869 (0.841) and 0.847 (0.814), respectively, and thus substantially higher than for the intra-industry OOCs and slightly higher than the IOCs over time. The standard deviations for the out-degree bins are 0.34, 0.209, 0.163 and 0.128; again decreasing with the number of out-links. The distributions are left skewed and for the smallest out-degree bin bi-modal. In all four bins there are firms having almost zero output overlap in the two years, but substantially less so than for the $\text{IOC}_{t,t-1}$. The probability of retaining an output type (buyer industry) from the year 2018 in the year 2019 is again higher than the overlap coefficients, for details see SI Fig. S11a-d.

To show that firms overwhelmingly keep existing inputs and buyer industries we calculate for each firm the input retention probability and the output retention probability from the binary input and output vectors of a year t with the previous year $t-1$. Recall the binary input vector, π_i^{in}, is defined as $\pi_{ik}^{\text{in}} = 1$ if $\bar{\Pi}_{ik}^{\text{in}} > 0$ and the binary output vector, π_i^{out}, as $\pi_{ik}^{\text{out}} = 1$ if $\bar{\Pi}_{ik}^{\text{out}} > 0$.

We define the input retention probability, $\text{IRP}_{t,t-1}$, for a firm i between two years t and $t-1$ as

$$\text{IRP}_{t,t-1} = \frac{\sum_{k=1}^{m} \min\left[\pi_{ik}^{\text{in}}(t),\ \pi_{ik}^{\text{in}}(t-1)\right]}{\sum_{k=1}^{m} \pi_{ik}^{\text{in}}(t-1)} \quad . \tag{S.10}$$

$\text{IRP}_{t,t-1}$ is the probability that a random input contained in the input vector of firm i in year $t-1$ is still present in the input vector of firm i at time t. Analogously, we compute the output retention probability, $\text{ORP}_{t,t-1}$, for a firm i between two years t and $t-1$ as

$$\text{ORP}_{t,t-1} = \frac{\sum_{k=1}^{m} \min\left[\pi_{ik}^{\text{out}}(t),\ \pi_{ik}^{\text{out}}(t-1)\right]}{\sum_{k=1}^{m} \min \pi_{ik}^{\text{out}}(t-1)} \quad . \tag{S.11}$$

$\text{ORP}_{t,t-1}$ is the probability that a random buyer industry contained in the output vector vector of firm i in year $t-1$ is still present in the output vector of firm i at time t.

We calculate IRP and ORP over time for the years 2019 and 2018 for each firm. Firms are allocated into the respective in- and out-degree bins based on their number of suppliers or customers in the year 2018. The results are shown as histograms SI Fig. S11, where the retention probabilities are on the x-axis and counts for the respective RP-value bins on the y-axis. SI Fig. S11a-d illustrate the distributions of, $\text{IRP}_{t,t-1}$, across all NACE 2 industries for the four in-degree bins (1-5, 6-15, 16-35, >35) as histograms. The median and mean IRPs over time, $\text{IRP}_{t,t-1}$, are 1 (0.847) , 1 (0.882), 0.923 (0.902) and 0.952 (0.933), respectively, indicated by the vertical solid (dashed) lines. The means increase with the in-degree. The standard deviations for the in-degree bins are 0.316, 0.173, 0.133 and 0.108, respectively, and decreasing with the number of in-links. With increasing in-degree the distributions

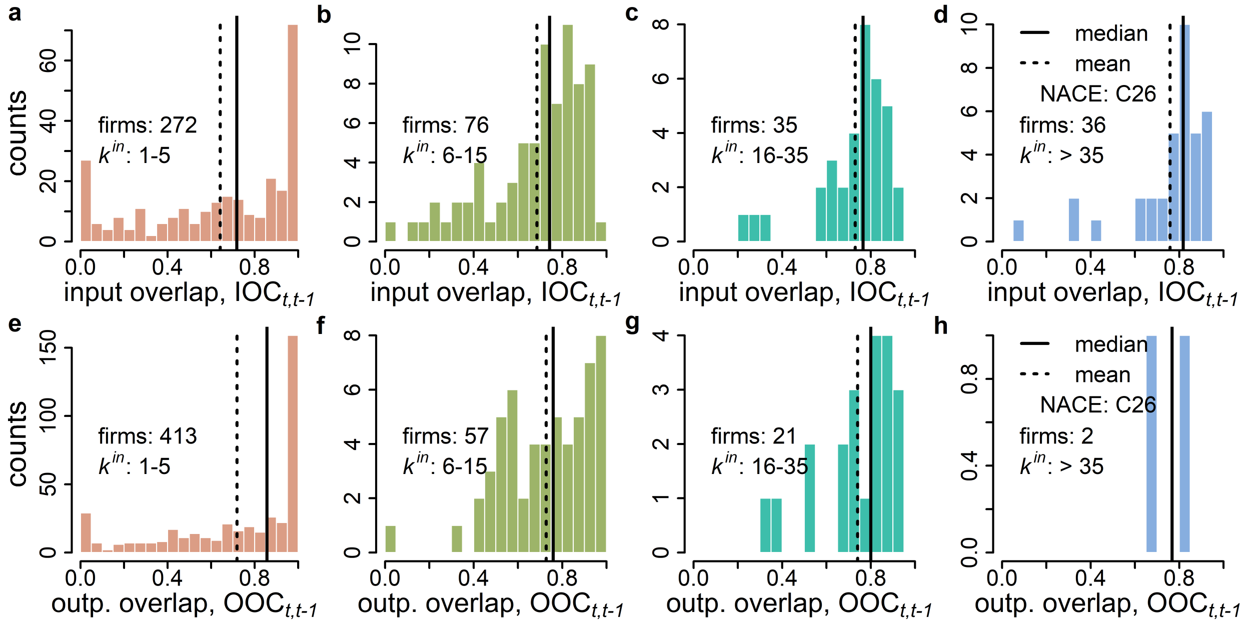

Figure S12. Similarity of firms' input- and output-vectors for 2019 and 2018 for NACE2 class C26, measures with the overlap coefficient (OC). The OC is on the x-axis and the counts for the respective OC-value bin on the y-axis. a-d) illustrate the distributions of, $IOC_{t,t-1}$, across all NACE 2 industries for the four in-degree bins (1-5, 6-15, 16-35, >35) as histograms. The median and mean IOCs over time, $IOC_{t,t-1}$, are 0.718 (0.642) , 0.743 (0.686), 0.765 (0.730) and 0.818 (0.759), respectively, indicated by the vertical solid (dashed) lines, and increasing with in-degree. The standard deviations for the in-degree bins are 0.34, 0.217, 0.167 and 0.188, respectively, and decreasing with the number of in-links. e-h) illustrate the distributions of, $OOC_{t,t-1}$, for the respective out-degree bins. The median and (mean) OOCs over time, $OOC_{t,t-1}$, are 0.857 (0.719) , 0.759 (0.727), 0.801 (0.740) and 0.768 (0.768). Only the means are increasing, but not the medians. The standard deviations for the out-degree bins are 0.321, 0.206, 0.176 and 0.115; again decreasing with the out-link number. The similarity of firms input- and output-vectors over time is substantially higher than the intra-industry similarities.

become more concentrated on the value 1, i.e. most firms retain almost all NACE2 input types. SI Fig. S11e-h illustrate the distributions of, $ORP_{t,t-1}$, for the respective out-degree bins. The median and (mean) ORPs over time, $ORP_{t,t-1}$, are 1 (0.872) , 1 (0.883), 1 (0.913) and 0.962 (0.933). The means increase with the out-degree. The standard deviations for the out-degree bins are 0.295, 0.177, 0.130 and 0.100; again decreasing with the out-link number. With increasing out-degree the distributions become more concentrated on the value 1, i.e. most firms retain almost all NACE2 customer industries. The similarity of firms input- and output-vectors over time is substantially higher than the intra-industry similarities.

Overlaps over time for industries

In this section we show the distribution of input and output overlap coefficients over time for specific NACE2 industries. For completeness we illustrate the similarity over time for NACE2 industry C26 in SI Fig. S12. The overlap coefficient, OC, is on the x-axis and counts for the respective OC-value bin on the y-axis. SI Fig. S12a-d illustrate the distributions of, $IOC_{t,t-1}$, across all NACE 2 industries for the four in-degree bins (1-5, 6-15, 16-35, >35) as histograms. The median and mean IOCs over time, $IOC_{t,t-1}$, are 0.718 (0.642) , 0.743 (0.686), 0.765 (0.730) and 0.818 (0.759), respectively, indicated by the vertical solid (dashed) lines, and increasing with in-degree The standard deviations for the in-degree bins are 0.34, 0.217, 0.167 and 0.188, respectively, and decreasing with the number of in-links. SI Fig. S12e-h illustrate the distributions of, $OOC_{t,t-1}$, for the respective out-degree bins. The median and (mean) OOCs over time, $OOC_{t,t-1}$, are 0.857 (0.719) , 0.759 (0.727), 0.801 (0.740) and 0.768 (0.768). Only the means are increasing, but not the medians. The standard deviations for the out-degree bins are 0.321, 0.206, 0.176 and 0.115; again decreasing with the in-link number. The similarity of firms input- and output-vectors over time is substantially higher, than the intra-industry similarities. For NACE C26 neither input or output overlaps are consistently larger across degree bins.

Next we look at the distributions of $IOC_{t,t-1}$ and $OOC_{t,t-1}$ across NACE2 industries. For the following figures, the y-axis denotes the overlap coefficients between the two years, the x-axis shows the NACE2 code for the respective boxplots. The dark horizontal bars correspond to the median, ($p_{50\%}$), dark vertical lines to the interquartile range ($p_{25\%} - p_{75\%}$), and thin light vertical lines to error bars ($p_{5\%} - p_{95\%}$). Thin black vertical lines separate NACE1 classes. Empty columns indicate sectors with less than two firms in this degree bin. First, we focus on the distributions of input overlaps for the years 2019 and 2018, $IOC_{t,t-1}$, in SI Fig. S13a and SI Fig. S14. SI Fig. S13a shows the distributions of firms input overlap coefficients, $IOC_{t,t-1}$, for firms with more than 35 suppliers, $k_i^{in} > 35$. The average of the mean (median) input overlaps, across NACE2 industries is 0.784 (807) and the standard deviation of mean (median) input overlaps is 0.099 (0.102). The average standard deviation is 0.124. This indicates that high input overlaps are the norm with few outliers. SI Fig. S14a shows the distributions of input overlap coefficients, $IOC_{t,t-1}$, for firms with in-degree between one and five, $1 \leq k_i^{in} \leq 5$. The mean over the industries' mean (median) $IOC_{t,t-1}$ is 0.660 (0.763), the standard deviation of mean (median) IOCs is 0.079 (0.104). The mean standard deviation is 0.334. SI Fig. S14b shows the distributions of input

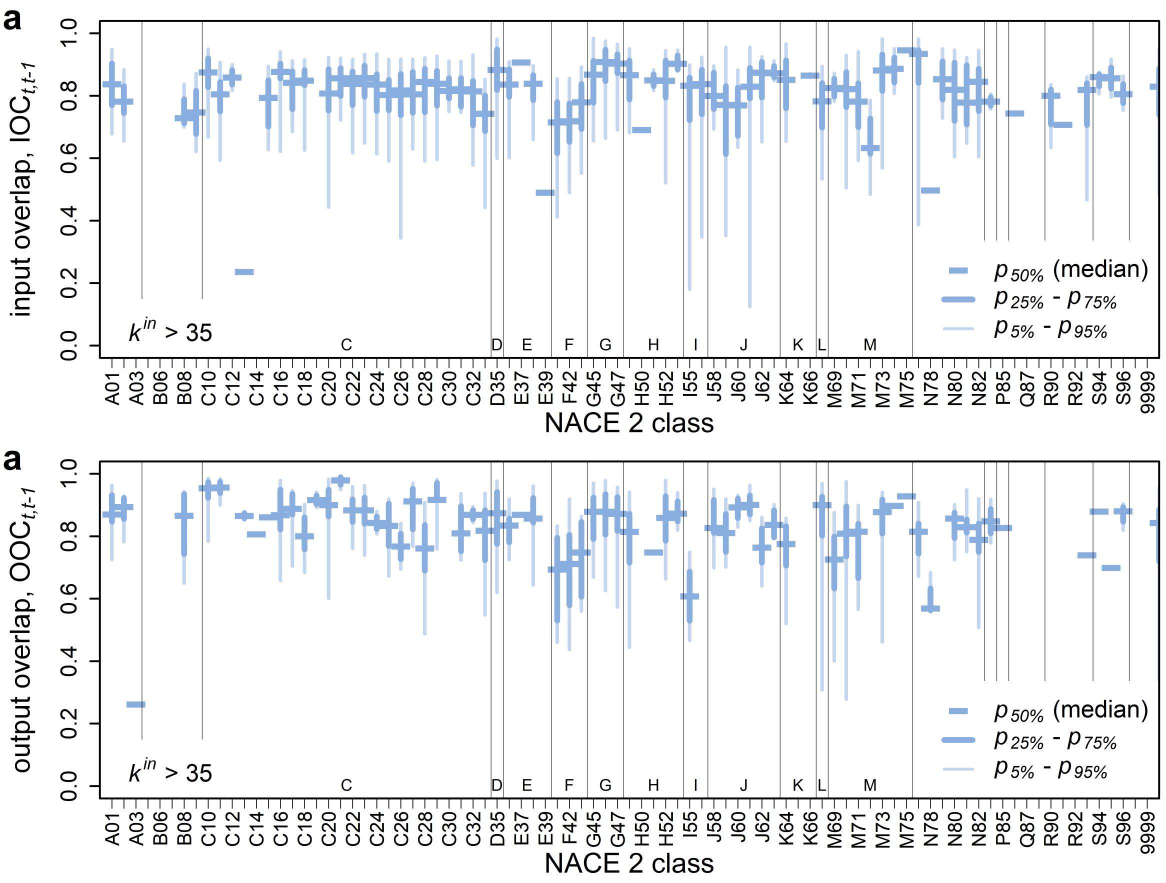

Figure S13. Similarity distributions of input- and output-vectors of firms between 2019 and 2018 for each NACE2 industry. Similarity is measured with the overlap coefficient for firms with more than 35 suppliers (a) and buyers (b), respectively. The y-axis denotes the overlap coefficients between the two years, the x-axis shows the NACE2 code for the respective boxplots. The dark blue horizontal bars correspond to the median, ($p_{50\%}$), dark blue vertical lines to the interquartile range ($p_{25\%} - p_{75\%}$), and thin light blue vertical lines to error bars ($p_{5\%} - p_{95\%}$). Thin black vertical lines separate NACE1 classes. Empty columns indicate no firms in this degree bin. a) distributions of firms input overlap coefficients, $\text{IOC}_{t,t-1}$. The average of the mean (median) input overlaps, across NACE2 industries is 0.784 (807) and the standard deviation of mean (median) input overlaps is 0.099 (0.102). The average standard deviation is 0.124. This indicates that high input overlaps are the norm with few outliers. b) distributions of pairwise intra-industry output overlap coefficients, $\text{OOC}_{t,t-1}$. The average of the mean (median) output overlaps, across NACE2 industries is 0.813 (0.828) and the standard deviation of mean (median) output overlaps is 0.101 (0.103), indicating that relatively low output overlaps are the norm with few outliers. The average standard deviation is 0.108. Output overlaps are on average slightly higher than input overlaps.

overlap coefficients, $\text{IOC}_{t,t-1}$, for firms with in-degree between 6 and fifteen, $6 \leq k_i^{in} \leq 15$. The mean over the industries' mean (median) $\text{IOC}_{t,t-1}$ is 0.692 (0.729), the standard deviation of mean (median) IOCs is 0.097 (0.100). The mean standard deviation is 0.184. SI Fig. S14c shows the distributions of input overlap coefficients, $\text{IOC}_{t,t-1}$, for firms with in-degree between 16 and 35, $16 \leq k_i^{in} \leq 35$. The mean over the industries' mean (median) $\text{IOC}_{t,t-1}$ is 0.730 (0.094), the standard deviation of mean (median) IOCs is 0.094 (0.096). The mean standard deviation is 0.162. Second, we focus on the distributions of output overlaps for the years 2019 and 2018, $\text{OOC}_{t,t-1}$, in SI Fig. S13b and SI Fig. S15. SI Fig. S13b shows the distributions of output overlap coefficients, $\text{OOC}_{t,t-1}$, for firms with more than 35 customers, $k_i^{out} > 35$. The average of the mean (median) output overlaps, across NACE2 industries is 0.813 (0.828) and the standard deviation of mean (median) output overlaps is 0.101 (0.103), indicating that relatively low output overlaps are the norm with few outliers. The average standard deviation is 0.108. Output overlaps are on average slightly higher, than input overlaps for the degree bin >35. SI Fig. S15a shows the distributions of output overlap coefficients, $\text{OOC}_{t,t-1}$, for firms with out-degree between one and five, $1 \leq k_i^{out} \leq 5$. The mean over the industries' mean (median) $\text{OOC}_{t,t-1}$ is 0.727 (0.876), the standard deviation of mean (median) OOCs is 0.085 (0.110). The mean standard deviation is 0.331. SI Fig. S15b shows the distributions of output overlap coefficients, $\text{OOC}_{t,t-1}$, for firms with out-degree between 6 and 15, $6 \leq k_i^{out} \leq 15$. The mean over the industries' mean (median) $\text{OOC}_{t,t-1}$ is 0.714 (0.749), the standard deviation of mean (median) OOCs is 0.084 (0.093). The mean standard deviation is 0.208. SI Fig. S15c shows the distributions of output overlap coefficients, $\text{OOC}_{t,t-1}$, for firms with out-degree between 16 and 35, $16 \leq k_i^{out} \leq 35$. The mean over the industries' mean (median) $\text{OOC}_{t,t-1}$ is 0.760 (0.780), the standard deviation of mean (median) OOCs is 0.119 (0.120). The mean standard deviation is 0.139. Overall overlap coefficients of firms input- and output vectors for the years 2018 and 2019 are substantially higher than the pairwise overlap coefficients within industries.

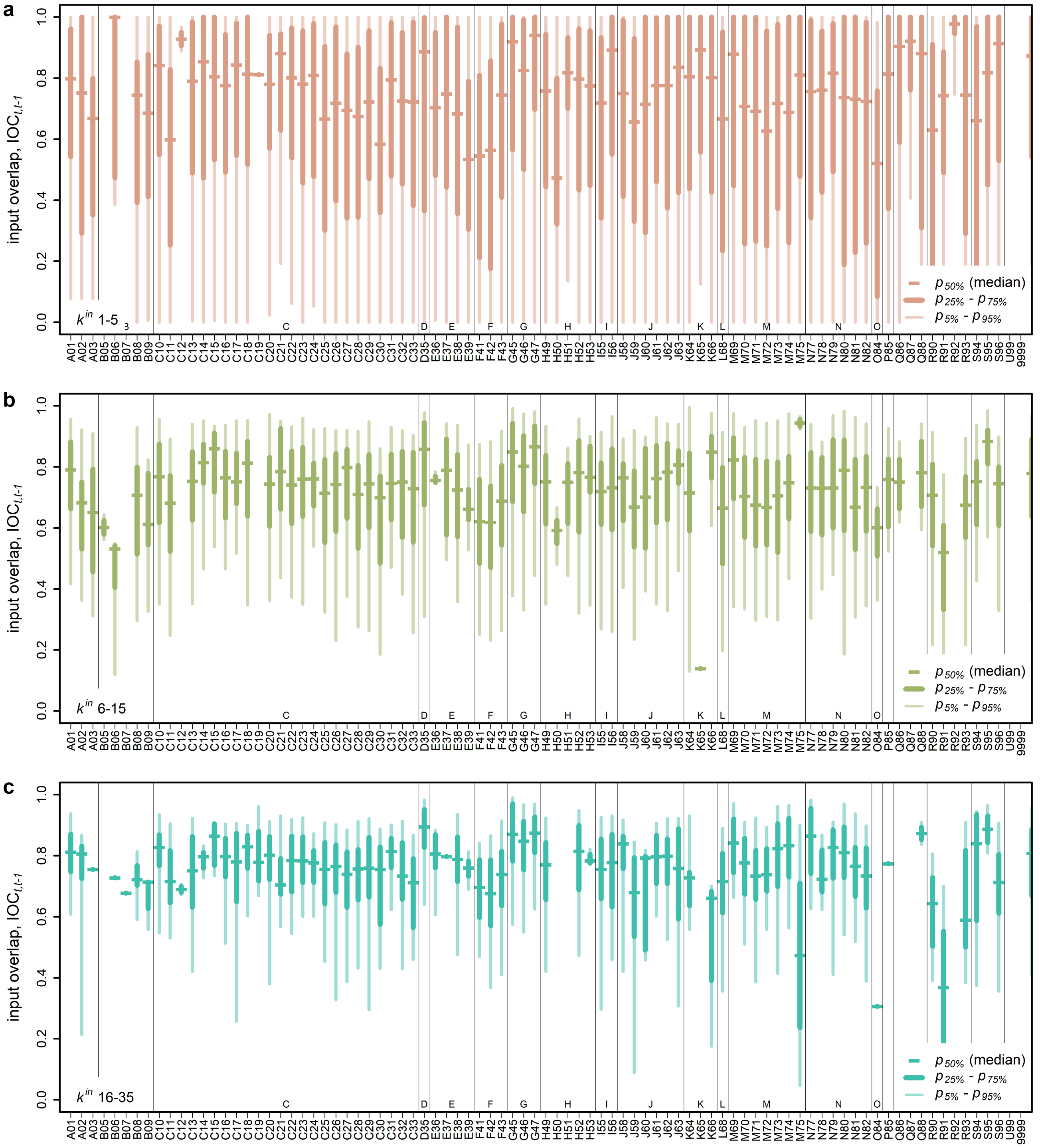

Figure S14. Distributions of input vector overlaps, $\mathrm{IOC}_{t,t-1}$, of firms across NACE2 industries for the years 2019 and 2018. NACE2 classes are on the x-axis; overlap coefficients on the y-axis. a) distributions of input overlap coefficients, $\mathrm{IOC}_{t,t-1}$, for firms with in-degree between one and five, $1 \le k_i^{in} \le 5$. The mean over the industries' mean (median) $\mathrm{IOC}_{t,t-1}$ is 0.660 (0.763), the standard deviation of mean (median) IOCs is 0.079 (0.104). The mean standard deviation is 0.334. b) distributions of input overlap coefficients, $\mathrm{IOC}_{t,t-1}$, for firms with in-degree between 6 and fifteen, $6 \le k_i^{in} \le 15$. The mean over the industries' mean (median) $\mathrm{IOC}_{t,t-1}$ is 0.692 (0.729), the standard deviation of mean (median) IOCs is 0.097 (0.100). The mean standard deviation is 0.184. c) distributions of input overlap coefficients, $\mathrm{IOC}_{t,t-1}$, for firms with in-degree between 16 and 35, $16 \le k_i^{in} \le 35$. The mean over the industries' mean (median) $\mathrm{IOC}_{t,t-1}$ is 0.730 (0.094), the standard deviation of mean (median) IOCs is 0.094 (0.096). The mean standard deviation is 0.162.

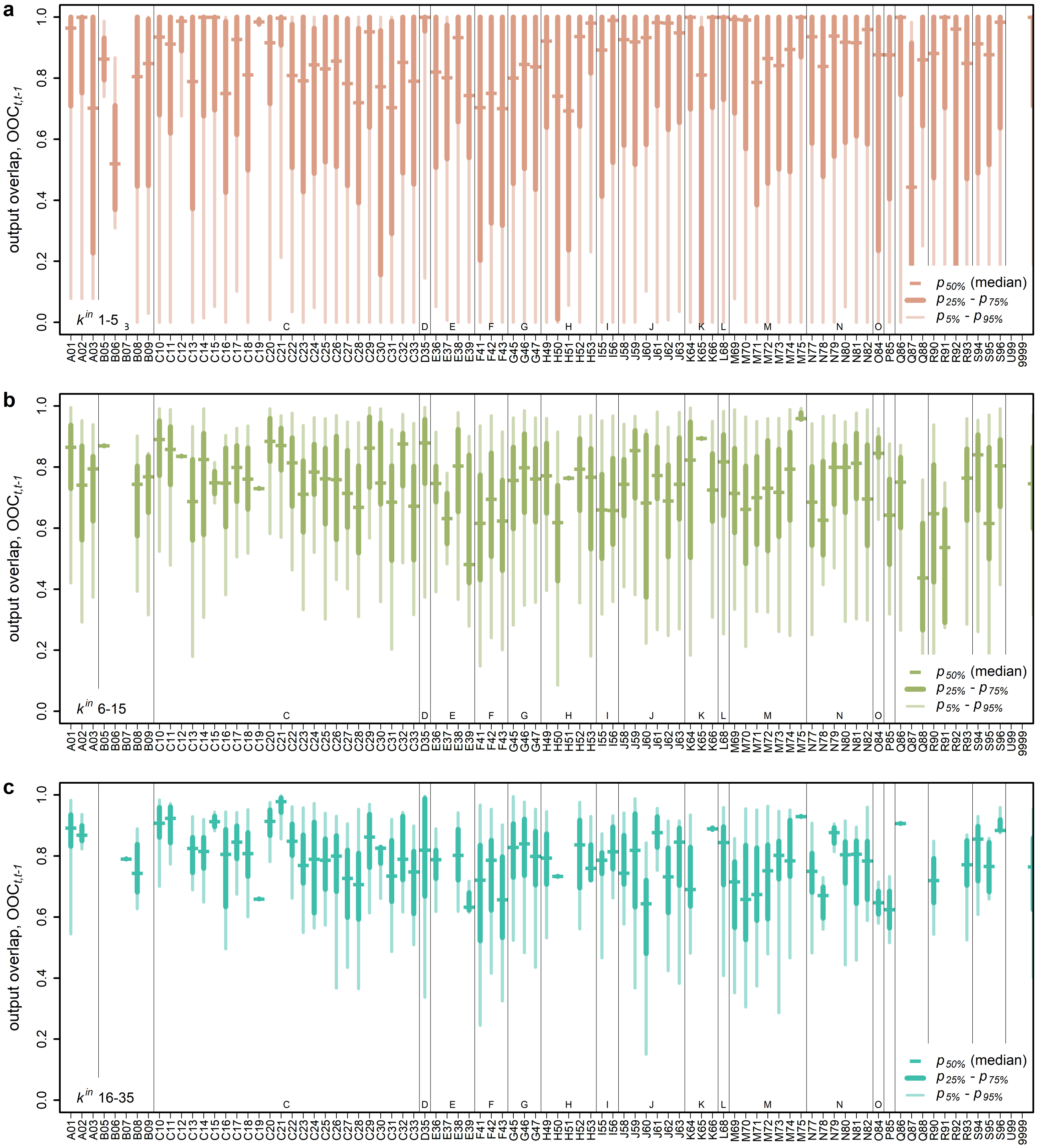

Figure S15. Distributions of output vector overlaps, $OOC_{t,t-1}$, of firms across NACE 2 industries for the years 2019 and 2018. NACE2 classes are on the x-axis; overlap coefficients on the y-axis. a) distributions of output overlap coefficients, $OOC_{t,t-1}$, for firms with out-degree between one and five, $1 \leq k_i^{out} \leq 5$. The mean over the industries' mean (median) $OOC_{t,t-1}$ is 0.727 (0.876), the standard deviation of mean (median) OOCs is 0.085 (0.110). The mean standard deviation is 0.331. b) distributions of output overlap coefficients, $OOC_{t,t-1}$, for firms with out-degree between 6 and 15, $6 \leq k_i^{out} \leq 15$. The mean over the industries' mean (median) $OOC_{t,t-1}$ is 0.714 (0.749), the standard deviation of mean (median) OOCs is 0.084 (0.093). The mean standard deviation is 0.208. c) distributions of output overlap coefficients, $OOC_{t,t-1}$, for firms with out-degree between 16 and 35, $16 \leq k_i^{out} \leq 35$. The mean over the industries' mean (median) $OOC_{t,t-1}$ is 0.760 (0.780), the standard deviation of mean (median) OOCs is 0.119 (0.120). The mean standard deviation is 0.139.

SI Section 8. Constructing synthetic firm-level shocks with same sector level impacts

Recall that ζ is based on the actual employment reductions in the course of the early phase of the COVID-19 pandemic. ζ_i is the reduction of the labor input for firm i between January and May 2020, $\zeta_i = \max[1 - e_i(may)/e_i(jan),\, 0]$, where e_i is the number of employees in the respective month. In this section we describe the algorithm for constructing new synthetic firm-level shock vectors, $\zeta^1, \zeta^2, \ldots, \zeta^{1,000}$, that differ in how firms within industries are affected, but are of the exactly same size when aggregated to the industry-level. From these shocks we derive the remaining production level vectors, $\Psi = \{\psi^1, \psi^2, \ldots, \psi^{1,000}\}$ that enter the shock propagation algorithm described in the Data and Methods section. We show how to construct a new shock vector, ζ^l. This problem can be solved sequentially for all industries $k \in \{1, 2 \ldots, m\}$, in our case the 593 NACE 4 classes contained in the data and the additional industry we introduce for all firms without NACE information.

We start by specifying additional industry-level notation. For a given industry $k \in \{1, 2 \ldots, m\}$ we denote the number of firms within the industry as n^k. The indices of the n^k firms in sector k are denoted as $I^k = \{i \mid p_i = k\}$. The in- and out-strength of industry k is defined as $s^{\mathrm{in},k} = \sum_{i=1}^{n} s_i^{\mathrm{in}}\, \delta_{p_i,k}$ and $s^{\mathrm{out},k} = \sum_{i=1}^{n} s_i^{\mathrm{out}}\, \delta_{p_i,k}$.

The initial shock to sector k, can be defined either through aggregating the shock vector ζ, or through aggregating the remaining production levels vector ψ, since $\psi = 1 - \zeta$. In Eq. [6] in the Data and Methods Section, we derived the shock to sector k by aggregating the vector ψ, according to firms in- and out-strengths as

$$\phi_k^{\mathrm{u}} = \frac{\sum_{i=1}^{n} \psi_i\, s_i^{\mathrm{in}}\, \delta_{p_i,k}}{\sum_{i=1}^{n} s_i^{\mathrm{in}}\, \delta_{p_i,k}}, \qquad \phi_k^{\mathrm{d}} = \frac{\sum_{i=1}^{n} \psi_i\, s_i^{\mathrm{out}}\, \delta_{p_i,k}}{\sum_{i=1}^{n} s_i^{\mathrm{out}}\, \delta_{p_i,k}} \quad . \tag{S.12}$$

ϕ_k^{u} indicates the fraction of goods sector k is still buying from its supplier industries, i.e. the fraction of k's in-strength, $s^{\mathrm{in},k}$, remaining after the shock. $\xi_k^{\mathrm{u}} = 1 - \phi_k^{\mathrm{u}}$ is the size of the corresponding demand shock that propagates upstream. ϕ_k^{d} indicates the fraction of goods sector k is still selling to its buyer industries, i.e. the fraction of k's out-strength, $s^{\mathrm{out},k}$,, remaining after the shock. $\xi_k^{\mathrm{d}} = 1 - \phi_k^{\mathrm{d}}$ is the size of the corresponding supply shock that propagates downstream. For the shock propagation algorithm it is more convenient to work with ψ, ϕ^{u} and ϕ^{d}, but for sampling new synthetic shocks we continue to work with ζ, ξ^{u} and ξ^{d}.

Our goal is to find for each firm i in industry k (i.e., where $p_i = k$) an initial shock, ζ_i^l, such that after aggregation, the sector level shock has the same size as the empirically defined original shock, ζ.

$$\begin{aligned} \text{find:} \quad & \zeta_i^l \quad \forall\, i \text{ where } p_i = k \\ \text{s.t.:} \quad & \sum_{i=1}^{n} \zeta_i^l\, s_i^{\mathrm{in}}\, \delta_{p_i,k} = \xi_k^{\mathrm{u}}\, s^{\mathrm{in},k} \quad , \\ & \sum_{i=1}^{n} \zeta_i^l\, s_i^{\mathrm{out}}\, \delta_{p_i,k} = \xi_k^{\mathrm{d}}\, s^{\mathrm{out},k} \quad , \\ & \zeta_i^l \in [0,1] \quad . \end{aligned} \tag{S.13}$$

The two right hand side terms $\xi_k^{\mathrm{u}}\, s^{\mathrm{in},k}$ and $\xi_k^{\mathrm{d}}\, s^{\mathrm{out},k}$ are the target shock sizes the new firm-level shock, ζ^l, needs to fulfil for industry k. We know that at least one solution exists always exists, the original initial shock ζ. Here, our sampled shocks fulfil Eq. [S.12] at the NACE4 level, as the NACE2 level constraint would lead to even higher variability in the resulting production losses. Not that if no firm-level shock is available and we want firm-level shocks that correspond to a specific industry-level shock, then the targeted shock size can be specified directly with the sector level shock vectors, $(\xi_k^{\mathrm{u}}, \xi_k^{\mathrm{d}})$. In this way we can construct many random firm-level shocks and receive a distribution of production losses for the given industry-level shock. We solve sampling problem [S.13] in two steps.

Sampling new shocks. The first step is shown in detail in Algorithm 1. First, we define the auxiliary index set $\tilde{I}^k = \{i \mid p_i = k\}$ that contains all indices of firms belonging to sector k. The firm index, i, refers to the row and column index firm i belongs to in the adjacency matrix W, and the position of i in the industry affiliation vector p. Note we use the terms "firm i" and "index, i" interchangeably. We initialise the algorithm by setting the shock size for each firm i in industry k to zero, i.e. $\zeta_i^l \leftarrow 0$ for all $i \in I^k$. Then, we add shocks to the values, ζ_i^l $(\forall i \mid p_i = k)$, until the new shock is larger than the original shock target, i.e.,

$$\left(\sum_{i=1}^{n} \zeta_i^l\, s_i^{\mathrm{in}}\, \delta_{p_i,k}\right) \geq \left(\xi_k^{\mathrm{u}}\, s^{\mathrm{in},k}\right) \quad , \qquad \left(\sum_{i=1}^{n} \zeta_i^l\, s_i^{\mathrm{out}}\, \delta_{p_i,k}\right) \geq \left(\xi_k^{\mathrm{d}}\, s^{\mathrm{out},k}\right) \quad . \tag{S.14}$$

The shocks are added in the following way. First we draw a firm index i from the index set $\tilde{I}^k$, and delete the index i from the index set, $\tilde{I}^k$. Then we draw a shock value, $\eta \in [0,1]$, from a specified distribution that takes values between zero and 1. Here we draw the shock values from the empirical distribution of employment shocks of sector k, i.e., $\eta \sim \{\zeta_j \mid j \in I^k\}$. Note that the empirical shock distribution, $\{\zeta_j \mid j \in I^k\}$, contains only values that lie between zero and 1. Note that we could also sample more general shocks, by drawing values from, e.g., the Beta distribution that is flexible enough to sample very concentrated or very evenly distributed shocks. Note that here we do not draw negative shocks that would be interpreted as production gains, or increases in production capacity, even though this would be possible for more general shocks. We add the additional shock value, η, to the previous shock level of firm i, i.e., $\zeta_i^l \leftarrow \min[1, \zeta_i^l + e]$. The min[1, .] function is necessary, because a firm i can be drawn a second time for receiving a shock, but shocks can not be larger than one — a firm can not lose more than 100% of its production.

Since, this procedure is continued until the necessary aggregate shock level ($\xi_k^{\mathrm{u}}\, s^{\mathrm{in},k}$, $\xi_k^{\mathrm{d}}\, s^{\mathrm{in},k}$) is reached, it can happen that each firm has been drawn already and the index set $\tilde{I}^k$ is empty, i.e., $\tilde{I}^k = \emptyset$. In this case we fill up the index set again with all firms in industry k, i.e. we set $\tilde{I}^k \leftarrow \{i \,|p_i = k\}$. This can happen when in the original shock, ζ, relatively large firms received relatively large shocks, and these large firms only received small shocks in the first round of Monte Carlo draws.

Algorithm 1 Drawing shocks for firms in industry k

```
Set Ĩ^k = {i |p_i = k}                      ▷ Create the set, containing all firm indices of firms belonging to sector k.
Set ζ_i^l ← 0 for all i ∈ I^k                ▷ Initialize the algorithm by setting all shocks to zero.
while  Σ_{i=1}^n ζ_i^l s_i^in δ_{p_i,k} ≤ ξ_k^u s^{in,k}  and  Σ_{i=1}^n ζ_i^l s_i^out δ_{p_i,k} ≤ ξ_k^d s^{out,k}
do
    i ∼ Ĩ^k                                  ▷ Draw a firm index i from sector k.
    Delete i from Ĩ^k                        ▷ Remove firm index i from the index set.
    Draw a shock η ∼ {ζ_j | j ∈ I^k}          ▷ Draw a shock ζ_j from the empirical distribution of shocks of sector k.
    Update shock ζ_i^l ← min[1, ζ_i^l + η]    ▷ Update the shock of firm i with the additional drawn shock.
    if Ĩ^k = ∅ then                          ▷ If each firm has received a shock and the aggregate shock is still too small.
        Set Ĩ^k ← {i |p_i = k}               ▷ Fill up the index set again and continue to draw shocks.
    end if
end while
return ζ_i^l for i ∈ I^k .                   ▷ Return the shock vector and use it as input for Algorithm 2.
```

Rescaling of shocks. In a second step we find weights to rescale the shocks, ζ_i^l, such that the constraints in Eq. [S.13] hold exactly. The basic idea is to divide the firms in sector k into two groups. The first group contains firms that have a higher ratio of in-strength to out-strength than the empirical shock, i.e.,

$$\frac{s_i^{\mathrm{in}}}{s_i^{\mathrm{out}}} > \frac{\xi_k^{\mathrm{u}}\, s^{\mathrm{in},k}}{\xi_k^{\mathrm{d}}\, s^{\mathrm{out},k}}$$

We assign all firms i of sector k that fulfil this condition to the set $I^{\mathrm{in},k}$. The second group contains firms that have a higher ratio of out-strength to in-strength than the target shock, i.e.,

$$\frac{s_i^{\mathrm{out}}}{s_i^{\mathrm{in}}} > \frac{\xi_k^{\mathrm{d}}\, s^{\mathrm{out},k}}{\xi_k^{\mathrm{u}}\, s^{\mathrm{in},k}}$$

We assign all firms i of sector k that fulfil this condition to the set $I^{\mathrm{out},k}$. Edge cases having exactly the same ratio can be added to the group with fewer firms. Then, we define a rescaling factor for the in-strength 'heavy' firms, v^{in}, that rescales all ζ_i^l where $i \in I^{\mathrm{in},k}$, and a rescaling factor for the out-strength 'heavy' firms, v^{out}, that rescales all ζ_i^l where $i \in I^{\mathrm{out},k}$. If we increase v^{in} while leaving v^{out} untouched, the shock scenario, ζ^l, will result in a higher loss of in-strength relative to the loss out-strength of sector k and therefore a larger upstream shock relative to the size of the downstream shock. If we increase v^{out} while leaving v^{in} untouched, the shock scenario, ζ^l, will result in a higher loss of out-strength relative to the loss of in-strength to of sector k and therefore a larger downstream shock relative to the size of the upstream shock. Now we only need to determine the weights v^{in} and v^{out}, such that the first two constraints in problem statement [S.13] exactly hold.

In principle the weights v^{in} and v^{out} can be found by solving the following linear system of equations,

$$v^{\mathrm{in}} \sum_{i\in I^{\mathrm{in},k}} \zeta_i^l\, s_i^{\mathrm{in}} \quad + \quad v^{\mathrm{out}} \sum_{i\in I^{\mathrm{out},k}} \zeta_i^l\, s_i^{\mathrm{in}} = \xi_k^{\mathrm{u}}\, s^{\mathrm{in},k} \quad , \tag{S.15}$$

$$v^{\mathrm{in}} \sum_{i\in I^{\mathrm{in},k}} \zeta_i^l\, s_i^{\mathrm{out}} \quad + \quad v^{\mathrm{out}} \sum_{i\in I^{\mathrm{out},k}} \zeta_i^l\, s_i^{\mathrm{out}} = \xi_k^{\mathrm{d}}\, s^{\mathrm{out},k} \quad . \tag{S.16}$$

The linear system [S.15-S.16] can be written in standard matrix form as

$$Av = \xi_k\, s^k \quad , \tag{S.17}$$

where $v = (v^{\mathrm{in}}, v^{\mathrm{out}})^\top$, $\xi_k\, s^k = (\xi_k^{\mathrm{u}}\, s^{\mathrm{in},k}, \xi_k^{\mathrm{d}}\, s^{\mathrm{out},k})$,

$$A_{11} = \sum_{i\in I^{\mathrm{in},k}} \zeta_i^l\, s_i^{\mathrm{in}} \quad ,$$

$$A_{12} = \sum_{i\in I^{\mathrm{out},k}} \zeta_i^l\, s_i^{\mathrm{in}} \quad ,$$

$$A_{21} = \sum_{i\in I^{\mathrm{in},k}} \zeta_i^l\, s_i^{\mathrm{out}} \quad ,$$

and

$$A_{22} = \sum_{i \in I^{\text{out},k}} \zeta_i^l \; s_i^{\text{out}} \quad .$$

The system is not always directly solvable for a given vector, ζ^l, that results from Algorithm 1.

In Algorithm 2 we show how to find the rescaling weights, $v = (v^{\text{in}}, v^{\text{out}})^\top$, for a given ζ^l. For each firm in industry k, i.e., the set $I^k = \{i \mid p_i = k\}$, we initialize the algorithm with the elements from the shock vector, ζ_i^l, that results from Algorithm 1. Then, we calculate the size of the violation of the first two constraints in problem statement [S.13], i.e. the distance to the targeted upstream shock size, $o^{\text{in}} \leftarrow \left| \sum_{i=1}^n \zeta_i^l \; s_i^{\text{in}} \; \delta_{p_i,k} - \xi_k^{\text{u}} \, s^{\text{in},k} \right|$ and the distance to the targeted downstream shock size, $o^{\text{out}} \leftarrow \left| \sum_{i=1}^n \zeta_i^l \; s_i^{\text{out}} \; \delta_{p_i,k} - \xi_k^{\text{d}} \, s^{\text{out},k} \right|$, where —.— denotes the absolute value. We define the "available for rescaling" indicator vector, d, where $d_i = 0$ indicates that the shock, ζ_i^l, can be rescaled, and $d_i = 1$ indicates that it can not be rescaled, because, ζ_i^l, was scaled above 1 in a previous iteration. Initially we set $d_i \leftarrow 0 \; \forall \; i \in I^k$, i.e. all firm shocks can initially be rescaled.

We continue the following calculations until the distance to the targeted upstream and downstream shock becomes smaller than a threshold ϵ, i.e. the algorithm stops when $\left(o^{\text{in}} \leq \epsilon\right)$ and $\left(o^{\text{out}} \leq \epsilon\right)$. We set the parameter epsilon to 0.01, such that in absolute monetary terms the difference in shocks becomes smaller than 10 Forint (approx 0.025 Euros).

First, we calculate the remaining target shock size, $b = (b^{\text{in}}, b^{\text{out}})$. b is the respective upstream or downstream shock target, $(\xi_k^{\text{u}}, \xi_k^{\text{d}})$, reduced by the respective in-strength or out-strength of firms that are not available for rescaling anymore. $b^{\text{in}} \leftarrow \left(\xi_k^{\text{u}} \, s^{\text{in},k} \; - \; \sum_{i=1}^n d_i \; s_i^{\text{in}}\right)$ specifies the size of the targeted in-strength shock that remains after deducting the in-strength of firms that received already a 100% shock, i.e., where $\zeta_i^l = 1$ and therefore where $d_i = 1$. $b^{\text{out}} \leftarrow \left(\xi_k^{\text{d}} \, s^{\text{out},k} \; - \; \sum_{i=1}^n d_i \; s_i^{\text{out}}\right)$ specifies the size of the targeted out-strength shock that remains after deducting the out-strength of firms that received already a 100% shock, i.e., where $\zeta_i^l = 1$ and therefore $d_i = 1$. The variables b^{in} and b^{out} need to be calculated in every iteration, because the change in the remaining target shock size, b, affects which firms belong to the set of "in-strength-heavy" firms and the set of "out-strength-heavy" firms. Hence, we update these two sets by setting $I^{\text{in},k}$ to include all firms i where $\frac{s_i^{\text{in}}}{s_i^{\text{out}}} > \frac{b^{\text{in}}}{b^{\text{out}}}$ and $I^{\text{out},k}$ to include all i where $\frac{s_i^{\text{out}}}{s_i^{\text{in}}} > \frac{b^{\text{out}}}{b^{\text{in}}}$. Edge cases can again be added to the group with fewer firms.

Next, we need to update the values of the coefficient matrix A. The values are updated, because firms that have received already a full shock ($d_i = 1$) are not considered anymore for rescaling, i.e. we sum only over firms where $d_i = 0$. We calculate $A_{11} = \sum_{i \in I^{\text{in},k}} \zeta_i^l \; s_i^{\text{in}} \; \mathbb{I}_{(d_i=0)}$, $A_{12} = \sum_{i \in I^{\text{out},k}} \zeta_i^l \; s_i^{\text{in}} \mathbb{I}_{(d_i=0)}$, $A_{21} = \sum_{i \in I^{\text{in},k}} \zeta_i^l \; s_i^{\text{out}} \mathbb{I}_{(d_i=0)}$, and $A_{22} = \sum_{i \in I^{\text{out},k}} \zeta_i^l \; s_i^{\text{out}} \mathbb{I}_{(d_i=0)}$. $\mathbb{I}_{(d_i=0)}$ is the indicator variable that is one if firm i can be rescaled and zero if firm i can not be rescaled anymore. The system has a solution when the rank of A has the same rank as the matrix $(A|b)$.

We list the four cases when the shocks, ζ_i^l, lead to a violation of the rank condition in matrix, A. First, if no firm i that has positive in-strength and is available for rescaling, (i.e., where $d_i = 0$), receives a shock, then the first row would be zero. Further, if additionally $b^{\text{in}} > 0$ the system has no solution. We can remedy this case by drawing a new shock for a firm that has previously not received a shock and has positive in-strength. Second, if no firm that has positive out-strength and is available for rescaling, (i.e., where $d_i = 0$), receives a shock, then the second row would be zero. Further, if additionally $b_k^{\text{out}} > 0$ the system has no solution. We can remedy this case by drawing a new shock for a firm that has previously not received a shock and has positive in-strength. These two cases do not happen with the initially drawn shocks, ζ_i^l, because of the condition in the while statement of Algorithm 1, but they can occur during the adjustment procedure in Algorithm 2, because the summations depend on the indicator variable d_i. Third, if no firm from the group of high in- to out-strength ratio, $I^{\text{in},k}$, receives a shock, then the first column of A is zero, which usually leads to an unsolvable system. We can remedy this case by drawing a new shock for a firm, i, that has previously not received a shock, $\zeta_i^l = 0$, and belongs to the set $I^{\text{in},k}$. Fourth, if no firm from the group of high out- to in-strength ratio, $I^{\text{out},k}$, receives a shock, then the second column of A is zero, which usually leads to an unsolvable system. We can remedy this case by drawing a new shock for a firm, i, that has previously not received a shock, $\zeta_i^l = 0$, and belongs to the set $I^{\text{out},k}$. The last two cases can occur since we do not specifically avoid them in Algorithm 1. If an additional shock was drawn, the matrix A needs to be updated again.

Next, we can solve the linear system of equations $Av = b$, by computing the generalized inverse, $A^\dagger$, of A and set $v \leftarrow A^\dagger b$. Then, we rescale the the elements of the shock vector ζ^l, in the following way. For the firms, i, that belong to the "in-strength-heavy" group, $i \in I^{\text{in},k}$, and are still available for rescaling, (where $d_i = 0$), we set $\zeta_i^l \leftarrow v^{\text{in}} \, \zeta_i^l$. For the firms, i, that belong to the "out-strength-heavy" group, $i \in I^{\text{out},k}$, and are still available for rescaling, $d_i = 0$, we set $\zeta_i^l \leftarrow v^{\text{out}} \, \zeta_i^l$. Then, we update the indicator variable by setting $d_i \leftarrow 1$ for all i with $\zeta_i^l > 0$. To ensure that shocks are not larger than one we take the maximum with 1, i.e. we set $\zeta_i^l \leftarrow \min[\zeta_i^l, \; 1]$. Finally, we update the distance to the target shock, o^{in} and o^{out}.

We have implemented algorithm 2 sufficiently fast to sample the 1,000 shocks for each of the approx. 245,000 firms within a few hours. Note that the common rescaling of many firm shocks at the same time with the same factors v might not lead to a full traversing of the space of all possible firm-level shocks that are consistent with our sampling problem [S.13]. This means that in practice for one specific industry-level shock the heterogeneity of production losses computed on the firm-level could be even larger. We have checked that the resulting shocks are uncorrelated on the firm-level and perfectly correlated (identical) when aggregated to the industry-level.

Algorithm 2 Rescaling weights for shocks of firms in industry k

1: set $I^k = \{i \,|p_i = k\}$
2: initialize with ζ_i^l for $i \in I^k$
3: set $o^{\text{in}} \leftarrow \left| \sum_{i \in I^k} \zeta_i^l \, s_i^{\text{in}} - \xi_k^{\text{u}} \, s^{\text{in},k} \right|$ and $o^{\text{out}} \leftarrow \left| \sum_{i \in I^k} \zeta_i^l \, s_i^{\text{out}} - \xi_k^{\text{d}} \, s^{\text{out},k} \right|$ ▷ Calculate the distance from the targeted shock.
4: set $d_i \leftarrow 0 \; \forall \; i \in I^k$ ▷ All shocks, $\zeta_i^l \; \forall i \in I^k$, are available for rescaling.
5: **while** $\left(o^{\text{in}} > \epsilon\right)$ and $\left(o^{\text{out}} > \epsilon\right)$ **do** ▷ Iterate until the distance to target up- and downstream shock size is small.
6: set $b \leftarrow \left(\xi_k^{\text{u}} \, s^{\text{in},k} - \sum_{i=1}^n d_i \, s_i^{\text{in}} \,,\; \xi_k^{\text{d}} \, s^{\text{out},k} - \sum_{i=1}^n d_i \, s_i^{\text{out}}\right)$ ▷ Calculate the remaining absolute shock that is left after deducting strength of fully scaled up firms i where $d_i = 1$.
7: Set $I^{\text{in},k}$ to include all i where $\frac{s_i^{\text{in}}}{s_i^{\text{out}}} > \frac{b^{\text{in}}}{b^{\text{out}}}$ ▷ Update "in-strength-heavy" group.
8: Set $I^{\text{out},k}$ to include all i where $\frac{s_i^{\text{out}}}{s_i^{\text{in}}} > \frac{b^{\text{out}}}{b^{\text{in}}}$ ▷ Update "out-strength-heavy" group.
9: calculate $A_{11} = \sum_{i \in I^{\text{in},k}} \zeta_i^l \, s_i^{\text{in}} \, \mathbb{I}_{(d_i=0)}$,
10: calculate $A_{12} = \sum_{i \in I^{\text{out},k}} \zeta_i^l \, s_i^{\text{in}} \mathbb{I}_{(d_i=0)}$,
11: calculate $A_{21} = \sum_{i \in I^{\text{in},k}} \zeta_i^l \, s_i^{\text{out}} \mathbb{I}_{(d_i=0)}$
12: calculate $A_{22} = \sum_{i \in I^{\text{out},k}} \zeta_i^l \, s_i^{\text{out}} \mathbb{I}_{(d_i=0)}$
13: **if** $A_{1.} = (0,0)$ **then** sample i where $\zeta_i^l = 0$, $s_i^{\text{in}} > 0$ and $d_i = 0$ and set $\zeta_i^l \sim U[0,1]$; recalculate lines 9-12
14: **end if**
15: **if** $A_{2.} = (0,0)$ **then** sample i where $\zeta_i^l = 0$, $s_i^{\text{out}} > 0$ and $d_i = 0$ and set $\zeta_i^l \sim U[0,1]$; recalculate lines 9-12
16: **end if**
17: **if** $A_{.1} = (0,0)^\top$ **then** sample i where $\zeta_i^l = 0$, $i \in I^{\text{in},k}$ and $d_i = 0$ and set $\zeta_i^l \sim U[0,1]$; recalculate lines 9-12
18: **end if**
19: **if** $A_{.2} = (0,0)^\top$ **then** sample i where $\zeta_i^l = 0$, $i \in I^{\text{out},k}$ and $d_i = 0$ and set $\zeta_i^l \sim U[0,1]$; recalculate lines 9-12
20: **end if**
21: Calculate the generalized inverse $A^\dagger$, of coefficient matrix A
22: set $v \leftarrow A^\dagger b$ ▷ Calculate the rescaling coefficients v.
23: set $\zeta_i^l \leftarrow v^{\text{in}} \, \zeta_i^l$ for $i \in I^{\text{in},k}$ and $d_i = 0$ ▷ Rescale shocks of "in-heavy" firms.
24: set $\zeta_i^l \leftarrow v^{\text{out}} \, \zeta_i^l$ for $i \in I^{\text{out},k}$ and $d_i = 0$ ▷ Rescale shocks of "out-heavy" firms.
25: set $d_i = 1$ for all i with $\zeta_i^l > 0$
26: set $\zeta_i^l \leftarrow \max[0, \min[\zeta_i^l, \; 1]]$
27: set $o^{\text{in}} \leftarrow \left| \sum_{i \in I^k} \zeta_i^l \, s_i^{\text{in}} - \xi_k^{\text{u}} \, s^{\text{in},k} \right|$ and $o^{\text{out}} \leftarrow \left| \sum_{i \in I^k} \zeta_i^l \, s_i^{\text{out}} - \xi_k^{\text{d}} \, s^{\text{out},k} \right|$ ▷ Update the distance from the targeted shock.
28: **end while**
29: **end while**
30: **return** ζ_i^l

SI Section 9. Details on industry-level production losses

In this section we give an overview of the production losses for all NACE2 industries. Table S1 compares theindustry-specific production losses between firm-level production network (FPN) based loss estimates and industry-level production network (IPN) based loss estimates for the NACE2 classes A01 to F43. The first column shows the NACE2 code for which production losses are compared across firm-level and industry-level production losses. The second and third column show the aggregation of the initial shock ζ, to the NACE2 level, into the up-stream shock, ξ^{u}, and the downstream shock, ξ^{d}, respectively. The fourth column shows the FPN based production losses, $L^k_{\mathrm{firm}}(\psi)$, for the labor shock, ψ (red 'x' symbols in Fig. 5). The fifth column shows the average FPN-based production losses, $\mathbb{E}[L^k_{\mathrm{firm}}(\Psi)]$, corresponding to the 1,000 synthetic firm-level shock scenarios, Ψ (mean of the boxplots in Fig. 5). The sixth column shows the IPN-based production losses, $L^k_{\mathrm{ind.}}(\phi)$, corresponding to the aggregated industry-level shock scenarios, ϕ (blue '+' symbols in Fig. 5). The seventh column shows the mean deviation, $\mathbb{E}\big[\frac{L^k_{\mathrm{ind.}}(\phi)}{L^k_{\mathrm{firm}}(\Psi)} - 1\big]$, of the industry-level production losses, $L^k_{\mathrm{ind.}}(\phi)$, from the firm-level production losses, $L^k_{\mathrm{firm}}(\Psi)$, across the 1,000 different firm-level shock scenarios Ψ. Note that when aggregated, all firm-level initial shocks, Ψ, are all identical to the industry-level shock, ϕ, that corresponds to the COVID-19 shock ψ. Table S2 compares theindustry-specific production losses between firm-level production network (FPN) based loss estimates and industry-level production network (IPN) based loss estimates for NACE2 classes from G45 to U99, incl. the fictional category where the NACE class is not available. The columns are as in Table S1. Note that on the aggregate level the shocks, Ψ, ψ, amount to 4.86% of overall out-strength and 4.58% of overall in-strength.

Table S1. Comparison of industry-specific production losses between firm-level production network (FPN) based loss estimates and industry-level production network (IPN) based loss estimates for NACE2 classes from A01 to F43.

NACE2	ind. shock, ξ^{u}	ind. shock, ξ^{d}	FPN-loss, $L^k_{\mathrm{firm}}(\psi)$	avg. FPN-loss, $L^k_{\mathrm{firm}}(\Psi)$	IPN-loss, $L_{\mathrm{ind.}}(\phi)$	avg. IPN/FPN
A01	0.03	0.03	0.12	0.12	0.11	-0.05
A02	0.06	0.07	0.14	0.16	0.13	-0.19
A03	0.04	0.04	0.12	0.10	0.08	-0.22
B05	0.02	0.16	0.17	0.23	0.16	-0.25
B06	0.00	0.00	0.11	0.12	0.08	-0.19
B07	0.05	0.05	0.08	0.10	0.09	0.12
B08	0.02	0.02	0.11	0.12	0.13	0.12
B09	0.08	0.05	0.11	0.13	0.08	-0.31
C10	0.03	0.03	0.12	0.10	0.11	0.05
C11	0.03	0.02	0.12	0.11	0.10	0.02
C12	0.00	0.01	0.07	0.06	0.05	0.02
C13	0.05	0.08	0.13	0.13	0.08	-0.38
C14	0.10	0.10	0.18	0.16	0.10	-0.36
C15	0.10	0.17	0.18	0.19	0.17	-0.10
C16	0.05	0.04	0.10	0.11	0.09	-0.17
C17	0.01	0.02	0.11	0.10	0.09	-0.08
C18	0.07	0.09	0.13	0.14	0.10	-0.27
C19	0.00	0.00	0.07	0.04	0.07	0.87
C20	0.02	0.11	0.20	0.20	0.11	-0.42
C21	0.01	0.01	0.12	0.11	0.09	-0.11
C22	0.03	0.04	0.12	0.12	0.07	-0.38
C23	0.02	0.03	0.11	0.12	0.11	-0.03
C24	0.04	0.06	0.13	0.11	0.07	-0.37
C25	0.05	0.05	0.10	0.10	0.05	-0.50
C26	0.03	0.03	0.09	0.08	0.03	-0.59
C27	0.03	0.03	0.09	0.09	0.06	-0.34
C28	0.04	0.03	0.08	0.08	0.04	-0.53
C29	0.02	0.03	0.09	0.09	0.07	-0.27
C30	0.06	0.01	0.12	0.09	0.07	-0.17
C31	0.09	0.09	0.13	0.14	0.09	-0.36
C32	0.04	0.06	0.10	0.11	0.09	-0.16
C33	0.03	0.04	0.09	0.10	0.08	-0.20
D35	0.10	0.09	0.21	0.22	0.12	-0.45
E36	0.00	0.00	0.12	0.11	0.15	0.36
E37	0.01	0.03	0.20	0.16	0.16	0.15
E38	0.04	0.04	0.12	0.12	0.14	0.12
E39	0.05	0.05	0.10	0.10	0.14	0.42
F41	0.06	0.07	0.13	0.14	0.09	-0.37
F42	0.03	0.04	0.10	0.11	0.11	0.03
F43	0.06	0.06	0.12	0.12	0.11	-0.11

Table S2. Comparison ofindustry-specific production losses between firm-level production network (FPN) based estimates and industry-level production network (IPN) based estimates for NACE2 classes from G45 to U99, incl. the fictional category where the NACE class is not available.

NACE2	ind. shock, ξ^{u}	ind. shock, ξ^{d}	FPN-loss, $L^k_{\mathrm{firm}}(\psi)$	avg. FPN-loss, $L^k_{\mathrm{firm}}(\Psi)$	IPN-loss, $L_{\mathrm{ind.}}(\phi)$	avg. IPN/FPN
G45	0.05	0.04	0.09	0.11	0.10	-0.09
G46	0.04	0.04	0.08	0.09	0.08	-0.12
G47	0.04	0.06	0.14	0.14	0.10	-0.26
H49	0.05	0.06	0.13	0.14	0.15	0.08
H50	0.08	0.15	0.19	0.19	0.15	-0.23
H51	0.03	0.03	0.07	0.05	0.03	-0.35
H52	0.03	0.04	0.14	0.16	0.11	-0.33
H53	0.02	0.03	0.11	0.11	0.15	0.35
I55	0.17	0.15	0.21	0.20	0.17	-0.16
I56	0.20	0.20	0.23	0.24	0.20	-0.15
J58	0.04	0.03	0.17	0.15	0.07	-0.51
J59	0.06	0.06	0.12	0.12	0.09	-0.25
J60	0.02	0.01	0.13	0.13	0.16	0.21
J61	0.03	0.02	0.15	0.17	0.15	-0.04
J62	0.05	0.06	0.11	0.12	0.15	0.24
J63	0.07	0.03	0.20	0.17	0.08	-0.48
K64	0.03	0.03	0.09	0.10	0.07	-0.22
K65	0.00	0.00	0.01	0.03	0.01	-0.32
K66	0.01	0.01	0.06	0.05	0.13	1.50
L68	0.09	0.05	0.10	0.11	0.11	0.07
M69	0.03	0.04	0.14	0.13	0.13	-0.01
M70	0.07	0.07	0.14	0.14	0.12	-0.13
M71	0.04	0.04	0.09	0.10	0.10	0.04
M72	0.04	0.03	0.06	0.07	0.08	0.29
M73	0.05	0.05	0.14	0.14	0.13	-0.08
M74	0.05	0.05	0.10	0.10	0.12	0.23
M75	0.02	0.05	0.16	0.09	0.11	0.32
N77	0.05	0.04	0.10	0.10	0.14	0.41
N78	0.27	0.17	0.21	0.20	0.27	0.34
N79	0.14	0.19	0.22	0.22	0.19	-0.13
N80	0.07	0.08	0.15	0.16	0.17	0.04
N81	0.07	0.10	0.16	0.17	0.16	-0.05
N82	0.05	0.08	0.15	0.16	0.14	-0.13
O84	0.01	0.01	0.08	0.07	0.08	0.12
P85	0.07	0.10	0.15	0.14	0.10	-0.32
Q86	0.10	0.06	0.10	0.10	0.10	-0.02
Q87	0.05	0.01	0.03	0.05	0.05	0.42
Q88	0.01	0.04	0.08	0.09	0.15	0.80
R90	0.07	0.07	0.14	0.14	0.12	-0.15
R91	0.02	0.03	0.07	0.08	0.14	0.83
R92	0.12	0.16	0.17	0.20	0.16	-0.20
R93	0.12	0.10	0.18	0.17	0.13	-0.23
S94	0.05	0.04	0.09	0.09	0.15	0.65
S95	0.05	0.04	0.07	0.09	0.11	0.34
S96	0.09	0.10	0.16	0.17	0.13	-0.19
U99	0.12	0.00	0.00	0.00	0.12	Inf
NA	0.05	0.07	0.12	0.12	0.08	-0.30

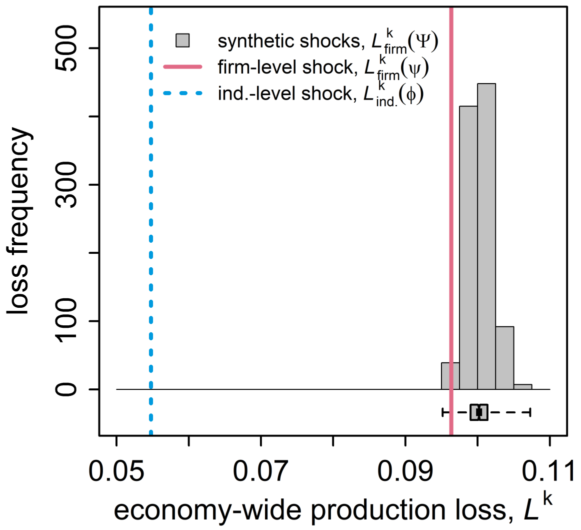

Figure S16. Economy-wide production losses, L, obtained from an empirically calibrated and 1,000 synthetic COVID-19 shocks propagating *linearly* on the aggregated industry-level production network, IPN, (blue dashed line) and on the firm-level production network, FPN, (red line, histogram). The FPN and IPN correspond to the production network of Hungary in 2019; the firm-level shock, ψ, correspond to firms reducing their production level proportional to their reduction in employees between January and May 2020, and are taken from monthly firm-level labor data. The NACE2 level shock, ϕ, is the aggregation of ψ. The 1,000 synthetic shocks, Ψ, are sampled such that (when they are aggregated to the NACE2 level) they all have the same size as ϕ.The empirically calibrated shock, ψ, yields a FPN-based loss, $L_{\text{firm}}(\psi)$, of 9.6% (red line). The synthetic shocks yield a distribution of FPN-based production losses, $L_{\text{firm}}(\Psi)$, ranging from 9.5% to 10.7% of national output (histogram). The median is 10% (see boxplot). As a reference, the Hungarian GDP declined by 14.2% in Q2 2020. Note that for the IPN all realizations, Ψ, result in the same production loss, $L_{\text{ind.}}(\phi)$, of 5.5%, by construction. The aggregation to the IPN causes a substantial underestimation of the FPN-based production losses.

SI Section 10. Results on linear shock propagation

In this section we show how production losses propagate differently on the firm-level and industry-level production network, when all firms and industries have only linear production functions. As pointed out in Eq. [7], each firm i is equipped with a generalized Leontief production function (GLPF), which is defined as

$$x_i = \min\left[\min_{k\in\mathcal{I}_i^{\text{es}}}\left[\frac{1}{\alpha_{ik}}\Pi_{ik}\right],\ \beta_i + \frac{1}{\alpha_i}\sum_{k\in\mathcal{I}_i^{\text{ne}}}\Pi_{ik},\ \frac{1}{\alpha_{l_i}}l_i,\ \frac{1}{\alpha_{c_i}}c_i\right], \tag{S.18}$$

and where $\mathcal{I}_i^{\text{es}}$ is the set of essential inputs, $\mathcal{I}_i^{\text{ne}}$ is the set of non-essential inputs of firm i. The linear production function is a special case of the GLPF where all inputs are in the set of non-essential inputs, $\mathcal{I}_i^{\text{ne}}$. We simulate the shocks when for all firms i all inputs, $k \in \mathcal{I}_i^{\text{ne}}$ belong to, $k \in \{1, 2, \dots, m\}$.

We show the estimation errors for network wide production losses from simulating the shock propagation on the IPN, Z, instead on the FPN, W. Fig. S16 shows the distribution of network wide production losses, $L_{\text{firm}}(\psi^l)$, in response to the 1,000 synthetic COVID-19 shock scenarios Ψ (defined in the maintext) as histogram and boxplot; loss bins, $L_{\text{firm}}(\psi^l)$, are on the x-axis and frequency of the losses in the respective bins on the y-axis. The variability of losses is economically substantial and ranges from 9.51% to 10.73% — a factor of 1.13. The median and mean losses are 10% each. The variation is substantially smaller than for case with the GLPF shown in Fig. 4 with losses differing by a factor of up to 1.46 across different shocks. Note again that the GDP growth in Hungary for Q2 2020 was -14.2%, indicating a realistic order of magnitude, but a substantial underestimation. Note again that the initial shocks all have the same monetary size and are identical at the industry-level, i.e. the variation of losses is merely due to the fact that different firms within sectors are initially shocked. The distribution is slightly right skewed with a right tail of larger losses. The right tail is substantially smaller than for the GLPF case. The production loss, $L_{\text{firm}}(\psi) = 9.6\%$, corresponding to the labor shock, ψ, (red vertical solid line) lies below the median of the loss distribution.

The IPN based production losses, $L_{\text{ind.}}(\phi)$, are shown as vertical blue dashed line. As in the main text, firm-level shocks are by construction identical when aggregated to the NACE2 level, each of the 1,000 shock scenarios leads to exactly the same production loss of 5.5% when propagating on the NACE2 level IPN, Z. Interestingly, the IPN estimated production losses, $L_{\text{ind.}}(\phi)$, are substantially smaller than the distribution of FPN estimated production losses $L_{\text{firm}}(\Psi)$. Therefore, the aggregated network, Z not only can not capture the variation of production losses on the firm-level network, W, but the overall level of shock propagation is underestimated substantially. To quantify the error of estimating the FPN based production loss, $L_{\text{firm}}(\Psi)$, with the corresponding IPN based production loss, $L_{\text{ind.}}(\phi)$, we calculate the mean absolute error (deviation). We find that the average estimation error is -45.35% ($\mathbb{E}\big[\frac{L_{\text{ind.}}(\phi)}{L_{\text{firm}}(\Psi)} - 1\big]$). For the Hungarian production network and the initial shocks, industry-level network shock propagation tends to substantially and systematically underestimate losses from firm-level shock propagation also when production functions are linear.

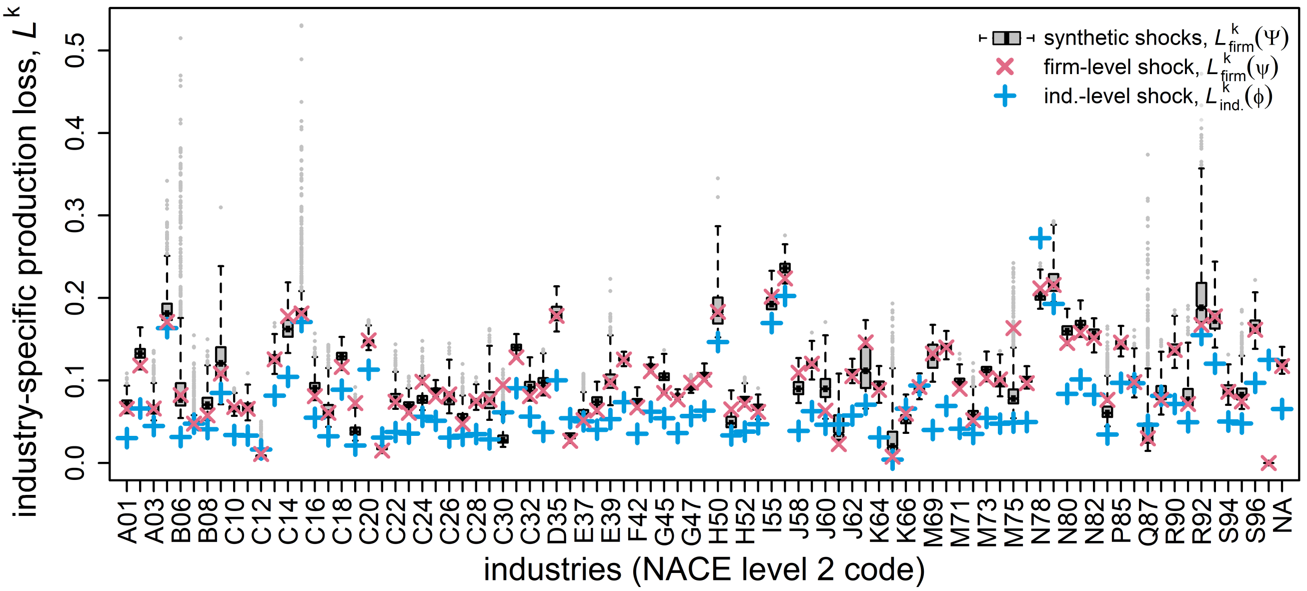

Figure S17. Comparison of industry-specific production losses, L^k, obtained from an empirically calibrated and 1,000 synthetic COVID-19 shocks propagating *linearly* on the aggregated industry-level production network, IPN, (blue '+'es) and on the firm-level production network, FPN, (red 'x'es, boxplots). For most industries the FPN-based production losses, $L^k_{\mathrm{firm}}(\Psi)$, (boxplots) vary substantially for few strongly across the synthetic shocks even though shocks have the same size on industry-level. Shock propagation on the industry-level (blue '+'es) can not capture this variation. IPN-based production-losses typically under-estimate the FPN based production losses significantly, on average by about 31.1%.

Fig. 5 shows the distribution of industry specific production losses, $L^k_{\mathrm{firm}}(\Psi)$, in response to the 1,000 synthetic COVID-19 shock scenarios, Ψ, as boxplots. Each boxplot corresponds to an industry, k, with the NACE2 code on the x-axis; the y-axis denotes the losses, $L^k_{\mathrm{firm}}(\Psi)$, of the respective NACE2 codes. The mean overindustry-specific median (mean) losses is 10% (10.3%). The red 'x' symbols represent the production losses, $L^k_{\mathrm{firm}}(\psi)$, corresponding to the original labor shock, ψ and lie within the boxes. We clearly see that for many industries remaining production levels vary strongly across initial shocks and the level of variation is very different across industries. The production loss distributions are obviously right skewed — indicated by extended upper vertical lines (whiskers) — for all but two industries (H53, N82). Few industries (B05, B06, C15, K65, M75, Q87, and R92) have a substantial amount of outliers (grey dots) that lie outside of 3 times the interquartile range. The minimum and maximum values can differ by factors of up to 9.5 (B06), 7.7 (C12), 5.9 (C30), 5.1 (J61), 41.1 (K65), or 25.8 (Q87). The median (mean) ratios of maximum to minimum loss is 1.27 (1.58). Again, these large deviations do not stem from different sizes of initial shocks, but affecting different firms within industries. Note that for some sectors the factors, representing the relative variation (maximum loss / minimum loss), are even higher for the case of only linear shock propagation. This is due to the fact that the minimum of the losses are smaller for the linear shock propagation, but the maximum losses are not affected by the non-linearities of the GLPF, i.e. ratios are larger. Fig. 5 shows that the IPN basedindustry-specific production losses, $L^k_{\mathrm{ind.}}(\phi)$, (blue '+' symbols) deviate even stronger from the FPN based losses than for network wide losses. The sectors where IPN based shock propagation underestimates output losses the most are C6 (-62.6%), C26 (-60.7%), C29 (-62%), C33 (-61.5%), K64 (-66.8%), K65 (-75.5%), and M69 (-68.6%) with negative average relative deviation in parenthesis. Overestimation of losses are highest for sectors, C12 (95.3%), C21 (70%), E36 (66.3%), and 87 (46%). The average across the mean *absolute* deviation of industries is 31.1%.

SI Section 11. Summary statistics of the production network

Summary statistics for the production network in 2019 containing only stable links (i.e., a link is present if at least two transactions occurred in two different quarters between the respecitve seller and buyer). The columns of Table S3 show the summary statistics of the in-degree, out-degree, in-strengh, out-strength, and link weight distributions of the network, respectively. Monetary units are in million Forint; 81,125 firms have no in-links and 51,679 firms have no out-links above the reporting threshold.

References

1. William P Jones and George W Furnas. Pictures of relevance: A geometric analysis of similarity measures. *Journal of the American society for information science*, 38(6):420–442, 1987.
2. MK Vijaymeena and K Kavitha. A survey on similarity measures in text mining. *Machine Learning and Applications: An International Journal*, 3(2):19–28, 2016.
3. Emmanuel Dhyne, Ayumu Ken Kikkawa, Magne Mogstad, and Felix Tintelnot. Trade and domestic production networks. *The Review of Economic Studies*, 88(2):643–668, 2021.

	in-degree	out-degree	in-strength in millions	out-strength in millions	link weight in millions
Minimum	0	0	0.00	0.00	0.00
25% Quantile	0	1	0.00	1.15	2.17
Median	1	1	5.26	10.23	6.07
Mean	4.54	4.54	230.42	230.42	50.78
75% Quantile	3	3	36.52	46.22	18.05
99% Quantile	57	56	2,605.84	2,771.30	565.11
99.9% Quantile	220	239	25,532.70	27,046.34	4,310.69
99.99% Quantile	643	947	220,741.28	142,470.25	27,272.62

Table S3. The columns show the summary statistics for the in-degree, out-degree, in-strength, out-strength, and link weight distributions of the production network in 2019, respectively. In-strength, out-strength, and link-weights are given in million Forint.
